# Rooting the tree of life by transition analyses

**DOI:** 10.1186/1745-6150-1-19

**Published:** 2006-07-11

**Authors:** Thomas Cavalier-Smith

**Affiliations:** 1 Department of Zoology, University of Oxford, South Parks Road, Oxford, OX1 3PS, UK

## Abstract

**Background:**

Despite great advances in clarifying the family tree of life, it is still not agreed where its root is or what properties the most ancient cells possessed – the most difficult problems in phylogeny. Protein paralogue trees can theoretically place the root, but are contradictory because of tree-reconstruction artefacts or poor resolution; ribosome-related and DNA-handling enzymes suggested one between neomura (eukaryotes plus archaebacteria) and eubacteria, whereas metabolic enzymes often place it within eubacteria but in contradictory places. Palaeontology shows that eubacteria are much more ancient than eukaryotes, and, together with phylogenetic evidence that archaebacteria are sisters not ancestral to eukaryotes, implies that the root is not within the neomura. Transition analysis, involving comparative/developmental and selective arguments, can polarize major transitions and thereby systematically exclude the root from major clades possessing derived characters and thus locate it; previously the 20 shared neomuran characters were thus argued to be derived, but whether the root was within eubacteria or between them and archaebacteria remained controversial.

**Results:**

I analyze 13 major transitions within eubacteria, showing how they can all be congruently polarized. I infer the first fully resolved prokaryote tree, with a basal stem comprising the new infrakingdom Glidobacteria (Chlorobacteria, Hadobacteria, Cyanobacteria), which is entirely non-flagellate and probably ancestrally had gliding motility, and two derived branches (Gracilicutes and Unibacteria/Eurybacteria) that diverged immediately following the origin of flagella. Proteasome evolution shows that the universal root is outside a clade comprising neomura and Actinomycetales (proteates), and thus lies within other eubacteria, contrary to a widespread assumption that it is between eubacteria and neomura. Cell wall and flagellar evolution independently locate the root outside Posibacteria (Actinobacteria and Endobacteria), and thus among negibacteria with two membranes. Posibacteria are derived from Eurybacteria and ancestral to neomura. RNA polymerase and other insertions strongly favour the monophyly of Gracilicutes (Proteobacteria, Planctobacteria, Sphingobacteria, Spirochaetes). Evolution of the negibacterial outer membrane places the root within Eobacteria (Hadobacteria and Chlorobacteria, both primitively without lipopolysaccharide): as all phyla possessing the outer membrane β-barrel protein Omp85 are highly probably derived, the root lies between them and Chlorobacteria, the only negibacteria without Omp85, or possibly within Chlorobacteria.

**Conclusion:**

Chlorobacteria are probably the oldest and Archaebacteria the youngest bacteria, with Posibacteria of intermediate age, requiring radical reassessment of dominant views of bacterial evolution. The last ancestor of all life was a eubacterium with acyl-ester membrane lipids, large genome, murein peptidoglycan walls, and fully developed eubacterial molecular biology and cell division. It was a non-flagellate negibacterium with two membranes, probably a photosynthetic green non-sulphur bacterium with relatively primitive secretory machinery, not a heterotrophic posibacterium with one membrane.

**Reviewers:**

This article was reviewed by John Logsdon, Purificación López-García and Eric Bapteste (nominated by Simonetta Gribaldo).

## Open peer review

Reviewed by John Logsdon, Purificación Lopez-García and Eric Bapteste (nominated by Simonetta Gribaldo). For the full reviews, please go to the Reviewers' comments section.

## Background

Correctly placing the root of the evolutionary tree of all life would enable us to deduce rigorously the major characteristics of the last common ancestor of life. It is probably the most difficult problem of all in phylogenetics, but not yet solved – contrary to widespread assumptions [1,2]. It is also most important to solve correctly because the result colours all interpretations of evolutionary history, influencing ideas of which features are primitive or derived and which branches are deeper and more ancient than others [1]. The wrong answer misleads profoundly in numerous ways. Establishing the root of a small part of the tree is more straightforward, yet often surprisingly difficult for organisms without plentiful fossils [3,4]. Usually the root of a subtree is located by comparisons with known outgroups. However, outgroups for the entire tree are air, rocks and water, not other organisms, vastly increasing the problem, which uniquely involves the origin of life – not just transitions between known types of organism. Here I explain how this seemingly intractable problem can be solved by supplementing standard molecular phylogenetic methods with the very same conceptual methods that were originally used to establish 'known outgroups' in well-defined parts of the tree, long before sequencing was invented. I then apply these methods comprehensively to establish far more closely than ever before where the root of the tree of life actually is.

I show here that, in conjunction with palaeontology and sequence trees, the methods of transition analysis and congruence testing demonstrate that archaebacteria are the youngest bacterial phylum and that the root lies within eubacteria, specifically among negibacteria of the superphylum Eobacteria, probably between Chlorobacteria and all other living organisms (Table 1 summarizes the prokaryotic nomenclature used here, which is slightly revised from previously [1], primarily by excluding Eurybacteria from Posibacteria). Chlorobacteria comprise photosynthetic 'non-sulphur' green bacteria like *Chloroflexus *and *Heliothrix*, some little-studied heterotrophs (e.g. *Thermomicrobium*, *Dehalococcoides*) and some apparently deeper-branching lineages known only from environmental DNA sequences and thus of unknown properties [1]. I use cladistic and transition analysis to provide the first rooted and fully resolved tree for all ten phyla of bacteria recognized here.

**Table 1:** The nomenclature and classification used here for prokaryotes (=Bacteria)

			Example genus
NEGIBACTERIA (subkingdom)			
Glidobacteria			
Eobacteria			
	Chlorobacteria*	Chloroflexi; green non-sulphur	*Chloroflexus*
	**Hadobacteria**	*Deinococcus*/*Thermus *group	*Thermus*
	**Cyanobacteria**		*Nostoc*
Gracilicutes			
	**Spirochaetae**	Spirochaetes	*Treponema*
	**Sphingobacteria**		
	Chlorobea	Chlorobi	*Chlorobium*
	Flavobacteria	CFB group + Fibrobacteres	*Cytophaga*
Exoflagellata			
	**Proteobacteria**		
	Rhodobacteria	α-, β-, γ-proteobacteria	*Escherichia*
	Thiobacteria	δ-, ε-proteobacteria + Aquificales	*Helicobacter*
	Geobacteria	Deferribacteres + Acidobacteria +	*Geovibrio*
	**Planctobacteria**	Planctomycetes + Chlamydiales +	*Pirellula*
**Eurybacteria**			
	Selenobacteria		*Sporomusa*
	Fusobacteria		*Fusobacterium*
	Togobacteria	Thermotogales	*Thermotoga*
UNIBACTERIA (subkingdom)			
**Posibacteria**			
	Endobacteria	low-GC Gram positives (incl. Mollicutes)	*Bacillus*
	Actinobacteria	high-GC Gram positives (e.g. Actinomycetales)	*Streptomyces*
**Archaebacteria**			
	Euryarchaeota	euryarchaeotes (e.g. methanogens)	*Halobacterium*
	Crenarchaeota	crenarchaeotes	*Sulfolobus*

* The 10 taxa shown in bold are ranked as phyla. A more detailed classification is given later in the paper, when I explain the small improvements over the previous system [1].

In addition to the taxa listed, three informal names are used for the following higher groups:

glycobacteria (a paraphyletic grade) = Cyanobacteria + Eurybacteria + Gracilicutes

proteates (a clade) = Actinomycetales + Archaebacteria + Eukaryota

neomura (a clade) = Archaebacteria + Eukaryota

I also provide new perspectives on the evolution of bacterial flagella and the cell envelope and conclude that the last common ancestor (cenancestor) of all life was a highly developed non-flagellate Gram-negative eubacterium with murein cell walls, acyl ester phospholipids, and probably non-oxygenic photosynthesis and gliding motility. It was more primitive than other eubacteria in probably lacking lipopolysaccharide, hopanoids, cytochrome b, catalase, the HslV ring protease homologue of proteasomes, spores, the machinery based on outer membrane (OM) protein Omp85 used by more advanced negibacteria to insert outer membrane proteins, type I, type II, and type III secretion mechanisms, and TonB-energized OM import systems. I briefly discuss implications of this novel rooting of the universal tree for understanding primordial cell biology and the history of life and its impact on global climate.

### The primacy of transition analysis

Classically three types of argument have been used to distinguish in-groups and out-groups. First, the fossil record. Among vertebrates, birds and mammals must be derived from reptiles, not vice versa, because reptile fossils are so much earlier. Likewise reptiles are derived from amphibians that were objectively earlier, amphibians from bony fish as fish are more ancient.

Second is transition analysis [5], which can often polarize major changes, showing that A went to B, not B to A. Thus, when birds originated, forelimbs previously used for walking were transformed into wings. We rule out the reverse by comparative/developmental and selective arguments. 18th century comparisons showed the structural and developmental homology of all pentadactyl limbs. Before palaeontology gave a time scale and evidence of direction it was obvious that wings were specializations of legs, not the reverse. Fore and hind limbs were clearly homologous throughout tetrapods; they must first have been essentially the same, as in amphibians and reptiles, not highly differentiated as in birds. It would be impossible mechanistically (developmentally or mutationally) to have evolved the very different bird wing and leg as the first tetrapod limbs – subsequent derivation of essentially similar reptilian five-toed legs from each would be equally improbable: that scenario would place birds at the tetrapod root; to become reptiles they would have to separate their fused trunk skeletons into discrete bones, convert feathers to scales, and evolve teeth. Such changes would be mechanistically complex, difficult, and of no selective advantage.

Transition analysis, if imaginative and critical, often clearly polarizes change unambiguously in the complete absence of a fossil record. Fossils are static and discontinuous; they do not show transitions or continuity directly and can be interpreted properly only by critical transition analysis, which is therefore the most fundamental way to polarize the direction of evolutionary change. For vertebrate evolution the fossil record is a valuable extra, inessential benefit. It is important to note that not all transitions can be clearly polarized when studied individually. Some evolutionary changes can in principle occur in either direction; evolutionary direction in such cases can only be established by reference to other changes that can be polarized and their relationship to the topology of the tree. It is the subset of changes that have a sufficient degree of complexity to allow unambiguous polarization that are of key importance for rooting trees. The key question that decides the utility of a particular character for this purpose is whether its evolution has enough evidence of directionality, which may be inherent in the process of evolutionary change itself or deducible by comparison between an evolved state and its putative precursors and knowledge of their phylogenetic distribution. Without evidence of directionality a character cannot be used clearly to polarize the tree.

I call the third approach congruence testing. One searches for congruence across major parts of the evolutionary tree between what analyses of individual transitions tell us, to ensure that the whole story is consistent; consistent historically and compatible with comparative morphology, genetics, developmental biology, and ecology. Thus in reptiles not only the ancestors of snakes but numerous different lizard groups lost limbs. Consistency across the whole tetrapod tree excludes its root from any group of limbless reptile. In unicellular organisms character losses have been equally confusing; yet though useful morphological characters are fewer, transition and congruence testing eventually enable losses to be identified and polarize transitions, especially by adding molecular cladistic characters [1,6]. Historically, biologists studying macroorganisms worked on many parts of the tree at once, using cross comparisons to hone arguments and criteria; such critical evaluation rejected discordant scenarios and subhypotheses. With congruence testing a serious mistake in one part of the tree may be revealed by incongruence with other parts. If two polarizations in different parts of the tree are incongruent (contradictory), then either the topology of the tree is incorrect or one of the polarizations is incorrect, and the source of the conflict can be sought for and at least one of the interpretations corrected in the light of the overall evidence from as many sources as possible. Usually it will be found that one of the lines of evidence is weaker than the others and has been given too much weight or is positively misleading or fundamentally misinterpreted. Search for congruence among multiple lines of evidence – the more diverse the better and resolving apparent contradictions by weighing up the evidence is not special to evolutionary biology but fundamental to all science. Its importance is easily overlooked by specialists familiar with only one field. Gaucher et al. [7] have rightly stressed that such an integrative approach, though recently unfashionable, is sorely needed in the face of the mass of new genomic data to suggest biologically well-grounded hypotheses to guide detailed experimental studies in the laboratory.

### Problems with sequence trees

Recent discussions about the root of the universal tree mostly fail to consider any palaeontological evidence or execute either transition analysis, or congruence testing and focus solely on sequence trees. Single-gene trees, notably of rRNA and unusually well-conserved proteins like cytochromes, RuBisCO and chaperones, have been valuable in clustering together relatively closely related organisms, especially if morphology was inadequate to establish their closest relatives (often because of character losses). Occasionally they made major breakthroughs, as in the recognition of Archaebacteria and Proteobacteria in prokaryotes and Cercozoa in protozoa [8]. Unfortunately, such trees have four serious limitations. First is limited resolution, especially for basal eukaryotes and prokaryotes, where branching order is almost totally unresolved and must be established otherwise. Second is pervasive systematic biases in evolutionary mode, which affect segments of the tree differentially causing some branches to be placed entirely incorrectly [2]; all sequence trees require testing and corroboration by other evidence. Such testing is sophisticated in the eukaryote part of the tree now [6], but for prokaryotes a regrettable tendency to take 16S rRNA trees as gospel truth and ignore other evidence persists; critical cladistic analyses are rare [1,9,10]. Thirdly, lateral gene transfer, commoner in bacteria than eukaryotes, but of uneven frequency, also places occasional branches incorrectly on single-gene trees [11,12]. Fourthly, single-gene trees are always unrooted, lacking inherent evidence of direction; any nucleotide can substitute reversibly for any other. These severe limitations of sequence trees emphatically do not mean that they are worthless. On the contrary, they are indispensable, but they must be interpreted critically and supplemented by cladistic, transition analysis and congruence testing, and by critical palaeontology, in order to produce a reliable and comprehensive picture. Some perceptive molecular biologists now appreciate the need to integrate sequence trees into the broader and time-based framework provided by palaeontology [7]. This synthetic approach to the history of life should become much more widespread [7].

### Paralogue rooting failed clearly to root the tree

**Figure 1:**
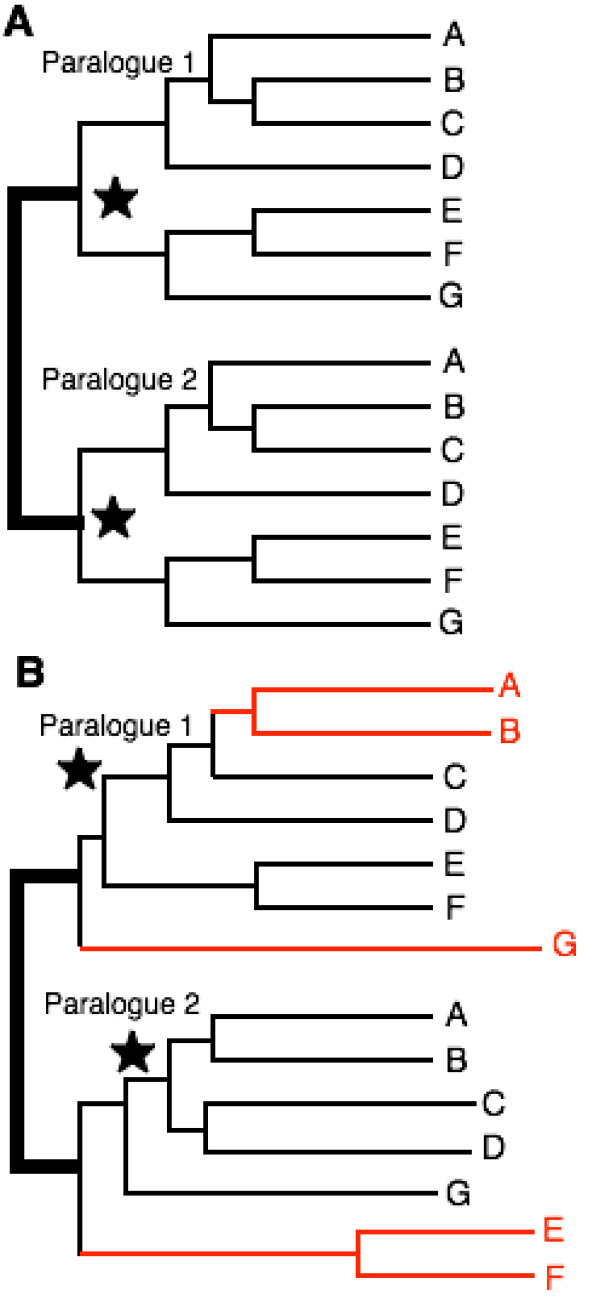
The logic and problems of paralogue rooting. In theory (A), two genes that arose from a single parent by duplication immediately prior to the common ancestor of the group under study should yield two identical trees joined together by a line (shown extra thick) between the roots (stars) of each tree. Letters are taxa. In practice (B), stochasticity and systematic biases in evolutionary modes and rates yield trees with partially incorrect topology and often-misplaced roots [1]. Misplaced branches (red) are shown as extra long, but in practice misplaced taxa often do not reveal themselves so neatly. In practice, root positions in paralogue subtrees may both be right (very rare: I recall no examples), both wrong but the same (implying strong systematic biases), both wrong but different (often reflecting stochasticity and poor resolution), or one right and one wrong. When such conflicts occur among different paralogue pairs (or triples, etc.), as is almost invariable, other means are required to decide between them.

Gene duplications can in principle be used to root a subtree like eukaryotes or the whole tree of life. If duplication was just prior to the last common ancestor of a group and all descendants retain both paralogues, data from both can be combined in one tree. In theory, each paralogue would give an identical tree, with both trees linked by a line connecting their roots (Fig. 1a). In practice paralogue rooting is highly problematic; different gene pairs put roots in contradictory places and the two subtrees may not be identical [13] (Fig. 1b). This is because double trees are subject to systematic biases and/or poor resolution like single-gene trees [1]. For many paralogue pairs these problems are worse than most single-gene trees; this arises because most paralogues kept in all descendants of a particular ancestor underwent temporary dramatically elevated rates of change immediately following duplication when their contrasting functions that allowed both to survive originated [1]. For two proteins in the same cell compartment (virtually all in bacteria) this general principle (analogous to ecological limiting similarity dictating species coexistence [14]) makes transiently hyper-fast early divergence between paralogues almost inevitable. Thus sister paralogues are each very long branches on the twin tree [15,16] that evolve with different constraints: the worst combination of properties for accurate phylogenetic construction [1,2]. Any lineage of either or both paralogues that underwent similar major changes in rate or mode will be put artifactually closer to the apparent root than is correct. Interesting possible exceptions, which might give sensible roots, are sister paralogues retaining almost the same functions in separate compartments, e.g. cytosolic and endoplasmic reticulum Hsp90 [17].

I previously highlighted two contrasting classes of universal paralogue tree [1]. Those for metabolic enzymes mostly place the root within eubacteria (in conflicting places with different enzymes [18]) and show weak support for monophyly of archaebacteria, which nest within eubacteria. In sharp contrast, trees for DNA-handling enzymes, molecules associated with ribosomes [15], and a few others, e.g. membrane ATPase [16], typically place the root on a very long stem that separates archaebacteria and eubacteria into unambiguously distinct branches. The latter trees are the minority but have often ('somewhat surprisingly': [2]) been accepted as genuinely locating the root, and the conflicting majority showing eubacterial roots ignored [19,20]. Such neglect of important conflicting evidence and of other approaches that may be more productive stems from the first paralogue trees used for rooting being of the minority type [15,16] and from a perceived fit to long-standing assumptions (devoid of sound evidence) that archaebacteria are as ancient as eubacteria. Instead of ignoring conflicting evidence, we need to understand why the trees differ and which most reliably locates the root. In essence, we are caught between the Scylla of strongly systematically-biased molecules that give the wrong root with high confidence and the Charybdis of less-biased, weakly-resolving molecules that give the right and several wrong versions of the root with too little support to distinguish them [1]; transition analysis, if critically applied, can pilot us into safer waters. Although it may not give the absolute certainty that some crave, it can allow us to reconstruct the past history of life with much higher confidence than anyone would have dreamed of a few decades ago.

### Cladistic analysis of discrete characters can improve the resolution of ambiguous trees

Molecular sequence trees have not established the branching order of the nine eubacterial phyla recognized here (Table 1). Basal resolution of single-gene trees like 16S rRNA is totally inadequate. Multi-gene trees and genomic trees confirm most major clusters indicated by single-gene trees, but lack resolution in most key areas and are still too weakly sampled taxonomically [21-23], with Chlorobacteria still unrepresented (the first to include a chlorobacterium appeared during review of this paper; it is remarkably congruent with the present analysis if properly rooted and is discussed in responses to referee 3). Some evolutionarily key organisms are greatly neglected. A worse problem with multiple-gene trees is genome-wide systematic biases that can give the wrong topology with increasing confidence as data are added [2]. Cladistic reasoning about unique or rare changes has a special role in formulating and testing relationships, having been decisive in eukaryotes, e.g. in creating and strongly corroborating the chromalveolate theory [24-26] and locating the root of the eukaryotic subtree [3,4,6]. The value of such characters depends on their complexity and rareness. Ideally one prefers congruence among several; when congruent they may be sounder than many genome-wide comparisons. This paper uses rare discrete characters to establish unambiguously the branching order among the 10 eubacterial phyla, and to establish the monophyly of Posibacteria, by seeking synapomorphies that group them together in the same way as has been very successful in eukaryotes [3,4,6,27,28].

### Multiple transition analyses of complex multimolecular characters can root the tree

**Figure 2:**
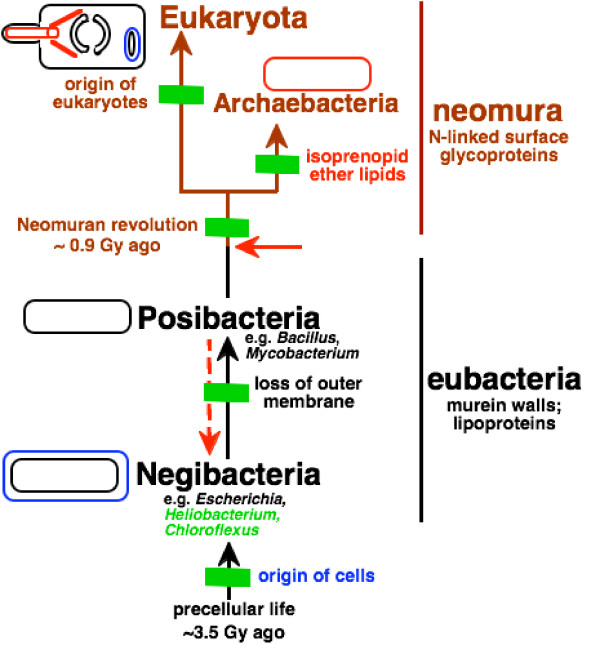
Evolutionary relationships among the four major kinds of cell. The horizontal red arrow indicates the position of the universal root as inferred from the first protein paralogue trees, i.e. between neomura and eubacteria. To determine whether the root is really there or within eubacteria, as suggested instead by many paralogue trees for metabolic enzymes, we must correctly polarize the direction of the negibacteria/posibacteria transition that took place in bacteria that had already evolved flagella. As argued in detail in the text, flagellar evolution and wall/envelope evolution both strongly favour a transition from negibacteria to posibacteria (continuous black arrow), not from posibacteria to negibacteria (broken red arrow). This places the root within Negibacteria and shows that the ancestral cell had two bounding membranes, not just one as traditionally assumed. A negibacterial root also fits the fossil record, which shows that Negibacteria are more than twice as old as eukaryotes [1, 129]. As negibacteria are the only prokaryotes that use sunlight to fix carbon dioxide this is also the only position that would have allowed the first ecosystems to have been based on photosynthesis, without which extensive evolution might have been impossible. Posibacteria, archaebacteria and eukaryotes were probably all ancestrally heterotrophs, whereas negibacteria are likely to have been ancestrally photosynthetic and diversified by evolving all the known types of photosystem and major antenna pigments.

Figure 2 emphasizes that the most fundamental question concerning the root of the tree of life is whether the ancestral cell had two bounding membranes (i.e. was a negibacterium, as argued here) or just one membrane as in archaebacteria and posibacteria (collectively therefore called unibacteria [1]), as has traditionally been widely assumed. To decide this one must correctly polarize the transition between cells that have two membranes (most bacteria, in eight phyla, grouped as Negibacteria in Table 1) and those with only a single surface membrane (eukaryotes and two bacterial phyla: Posibacteria and Archaebacteria); in other words one must decide whether evolution occurred from Negibacteria to Unibacteria or the reverse. Given the topology of the tree, if it can be shown that Posibacteria evolved from Negibacteria, not the reverse, then the root cannot lie between neomura and eubacteria, as widely supposed, but must lie within negibacteria. Thus we can firmly establish the position of the root of the tree by determining (a) its correct topology and (b) the direction of major transitions within it. This paper shows that several major transitions within eubacteria can be unambiguously polarized and that no strongly polarized transitions conflict with each other. I show that all the more robust polarizations are consistent with a negibacterial root but that several of them contradict alternative hypotheses: i.e. that the root is between neomura and eubacteria [15,16], or between posibacteria and negibacteria, or within either neomura [13] or posibacteria. The direction of some transitions is ambiguous, but enough can be polarized sufficiently confidently to exclude all phyla except Chlorobacteria from the root of the tree.

Of the five major transitions shown by green bars in Fig. 2, the prokaryote-eukaryote transition was analyzed in considerable detail before [27,29], as were the eubacterial to neomuran and the neomuran to archaebacterial transitions [1]. The first transition, from non-life to negibacteria, i.e. the origin of the first cell has also been considered in detail [30,31]. Since those papers were published major advances have been made in rooting the eukaryote tree of life [3,4,28], which have important implications for the universal tree. It is now generally accepted that all extant anaerobic eukaryotes had ancestors with aerobic mitochondria [32] and highly probable that the root lies between unikonts and bikonts [3,4,28]. Thus the last common ancestor of eukaryotes was a sexual aerobe with mitochondrion, and probably also a cilium and capacity to make pseudopodia and dormant cysts. The fact that the eukaryote cenancestor had mitochondria, which arose from enslaved α-proteobacteria [33], means that eukaryotes must have evolved long after eubacteria, which must have diversified to produce proteobacteria and α-proteobacteria before the first eukaryote. This raises a severe problem for the common, but seldom critically evaluated, assumption that the root lies between neomura and eubacteria (red arrow Fig. 2); on that widespread assumption [15,16] eukaryotes would have originated in the very first bifurcation on the neomuran side of the tree. Given that hypothetical position of the root and the topology of the tree, the basal eubacterial group would have been posibacteria; negibacteria would probably not have evolved by the time of the primary neomuran bifurcation, whereas proteobacteria and α-proteobacteria would each have arisen much later still. Such a later origin of α-proteobacteria than eukaryotes is now untenable. Bayesian relaxed molecular clock analyses calibrated by multiple palaeontological dates for 143 proteins [34,35] and for 18S rRNA [36] suggest that the eukaryote cenancestor was only 0.9–1.1 Gy old, whereas the fossil record indicates that eubacteria are at least 2.8 and probably about 3.5 Gy old [1,37]. Thus there is now a very strong temporal and evolutionary incompatibility between the now well-established chimaeric and aerobic nature of the oldest eukaryote and the widespread (and, I have argued, false [1]) assumption that neomura are as ancient as eubacteria. There is no fossil evidence whatever that archaebacteria are older than eubacteria – or even as old as them; given the extensive phylogenetic evidence that archaebacteria are sisters of eukaryotes, it is now very hard indeed to escape the conclusion that neomura were derived from eubacteria, not the reverse, and that the universal root lies in eubacteria not between eubacteria and neomura.

Here I use transition analysis arguments that are entirely independent of the fossil record to show that this is indeed the case and that both the tree topology and the root shown on Fig. 2 are correct. I provide the first detailed analysis of the negibacteria to posibacteria transition, which unambiguously polarizes it in that direction, and argue that Posibacteria evolved from the new phylum Eurybacteria, established here (Table 2). I give new evidence for the monophyly of Posibacteria, for the derived nature of Actinobacteria compared with Endobacteria, and a new argument from proteasome evolution that also places the universal root within eubacteria and thus excludes it from the eubacteria/neomura junction. I also analyze 13 transitions within negibacteria (the eight shown on Fig. 2 plus five less important ones within gracilicutes) in sufficient detail unambiguously to root the tree, and map other characters onto the resulting tree. Given this root, sequence trees, cladistic trees, the fossil record, and polarizations deduced by transition analysis are all congruent and thus mutually reinforcing. I also argue that a root within negibacteria is ecologically plausible but any other position is not. One paragraph is first necessary to summarize the conclusions form the previous polarization of the neomuran revolution [1].

**Table 2:** The 10 phyla (=divisions) of the kingdom Bacteria* recognized here

Formal name	Informal names	Examples
Subkingdom Negibacteria* (invariably with acyl-ester phospholipid-containing outer membrane: OM)		
Infrakingdom Glidobacteria* infraking. nov. (Description: gliding motility only; primitively lack flagella, endospores, and haem catalase III. Type order: Nostocales)		
Superdivision Eobacteria* superking. nov. (earlier infrakingdom and division [1]. Description: no lipopolysaccharide or diaminopimelic acid, TolC or TonB)		
Phylum Chlorobacteria	green non-sulphur bacteria	
	(Chloroflexi, Thermomicrobia, GNS group)	*Dehalococcoides*
Phylum Hadobacteria	*Deinococcus*/*Thermus *group	*Thermus*
Superdivision Cyanobacteria* superking. nov. (Description: flagella entirely absent; with lipopolysaccharide, diaminopimelic acid, oxygenic photosynthesis, TolC, TonB).		
Phylum Cyanobacteria	cyanobacteria, blue-green algae	*Nostoc*
		*Synechococcus*
Infrakingdom Eurybacteria* infraking. nov.^1 ^(typically with endospores; external flagella or gliding motility)		
Phylum Eurybacteria* div. nov.^1^	Classes: Selenobacteria* cl. nov.^2^	*Sporomusa*
	incl. Heliobacteriales ord. nov.	*Heliobacterium*
	Fusobacteria cl. nov.^3^	*Leptotrichia*
		*Fusobacterium*
	Togobacteria (Thermotogales)	*Thermotoga*
**Infrakingdom Gracilicutes infraking. nov.**^4 ^(murein sacculus very thin or absent; no endospores)		
Phylum Spirochaetae	spirochaetes and leptospiras (endoflagella)	*Treponema*
Phylum Sphingobacteria (fast gliding; mostly non-flagellate; unique MotB homologue – see text)		
Class Chlorobea	Chlorobi	*Chlorobium*
Class Flavobacteria	CFB group and Fibrobacteres	*Cytophaga*
		
**Superphylum Exoflagellata **(external rotary flagella with both L- and P-rings; no sulfonolipids)		
Phylum Proteobacteria	proteobacteria (flagella; sometimes gliding; always murein)	
Subphylum Rhodobacteria	purple bacteria; α-, β- and γ-proteobacteria	*Escherichia*
		*Rhizobium*, *Spirillum*
Subphylum Thiobacteria	δ- and ε-proteobacteria	*Desulfovibrio*
	(including gliding Myxobacteria)	*Geobacter, Bdellovibrio*
	plus Aquificales	*Helicobacter, Aquifex*
Subphylum Geobacteria	Deferribacteres, Chrysiogenetes and Acidobacteria groups	*Geovibrio*
		*Acidobacterium*
Phylum Planctobacteria^5^	Planctomycetales (flagella; no murein) and Chlamydiae/Verrucomicrobia group	*Pirellula*
		*Chlamydia*
		
Subkingdom Unibacteria* (ancestrally with only a single cell surface membrane; absence of OM with acyl-ester phospholipids and of slime-secretion or pilus-based gliding motility)		
Phylum Posibacteria*	Gram-positive bacteria (ancestrally very thick murein with lipoprotein sortases; both lost only in Mollicutes)	
Subphylum Endobacteria^6^	'low-GC Gram-positives'^6 ^+	*Dictyoglomus*
	i.e. Teichobacteria (murein)	*Bacillus*, *Clostridium*
	Mollicutes (no murein)	*Mycoplasma*
Subphylum Actinobacteria*	high-GC Gram-positives^7^	*Mycobacterium*
		*Streptomyces*
Phylum Archaebacteria	archaebacteria, archaea (isoprenoid ether lipids and N-linked glycoproteins; no murein or lipoprotein)	
Subphylum Euryarchaeota	euryarchaeotes (e.g. methanogens, halophiles)	*Thermoplasma*
Subphylum Crenarchaeota	crenarchaeotes	*Sulfolobus*
		Thermoproteales

Informal names are mostly as used in GenBank. The formal, validly published names are explained in detail in [1] with key defining characters, except for four modifications here, i.e. accepting Eurybacteria [69] as a genuine, but slightly revised, phylum and Selenobacteria as a class (originally phylum [142]), placing both Selenobacteria and Thermotogales in Eurybacteria, and revising the circumscription of Gracilicutes. I use the name Proteobacteria more broadly than usual to include also Geobacteria and Aquificales [1]. All 10 phyla are monophyletic on the backbone bacterial tree , except for Posibacteria and Proteobacteria (the grouping there of typical ε-proteobacteria with Sphingobacteria and exclusion of Geobacteria from Proteobacteria are all arguably artefacts of tree reconstruction related to the almost non-existent resolution at the base of eubacteria on single-gene trees [1]) and the artifactual attraction of the hyperthermophiles *Aquifex *and *Thermotoga *towards the long-branch archaebacterial outgroup. The latter artifactual attraction is not seen on a 31 protein 191 species tree [175] on which all my phyla would be monophyletic if just three branches (the position of none strongly supported) were moved, as discussed in responses to referee 3; that tree even puts Acidobacteria within Proteobacteria (98% bootstrap support), being thus and in other ways much superior to single-gene trees (including rRNA). Note that the early definition of 10 major eubacterial 'taxa' (actually clades, not taxa) based on 16S rRNA signature sequences [201] was remarkably good and durable, better in some respects (e.g. treatment of what was a little later named Posibacteria [29] as a single phylum) than later ideas based on trees [68], which can be misled by such artefacts; all 10 of those early-recognised major clades are represented as high-level taxa in the present system, six as phyla and four as subphyla (within Proteobacteria and Sphingobacteria); the only phylum in this table not foreshadowed by that rRNA signature analysis is Eurybacteria, which if they are indeed paraphyletic would not have exclusive rRNA signatures, unless they were lost during the formation of Posibacteria.

Many, possibly all, of the so-called 16S rRNA 'deep' branches from thermophilic organisms (whether cultured, e.g. *Thermotoga*, *Aquifex*, *Dictyoglomus*, *Thermodesulfobacter*, *Coprothermobacter*, or environmental) that have convergently acquired high GC likely to bias analyses are likely to be phylogenetically misplaced members of one of these 10 phyla rather than genuinely distinct lineages [1, 46]. Bergey's Manual [202] and GenBank use too many small 'phyla', not recognising Sphingobacteria or Planctobacteria even though both are robustly holophyletic on concatenated rRNA trees [18], and Sphingobacteria is strongly supported by indel analysis [83] and recovered by the 31 protein tree [175]; see [1, 69, 142] for a more preferable, organismally oriented, high-level bacterial classification that underlies the simpler system and phylum and subkingdom names used here, and is comprehensive to the class level (but note that the classes suggested for Actinobacteria are probably unsound; they are better retained as a single class [49], pending further research), and in which all names were validly published. *Probably paraphyletic taxa are marked with an asterisk.

^1^Formal description: Eurybacteria ([69] but not yet validated by a listing in IJSEM) phyl. nov. Negibacteria, usually with outer membrane lipopolysaccharide, but lacking the two domain insertions in RNA polymerase that characterise Gracilicutes; flagella often present, with L-rings and P-rings; endospores frequently present. Murein, if present, with cadaverine. If anoxygenic photosynthesis present, with bacteriochlorophyll g and no chlorosomes. Etym. Gk *eury *broad (because of broad range of phenotypes) *bacterion *rod. Type order Heliobacteriales ord. nov. Description: anaerobic flagellate or sometimes gliding photoheterotrophic (non-CO_2 _fixing) endospore-forming negibacteria with a homodimeric photosystem similar to photosystem I and bacteriochlorophyll *g*; type genus *Heliobacterium*. Etym. Gk *helios *sun *bacterion *rod. After type genus.

^2^Formal description: Selenobacteria (Cavalier-Smith 1992 as phylum [142], name based on included genus *Selenomonas*) cl. nov. Often flagellate negibacteria that are endospore formers or have secondarily lost endospores. Type order Sporomusales ord. nov. non-photosynthetic endospore-forming negibacteria and negibacterial descendants without spores. Etym. Derived from the type genus *Sporomusa*. The class also includes Heliobacteriales. (Selenobacteria are inappropriately lumped by many with Endobacteria under the informal name 'low-GC Gram-positives').

^3^Formal description: Fusobacteria (Cavalier-Smith 1998 as phylum [69] cl. nov. Non-spore forming, heterotrophic non-flagellate negibacteria with lipopolysaccharide and Omp85 in outer membrane but lacking the two domain insertions in RNA polymerase that characterise Gracilicutes (Fig. 7 legend); type order Fusobacteriales ord. nov. description as for Fusobacteria, type genus *Fusobacterium*; Etym Gk *fus*- spindle *bacterion *rod, after type genus.

^4 ^Formal description: Gracilicutes (Gibbons and Murray 1978 [203], originally a division that excluded spirochaetes) infraking. nov. Negibacteria in which the peptidoglycan sacculus, if present, is invariably very thin, and with one or both of two distinct inserts in RNA polymerase: αβ-β' module domain 1 inserted into the universally conserved second sandwich barrel hybrid motif domain in the β-subunit; a long helical module in subunit σ; outer membrane with lipopolysaccharide or lipo-oligosaccharide and Omp85. Type order Chlorobiales.

^5 ^Monophyly of Planctobacteria, repeatedly questioned since the relationship between *Planctomyces *and *Chlamydia *and the name were first proposed [30], is now well supported by multiple protein trees [204] as well as by concatenated rRNA trees [18].

^6 ^Unlike in [1] Endobacteria now excludes Selenobacteria and Thermotogales because they are now established as Negibacteria (see text). 'Low GC-Gram positives' in most recent usages include the genetically related, but phenotypically non-Gram-positive, Selenobacteria and Mollicutes; by embracing two major and phenotypically very different non Gram-positive classes it is now descriptively profoundly misleading in this sense; the term 'low GC-Gram positives would be best restricted to Teichobacteria alone or else abandoned. The now frequently synonymous name Firmicutes also is not used here, as it has become thoroughly ambiguous and is probably best forgotten; it was originally invented for Actinobacteria plus Teichobacteria, but is now commonly contradictorily and inappropriately used instead for Teichobacteria, mycoplasmas and Selenobacteria collectively, a probably paraphyletic and phenotypically most heterogeneous assemblage, just because these taxa usually form a single branch on 16S rRNA trees. This usage destroys the whole point of the name, which was to contrast the thick-walled unimembranous Actinobacteria/Teichobacteria with the wall-less unimembranous mycoplasmas (*Molli*- soft; *Firmi*- hard; *cutes *skin in Greek). Confusingly the older usage still occurs, but often inappropriately modified to include Mollicutes. The now most prevalent misuse of Firmicutes comprises one group with one membrane and no wall, one with two membranes and a thin wall, and only one of the two groups that have a single membrane and thick wall; probably this group is not holophyletic, but a pseudoclade arising because actinobacteria are artifactually excluded from it because of their very high rRNA GC composition and/or elevated evolutionary rate and because the dramatic quantum evolution and persisting higher evolutionary rate of neomuran rRNA also artifactually drives them still further away [1]. Applying any name – even were it appropriate – to this artifactual pseudogroup is a taxonomically meaningless prime example of how not to use the potentially very valuable information that 16S rRNA trees can provide; like all other information, 16S RNA trees must by tested by their congruence (or incongruence in this case) with independent lines of evidence.

^7^A few early branching genera of 'high-GC Gram-positives' (e.g. *Symbiobacterium *[51]; Rubrobacterales [205]) are more like Endobacteria in some respects, showing that the original distinction between Endobacteria and Actinobacteria has broken down (see discussion in text). The status of such borderline organisms needs clarifying by a taxonomically broad and critical phylogenetic analysis of many conserved proteins and is potentially very important for understanding the origins of actinobacteria and neomura.

### The neomuran revolution

Morphological fossil evidence that eubacteria are several times older than eukaryotes plus strong phylogenetic evidence that archaebacteria are holophyletic sisters of eukaryotes (together comprising the clade neomura [29]), not their paraphyletic ancestors, strongly indicate that archaebacteria are much younger than eubacteria [1]. Transition analysis showed that 19 major changes in the immediate common ancestor of neomura can all be polarized in the direction from eubacteria to neomura, most by strong selective arguments, none making sense in reverse [1]. These numerous coevolving changes constitute the 'neomuran revolution', the second most important change in cell organization apart from the immediately following origin of eukaryotes [1]. Most of the 19 (now 20) neomuran innovations are explicable as consequences of stronger cotranslational protein secretion associated with the replacement of murein cell walls by cotranslationally-synthesized N-linked glycoproteins (neomura means new walls), or of the simultaneous replacement of eubacterial DNA gyrase by core histones [1]. Both key innovations were arguably adaptations to thermophily [1]. For want of space these very detailed arguments are not repeated or even summarized here; nor shall I repeat my detailed discussion of the fossil record and the weakness of claims from it of an early origin for neomura [1]. The best attempt since then to date the primary divergence of eukaryotes using sequence trees multiply calibrated by the fossil record [34,35] is consistent with my argument that eukaryotes are well over a billion years younger than eubacteria [1]. The recent discovery of histone genes in crenarchaeotes [38] eliminates one line of 'evidence' for claims that archaebacteria are ancestral to eukaryotes rather than their sisters by supporting my contention that histones were already present in the last common ancestor of archaebacteria and of neomura [1]. This considerably strengthens the thesis that the large differences in DNA-handling enzymes of neomura, compared with eubacteria, were caused by rapid coevolutionary adaptation to the origin of histones in the neomuran cenancestor [1].

## Methods

The main methods used were transition analysis and congruence testing as outlined above. BLAST and examination of resulting alignments and domain identifications by CDD was frequently used to check homology among potentially related sequences and to extend the literature information about the distribution of key characters across phyla. All BLAST results mentioned were by simple P-BLAST, except for those for Omp85, which additionally used PSI-BLAST in an unsuccessful attempt to detect more divergent homologues in Chlorobacteria. In many cases I used several phylogenetically divergent queries and also reciprocal BLASTs of hits that were rather low; in some cases reciprocal BLAST was dramatically better at picking up strong relationships. BLAST hits with E values above 10 were considered to lack detectable homology.

## Results and discussion

**Figure 3:**
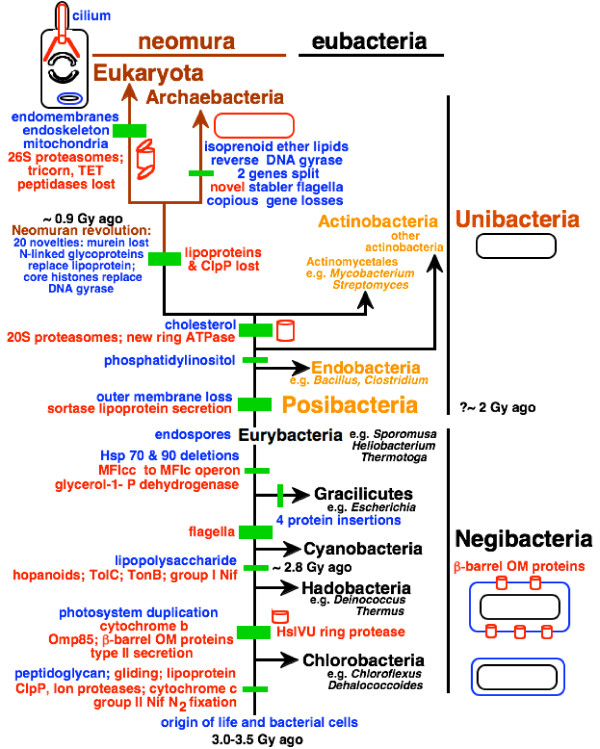
Key molecular cladistic characters that help root the tree of life. Green bars mark major evolutionary innovations. Those explained in detail in previous publications [1, 24, 26] are labelled in blue. Those introduced for the first time or discussed in more detail in the present paper are in red. The three most fundamental changes in cell structure (the origin of unibacteria by loss of the negibacterial outer membrane [1, 5]; the neomuran revolution involving novel chromatin and glycoprotein secretion and much coadaptive macromolecular evolution [1, 5, 29, 62]; and the origin of the eukaryote cell [5, 27, 62]) are marked by thicker bars. So also are the three major transitions, whose key importance and decisiveness for rooting the tree of life are explained here for the first time: the origins of the proteasome, of flagella, and of Omp85 for insertion of OM β-barrel proteins. The three major kinds of cell from the viewpoint of their having fundamentally distinct membrane topology (eukaryotes, unibacteria, negibacteria) [5, 29, 56, 62] are shown by thumbnail sketches (isoprenoid ether lipids in red, outer membranes in blue). Thumbnail sketches also illustrate the inferred times of origin of two key cylindrical macromolecular assemblies (the OM β-barrel protein Omp85 and HslVU/proteasome ATP-dependent regulated proteases) and the two-step increased complexity of the latter. Negibacterial taxa are shown in black, Posibacteria in orange, and neomuran taxa in brown. Gracilicutes comprise four negibacterial phyla with either a very thin peptidoglycan layer or no peptidoglycan at all in their cell envelope: Proteobacteria, Planctobacteria, Spirochaetae, Sphingobacteria (Table 1 explains the formal bacterial taxon names used here for precision and brevity). Evidence for the relatively late dating of the neomuran revolution was explained in detail previously [1]. Note that although Chlorobacteria and Endobacteria are shown as holophyletic, either or both might actually be paraphyletic; I suspect that Endobacteria may be paraphyletic as the most divergent actinobacterium has endospores, but think that Chlorobacteria are probably not. Conversely, it is uncertain whether actinobacteria are paraphyletic as shown or paraphyletic; see text – further work is needed to decide. For simplicity, five additional polarizations within Gracilicutes that are also discussed are not shown; see the more comprehensive Fig. 7 for them and additional characters mapped onto the tree. Note that the ~2.8 Gy date for the origin of cyanobacteria is based solely on hopanoid biomarkers; since no earlier organic deposits have been found that are sufficiently well preserved and with enough extractable hydrocarbons for such biomarker analysis, this is a minimum date (though its validity also depends on the assumption that such hydrocarbons have not migrated vertically in the rocks since being formed, which is hard to test).

To orient the reader in the following complex discussion, Fig. 3 indicates the 12 major transitions that will be discussed; five lesser transitions within Gracilicutes are also considered, making 17 in all (13 within eubacteria). I shall start with the evidence that actinobacteria are sisters of or ancestral to neomura, then work systematically down the tree to the root, discussing each transition in turn, and finally discuss overall implications of this new rooting. As Fig. 3 indicates, a major new line of evidence for polarizing the upper part of the tree concerns stepwise increases in complexity of the HslV and proteasomal proteases, both of which are absent from Chlorobacteria. Before explaining the logic, I provide a little background information about controlled proteolysis within hollow cylindrical macromolecular assemblies, which is essential for all life. I have attempted to present the following discussion in sufficient detail for specialists to check and criticize the validity of all the major points, but have shorn away as much detail as possible to expose the fundamental evolutionary points and to attempt to make the argument reasonably accessible to a broad audience. It is an analysis and synthesis, not a comprehensive review.

### Intra-cylinder ATP-dependent proteolysis (protein digestion)

Three different families of ring-shaped or cylindrical macromolecular assemblies have evolved to allow controlled ATP-dependent proteolysis in cells [39]. I shall argue that two of them, ClpP protease and Lon protease [40] had evolved prior to the bacterial cenancestor, whereas HslVU [39] evolved only after the divergence of Chlorobacteria and higher organisms. In all three cases the proteolytic site is inside a hollow cylinder, in its central part as far away from entry channels as possible, which maximally protects external proteins from digestion unless they are actively pulled inside with the help of an associated ATP-dependent chaperone that recognizes only the correct proteins for destruction. In the Lon protease the chaperone and ATPase activities are part of a single large tripartite multifunctional polypeptide chain that is capable of self-assembly. Its N-terminal region is important for this assembly; its middle part has the ATPase/chaperone function; and its C-terminus has the protease activity. The protease and ATPase moieties each independently assemble into hexameric rings and it is thought that they then form a two-tiered hexamer with the digestive site on the inside. By contrast, in ClpP and HslVU/proteasomes, the chaperones and proteases are distinct and much smaller polypeptides coded by evolutionarily unrelated genes (but are confusingly given similar names despite this). Each assembles as a hollow ring and the whole assembly is formed by an ATPase ring sticking to each end of the protease ring/cylinder, in a suitable position to monitor substrate entry.

Lon is present right across the living world but not found in every species [40]; soluble LonA proteases are eubacterial or mitochondrial, whilst archaebacteria only have membrane-bound ones (LonB) with an extra membrane-spanning domain inserted within the ATPase domain. ClpP is present throughout eubacteria and in all chloroplasts, but not in any eukaryotes without plastids; this suggests that it was lost prior to the origin of eukaryotes but regained by photosynthetic eukaryotes when a cyanobacterium was enslaved to make chloroplasts. It is also absent from all archaebacteria except *Pyrobaculum *and *Methanosarcina*; as the latter is known to have acquired vast numbers of genes from eubacteria by lateral transfer [41] it is probable that ClpP was lost in the neomuran not the eukaryote ancestor (Fig. 3), and that both archaebacteria reacquired it by lateral transfer; proper phylogenetic analysis is needed to test this.

**Figure 4:**
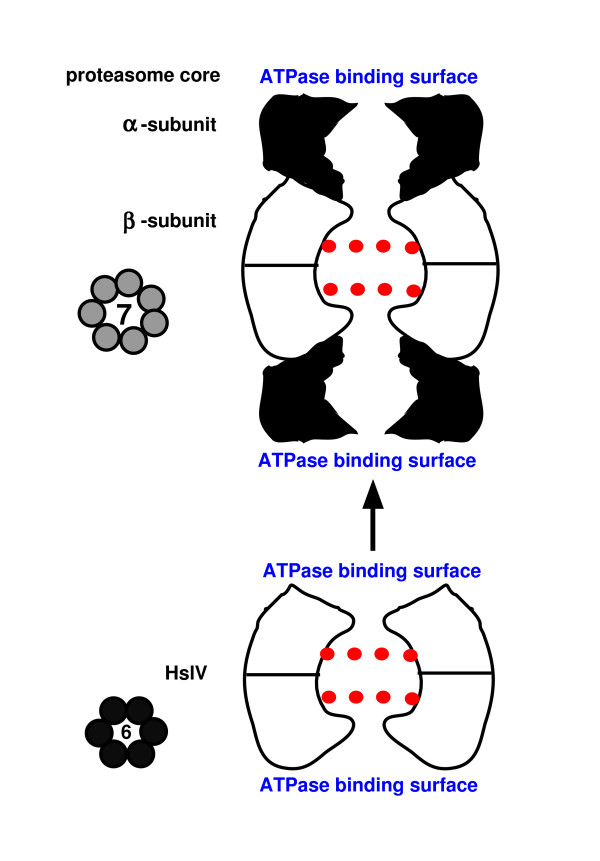
Schematic longitudinal sections through the two-tier HslV and the four-tier bacterial 20S proteasome core particle. Red dots are proteolytic active centres. Thumbnail sketches on the left of the main figure are cross sections through the proteolytic chamber showing respectively their 6-fold and 7-fold symmetry. Evolution from the 12-mer HslV to the 28-mer proteasome by duplication to form α- and β-subunits forming heptameric rings is shown by the arrow; loss of proteolytic activity by the new α-subunit (black) coupled with a new ability to stack onto the β-subunits would have expanded the digestive cavity radially and longitudinally and kept potentially vulnerable external proteins further away from the proteolytic centres. Changed dimensions and shape of the α-subunit's ATPase binding surface probably favoured replacement of the HslU ATPase ring by a different one. Hypothetical evolution in the reverse direction by loss of the α-subunit's would have created a less efficient purely β-subunit 14-mer that might have lost any ability to bind an ATPase ring through adapting to α-subunit binding instead and with a broader digestive cavity and entry pore more likely to digest the wrong proteins. It is unlikely that it could have survived purifying selection long enough to reduce its symmetry to sixfold and find a new ATPase partner to bind and thus generate HslVU. No selective advantage for simplification of a proteasome to HslV is apparent. Subunit shapes simplified from [199].

ClpP protease is a ring with 7-fold symmetry [40], whereas its unrelated chaperone ATPase ring (ClpX or A [42]) has 6-fold symmetry, being made from six monomers. HslV protease has six subunits (Fig. 4), as does its unrelated chaperone ring HslU, which allosterically activates it [43]. The proteolytic cylinder has 7-fold symmetry and its unrelated ATPase chaperone 6-fold symmetry. However, sequence analysis indicates a rather complex pattern of relationships. Although ClpP, HslV, and proteasomal proteases are all very distantly related, ClpP serine protease belongs to a different superfamily (acyl-CoA decarboxylase/isomerase) from proteasome α- and β-subunits and HslV (threonine NTN hydrolases) [19] and cannot therefore be their ancestor. Thus the heptameric proteasomal protease is much more closely related to the hexameric HslV, not to the ClpP protease, which has a fundamentally different tertiary protein-folding pattern. The ClpX and HslU chaperones are closely related members of the AAA+ ATPase superfamily; thus they probably either had a common ancestor or one evolved from the other. The proteasomal chaperones are also AAA+ ATPases but belong to a different family, being related to the ATPase domain of the 3-domain membrane inserted protease FtsH of eubacteria and chloroplasts; in fact the ATPase component of all ATP-dependent proteases including Lon and FtsH are AAA+ ATPases that assemble as hexamers, like other still more distant members of that family.

**Figure 5:**
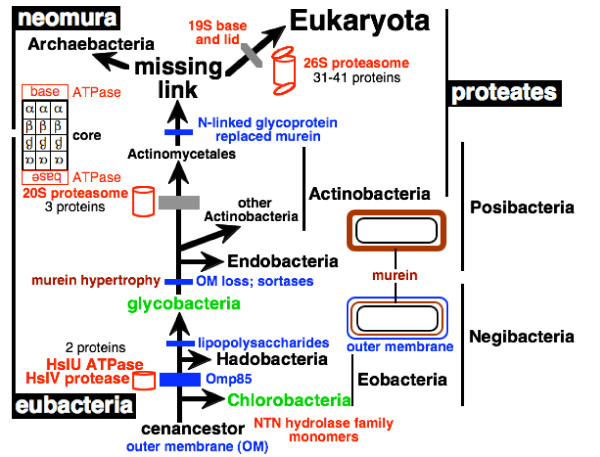
Proteasome evolution showing step-wise increase in complexity, first to the HslV ring protease, then to the 20S proteasome, and lastly to the 26S proteasome; the two major transitions in proteasome structure important for polarizing the tree are marked by grey bars. Blue bars mark four other important evolutionary transitions that also congruently polarize the tree. HslV has 6-fold symmetry (a 2-tiered ring of 12 identical subunits) and arose from a monomeric NTN hydrolase, probably just before Hadobacteria diverged. HslV rings interact with an unrelated chaperone ATPase, HslU, also having 6-fold ring symmetry, like ClpX chaperone from which it arguably evolved and virtually all AAA+ ATPase proteins, which originated in a burst of gene duplications prior to the last common ancestor of all life [19]. The 4-tier proteolytic core of the 6-tiered 20S proteasome evolved in a common ancestor of neomura and Actinomycetales (jointly proteates) of the subphylum Actinobacteria by another gene duplication that generated its catalytic β- and non-catalytic α-subunits from HslV, with an associated symmetry change to 7-fold: all four rings forming the core of the proteasomal cylinder have 7 subunits, but the 6-fold-symmetric HslU was replaced by another hexameric ATPase ring from a different AAA+ family to make the proteasome 'base' (red in the two-colour sketch of the archaebacterial proteasome at the top left). Glycobacteria [1] comprise all the typical negibacteria with OM lipopolysaccharide, i.e. all negibacterial phyla listed in Table 2 except Hadobacteria and Chlorobacteria).

Proteasomes are hollow cylindrical organelles for intracellular digestion of denatured proteins, found in neomura and advanced actinobacteria (Actinomycetales) only. They have a 15 nm long hollow cylindrical core, the 20S proteasome, with internal proteolytic activity: additional ATP-dependent chaperone structures at either end feed denatured proteins into it for digestion. In all proteasomes the central core has 7-fold rotational symmetry and four tiers of seven protein subunits (Fig. 4). In actinobacteria and archaebacteria the central core has only two kinds of protein: two inner tiers of identical proteolytic β-subunits (threonine proteases) and outer ones of the evolutionarily related non-proteolytic α-subunits (Fig. 4). This notable differentiation in function of the α- and β-subunits and associated change in their symmetry during the evolution of the threonine NTN protein hydrolases is the crux of my argument in the next section for polarizing the direction of evolution. In eukaryotes the core is far more complex, each protease subunit being different; this complication arose by repeated gene duplication during the origin of eukaryotes. In eukaryotes, each end is capped by a complex 'base' of several different proteins, including 6 different, but related, AAA+ ATPase chaperones, and a multiprotein lid open at one side to allow denatured proteins entry [44]. Adding 'base' and lid created a functional 26S proteasome of 31–41 different proteins (Fig. 5) [45]. Actinobacterial and archaebacterial proteasomes are much simpler: ends are terminated by a ring of six identical, but directly related, chaperone AAA+ ATPase proteins, so bacterial proteasomes are built of only three different proteins of two evolutionary groups.

### HslV to proteasome differentiation polarizes the evolutionary transition

I argue here that the proteasome 20S core particle evolved from the simpler HslV, not the reverse. If this evolutionary polarization is correct, it excludes the root of the universal tree from a clade comprising neomura and actinomycete actinobacteria (Fig. 5), the only organisms that have the shared derived character of a proteasome with distinct but evolutionarily related α- and β-subunits, only one of which is enzymatically active. My argument does not depend on the sometimes-controversial fossil evidence [1] or on archaebacteria being holophyletic not paraphyletic [1]. My analysis, if correct, establishes the universal root within eubacteria, in agreement with paralogue trees for metabolic enzymes [1], confirming that archaebacteria are highly derived, not a primary domain of life, and that long-standing interpretations of early life assuming a molecular clock for rRNA have been grossly misleading [1,46].

As explained above, HslV is a single protein, evolutionarily related to both the α- and the β-subunits of the proteasomal digestive core. Twelve HslV molecules are arranged as two tiers of six identical subunits. In its active form it has an HslU ATPase ring at each end. Thus the 24-molecule HslVU protease is markedly simpler than, yet partially evolutionarily related to, the actinomycete/archaebacterial proteasome. The simplest interpretation of the evolutionary origin of proteasomes is that the core proteasome originated from HslV protease by a gene duplication that made functionally distinct α- and α-subunits arranged as a four-tier core rather than as a two-tier core as in HslVU. This increased the length of the protective cylinder and the associated increase in the number of subunits per ring to seven increased the diameter of its hollow lumen, thus expanding the proteolytic chamber in both directions. These concerted changes thus increased the capacity of the proteasome to digest larger proteins and to protect cytosolic proteins from accidental digestion compared with the simpler and smaller HslVU.

I suggest that the increased diameter of the core caused problems with its previous association with HslU, so that this was replaced by a larger more distantly related AAA+ ATPase ring to form the cap attached to each end of the 20S proteasome. In archaebacteria the cap ring is a hexameric ATPase [47] that is related to the ATPase domain only of FtsH protease; its homologue in actinomycetes is a similar hexamer also of identical protein subunits, but interaction with the 20S core has not yet been directly demonstrated [48]. FtsH is very conserved and found in all eubacteria, including actinobacteria where it coexists with the putative cap ring; thus unlike HslVU it did not disappear when proteasomes evolved by being directly converted into the cap ring. Instead gene duplication and with one copy only losing its N-terminal membrane-insertion domain and C-terminal protease domains was probably involved. However, neomura have partially related proteins with two separate ATPase domains, which in eukaryotes form a hexameric ATPase (Cdc48) responsible for chaperoning proteins out of the ER lumen for degradation. Cdc48 seems more closely related to the proteasome cap, which in the ancestral eukaryote became differentiated into a heteromeric structure by gene duplication and divergence, than it is to FtsH, which was probably lost in the ancestral neomuran. Since related two domain ATPases are also found in a sprinkling of Posibacteria and even a few negibacteria higher in the tree (apparently not in Chlorobacteria), such proteins (rather than FtsH) might have been ancestral to the proteasomal ring protease; phylogenetic analysis of each domain is needed to establish the precise evolutionary relationships among them. The main point for this paper is that the ATPase regulatory cap of the proteasome originated from a different AAA+ ATPase from HslU and its origin was a complex process involving gene duplication, domain deletion, and the origin of a novel ability to bind the newly arisen α-subunits of the 20S proteasome. It was not a simple process of molecular transformation with retention of all main functions. Moreover, as for the proteasomal proteolytic core, there was a further increase in regulatory ATPase complexity, involving even more extensive gene duplication, to make the eukaryote 26S proteasome.

An important point is that the α- and β-subunits of the proteasome appear to have diverged from HslV in opposite but mutually complementary directions. Four functions present simultaneously in HslV are partitioned between them. The β-subunits retained the threonine proteolytic active centre at the N-terminus and the capacity to assemble into a homomeric two-tier ring of 12 subunits. But they lost the distally constricted inner rim that narrows the ends of HslV to prevent entry of unfolded proteins into the proteolytic cavity (Fig. 4) and the capacity to bind to the regulatory ATPase ring. At the same time they acquired a new ability to bind the β-subunit ring by the same region of the molecule that lost the distal constriction. It is very likely that these two changes came about by a concerted remodelling of this region of the polypeptide chain. By contrast, the α-subunits lost the proteolytic centre and ability to form two-tier homomeric rings, but retained the distal constriction and the ability to bind an ATPase ring, albeit a different one as argued above. Thus it was the opposite end of the molecule, away from the ATPase-binding site, that was mainly modified in β-subunits.

It is well known that evolution can involve simplification as well as stepwise increases in complexity. Therefore, the fact that one can see functional advantages in the proposed increase of complexity from smaller and simpler HslV to larger and more complex 20S proteasomes, though adaptively much more plausible than evolution in the reverse direction, for which no selective advantage is apparent, is not in itself proof that evolution occurred in that direction. How can we rule out the alternative theoretical possibility of evolution in the reverse direction from the 20S proteasome to HslV by simplification? The clinching argument concerns the differentiation in function between the proteasome α- and β-subunits.

Though logically possible, direct reversal is mechanistically and evolutionarily highly unlikely. It would entail the loss of the non-catalytic α-subunits that serve as a toroidal adaptor for binding the two-tiered proteolytic β-subunit rings to the terminal ATPase rings. Such loss would generate an intermediate two-tiered β-subunit 14-mer without a narrowly constricted protein entry channel or any ability to bind regulatory ATPase rings. Thus it would be very harmful by digesting proteins that it should not and would be strongly selected against. Three major changes would be needed to convert such a defective β-subunit into HslV. The probability that it could simultaneously change its symmetry from 7-fold to six-fold, evolve a narrow entry channel, and evolve an ability to bind an ATPase ring in the short time before the mutant strain was rapidly eliminated by such adverse selection is negligible. Thus simple reversal would in practice be evolutionarily impossible. The fact that mutant *Thermoplasma*s without proteasomes can survive, unless subjected to heat shock, does not contradict this argument. Nor does the fact that proteasomes appear to have been lost by a few actinomycetes that are endoparasites of animals. Simple loss of an entire structure has been observed repeatedly in evolution, but reversal of evolution of a complex highly differentiated structure to form a more generalized and simpler one closely mimicking an ancestral state has, as far as I am aware never been clearly documented. Thus evolution of HslVU from 20S proteasomes is so improbable that we can safely polarize the actual evolutionary change in the opposite direction.

The transition from bacterial proteasomes to eukaryotic 26S proteasomes involved even more complex changes and differentiation among the different subunits, so it could not have occurred in the opposite direction either. The actinobacterial/archaebacterial proteasome is undoubtedly ancestral to the eukaryotic one, not the reverse. The far greater complexity of 26S proteasomes is associated with the origin of ubiquitin, unknown in bacteria but present in all eukaryotes as the most conserved protein of all. Ubiquitin is covalently attached to proteins to target them for destruction by 26S proteasomes; the lid includes proteins helping to recognize the polyubiquitin tags, remove them and push the target protein into the proteasomal digestive lumen. Clearly the extra complexity of base and lid coevolved with the origin of ubiquitin tagging. The greater heterogeneity of the eukaryote proteasome core reflects the greater diversity of substrates that need digesting compared with bacteria.

Arguing that HslVU evolved from proteasomes would leave totally unanswered how 20S proteasomes evolved. If HslV were not the ancestor of the α- and β-subunits, what is? There are no other candidates. Polarizing the tree in the direction shown in Figs 4, 5 explains the origin of proteasomes from HslV in a gradual way that is mechanistically and evolutionarily plausible. Polarizing it in the opposite direction totally fails to explain the origin of proteasomes and postulates changes that are mechanistically and selectively unreasonable, and is thus doubly defective scientifically. Thus mechanistic, selective, and phylogenetic arguments all unambiguously polarize the direction of evolution from HslVU to the more complex 20S proteasome with larger digestive cavity and more strongly bound ATPase caps, not the reverse. This important evolutionary step took place prior to the last common ancestor of all Actinomycetales, as proteasomes are found in all free-living actinomycete genomes so far sequenced, spread right across the 16S rRNA tree [49] and are absent only in a few parasites, almost certainly secondary losses such as are widespread in parasites – perhaps allowed by their greater degree of buffering from environmental heat shocks inside animal bodies. It could have taken place at any time between then and the origin of actinobacteria themselves, about twice as early, judging from 16S rRNA trees [50]. The exact timing is uncertain as genomes of earlier diverging actinobacteria (*Bifidobacterium*, *Symbiobacterium *[51]) lack both proteasome and HslV genes. Presumably one or other was present in their common ancestor shared with Actinomycetales, and has been lost since they diverged. Since, as discussed below, there have probably been many losses of HslVU within eubacteria, but proteasome loss has never been clearly demonstrated among free-living bacteria, losses of HslV seem more likely. If proteasomes have never been lost from free-living bacteria, they evolved only in the immediate common ancestor of Actinomycetales, and thus may be only half as old as actinobacteria. If that is correct and proteasomes have always been vertically inherited, neomura must be more closely related to Actinomycetales (as several other characters such as cholesterol biosynthesis also suggested [1]), making Actinobacteria paraphyletic. However, these parsimony arguments are not decisive evidence for actinobacterial paraphyly. We need more data on early diverging actinobacteria; finding either HslV or proteasomes among them would clarify this. The glycosyltransferases discussed below that support a posibacterial ancestry for neomuran N-linked oligosaccharide biosynthesis are found in Lactobacillales (Endobacteria) but not Actinomycetales; it is thus likely that characters relevant to neomuran origins have been differentially lost in different posibacterial lineages since the origin of neomura from a posibacterium. The important point is that multiple lines of evidence show that either actinomycetes or endobacteria are their nearest eubacterial relatives.

This argument polarizing the evolutionary direction from HslV to the 20S actinomycete core proteasome, not the reverse, uses paralogue rooting – but in a novel way not suffering from the usual tree-reconstruction artifacts: it stresses not sequence trees but two successive increases in complexity of quaternary protein structure – from monomeric NTN hydrolases to hexameric HslV to 14-meric core proteasomes with sharply differentiated functions and 3D structures for the α- and β-subunits. This polarization provides strong evidence that actinomycetes and neomura together form a clade, which I designate 'proteates' because the proteasome with core 7-fold symmetry is its synapomorphy, and thus excludes the root of the tree of life from anywhere within proteates. One can hardly suppose that the complex proteasome core was the ancestral state for all life and that monomeric NTN hydrolases ultimately evolved from it via HslV and two progressive simplifications involving a change of chaperone partner and then its loss. Yet the 'standard model' of bacterial evolution assuming a root between archaebacteria and eubacteria must assume just that and specifically put it between archaebacteria and actinomycetes (to explain their sharing proteasomes). Proteasome evolution excludes the root from proteates (neomura plus actinomycetes) but does not positively locate it. To do this we must polarise several other evolutionary transitions (Fig. 3), as explained below.

The red herring of lateral gene transfer might be raised against the above interpretation. Gille et al. [52] assumed that proteasome genes were laterally transferred from archaebacteria to the common ancestor of actinomycetes. However, they presented no phylogenetic analysis to support this assumption; unpublished trees give no support for lateral transfer, but as the α- and β-subunits and HslV proteins are very divergent and with too long branches for satisfactory phylogenetic analysis, such a possibility cannot be excluded with total confidence (J. Archibald pers. comm.). However there is no positive reason to invoke the total replacement of HslVU by three foreign genes; possibly Gille et al. did so through being unaware of the evidence of a vertical relationship between actinobacteria and neomura and the likelihood that actinobacteria are much older than archaebacteria [1], making the assumed lateral transfer temporally impossible if it is assumed into their cenancestor (though possibly more likely if it were into the ancestor of Actinomycetales alone). Furthermore, assuming lateral transfer from archaebacteria leaves the origin of archaebacterial proteasomes themselves totally unexplained, and ignores the undoubted homology between HslV and proteasomal subunits, and is thus untenable for three independent reasons. Given this homology, there had to be a transition between HslV and proteasomes at some stage.

HslVU is found in Endobacteria (i.e. low-GC Gram-positives plus mycoplasmas, spiroplasmas: see Table 2) and four phyla of Negibacteria [52,53]: proteobacteria, spirochaetes, Sphingobacteria, and many Eurybacteria (e.g. Heliobacteria, *Thermotoga*, but absent from Fusobacteria, which presumably lost it). HslVU is absent from the two entirely non-flagellate bacterial phyla (Chlorobacteria, Cyanobacteria) that are among the best candidates for early diverging life. But this absence is not itself a strong argument for considering them to be primitive, for it is likely that HslVU can be lost evolutionarily. If, as Figs 3 and 5 suggest, HslVU evolved prior to the origin of Hadobacteria, it must have been lost by Cyanobacteria. Its absence from Clostridiales and mycoplasmas suggests loss within Endobacteria. HslVU is currently unknown in Planctobacteria, which for reasons discussed below are unlikely to be at the base of the tree, and thus may have been lost by them. HslV is also absent from Hadobacteria except for *Thermus*, but as its HslV has a highest BLAST hit to *Thermotoga *and an HslU with highest hit to *Aquifex*, it might have been a thermophilically adaptive lateral acquisition from these unrelated hyperthermophiles. If that were true, cyanobacteria and *Deinococcus *need not have lost it, as HslV may have originated after Hadobacteria and Cyanobacteria arose, not just before Hadobacteria as shown on Fig. 3.

Interestingly, trypanosomatid protozoa and Apicomplexa retained proteobacterial HslVU in their mitochondria as well as proteasomes in the cytosol and nucleus – the only known organisms with both [52,53]. The fact that no bacteria are known to harbour both HslV and proteasomes is consistent with HslV having evolved directly into proteasomes.

Given the position of the root of the tree deduced from Omp85 evolution, as explained below, the earliest diverging phylum, Chlorobacteria, lacks HslVU. It is therefore likely that they never possessed it and that it evolved in the last common ancestor of all other bacteria, as shown on Figs 3 and 5. The absence of HslVU from Chlorobacteria, though probably the primitive state consistent with the rooting shown, is – I stress – *not *the primary reason for that rooting, merely a very minor corroboration, given the likelihood that HslVU was lost several times within negibacteria.

In sum, there were three successive increases in complexity: first from an ancestral monomer threonine protease to hexameric HslV, thus increasing the proteolytic repertoire of the common ancestor of eubacteria other than chlorobacteria; then to a 14-mer of two proteins in the actinomycete/archaebacterial 20S core proteasome with an expanded digestive cavity and differentiated function of its α- and β-subunits; thirdly to the markedly more internally differentiated eukaryotic 26S proteasome with expanded proteolytic scope and selectivity. The two latter compellingly polarize the tree of life from non-proteates to proteates and from unibacteria to eukaryotes respectively (Figs 3, 5), and therefore place its root within or among the other eubacterial groups.

Before explaining why the root must be within Negibacteria, I will briefly map onto the tree three main peptidases that further digest the peptide products of the cylindrical ATP-dependent proteases: tricorn peptidases [54], tetrahedral (TET) peptidases [55], and TPP proteases [55]. All are multimeric with a central digestive cavity, but each with unique structures dissimilar from the cylindrical enzymes discussed above. Tricorn peptidases are the most phylogenetically widespread; they were probably present in the prokaryote cenancestor but lost by the ancestral eukaryote at the origin of the 26S proteasome. TET peptidases were probably also lost then and occur only in prokaryotes, mostly those apparently lacking tricorn – for which they may substitute. The statement that TET is more widespread than tricorn [55] seems mistaken, but I agree that tricorn is more ancient. As tricorn needs protein cofactors but TET does not, TET could be acquired by lateral transfer and substitute for tricorn more easily than the reverse; phylogenetic analysis is needed to see if its scattered distribution arose thus, and not by differential loss. Tricorn is a complex two-domain protein with both domains present from *Chloroflexus *(Chlorobacteria) to archaebacteria. BLAST reveals an additional stand-alone paralogue of the C-terminal proteolytic domain only in taxa ranging from Cyanobacteria to Endobacteria; this appears to be absent from Actinobacteria and archaebacteria and perhaps was lost when 20S proteasomes evolved. TPP peptidases are large proteins, like tricorn, but restricted to eukaryotes. BLAST indicates that their proteolytic domain is homologous to the much smaller subtilisin proteases of endobacteria and some negibacteria; the stronger hits to endobacteria fit the topology of Fig. 3; TPP could have evolved from a smaller posibacterial protease by adding a domain.

### Membranome evolution: from negibacteria to posibacteria

For understanding cell evolution we must consider not only genomes but also evolution of the membranome: the set of different genetic membranes that make the cohering supramolecular framework for cell structure [56]. Bacteria fall into two very distinct subkingdoms with respect to cell envelope structure: Negibacteria, all with a double envelope with an outer membrane lying outside the cytoplasmic membrane, and Unibacteria in which the cytoplasmic membrane is typically the only membrane. Proteins of the cytoplasmic membrane are always bundles of α-helices and are inserted directly into it by the SecYE translocon. In most negibacteria outer membrane proteins (Omps) are never α-helix bundles, but almost always β-barrels, some of which form large hydrophilic pores in it, e.g. porins; Omps are translocated across the cytoplasmic membrane by SecYE and then insert specifically into the outer membrane. Of the 10 bacterial phyla (Table 1) only two (Archaebacteria, Posibacteria) are Unibacteria: the rest, which include the majority of bacteria, are all Negibacteria [1].

The most fundamental question about the origin of the first cell [30] is did it have just one membrane, like most Posibacteria, as usually assumed, or two surface membranes like all negibacteria (most bacteria) as Blobel [57] and I [30,37] argued. The negibacterial double envelope is so complex that it must have arisen only once. I previously argued that the origin of the first cell is easier to understand in simple selectively advantageous stages if it was a negibacterium with two membranes [31]; that obcell theory simultaneously explained the origin of the genetic code, the first cell, and the negibacterial outer membrane. That detailed transition analysis from precellular life to the first cell will not be discussed again here, but the origin of the posibacterial cell wall can now be better understood than before because of advances in understanding its biogenesis and also in the wall and membrane structure of the Selenobacteria (see Table 2), which I shall argue are probably ancestral to all Posibacteria.

**Figure 6:**
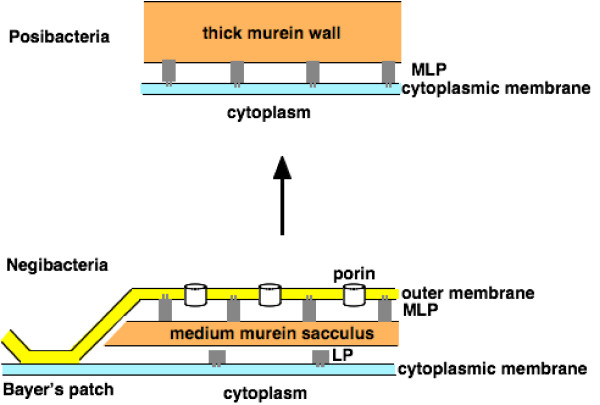
Contrasting cell envelope structure in posibacteria and negibacteria. OM phospholipids, and when present possibly also lipopolysaccharides (LPS), may pass from their site of synthesis in the cytoplasmic membrane to the OM at the Bayer's patch contact sites, but this is not proven and only one protein (Imp) needed for LPS export is yet known. During its biosynthesis murein is secreted across the cytoplasmic membrane by isoprenol carriers. Lipoprotein (LP) is cotranslationally synthesised in both groups. Conversion of a negibacterial wall to a posibacterial wall as shown would be very much simpler than the reverse, requiring only a mutation causing sudden murein hypertrophy that could have broken the OM away from the Bayer's patches, preventing further lipid transfer and OM regrowth, plus the origin of sortases with a novel recognition system for covalently attaching murein lipoproteins (MLP) to the wall. As the negibacteria most closely related to Posibacteria (Eurybacteria) are glycobacteria with much more complex OM, secretion, and import mechanisms than Chlorobacteria (which lack lipopolysaccharide, most porins, Omp85, type I, II, and III secretion machinery, and probably the LolDE lipoprotein release mechanism, of more advanced bacteria), evolution in the reverse direction of such a complex OM in one step from a posibacteria would be practically impossible (see text) and immensely more difficult than the stepwise increase in its complexity possible with a chlorobacterial root of the tree. As the transitional stage between negibacteria and posibacteria had flagella, adding an outer membrane to a posibacterium and evolving a lipid export mechanism in one step would be even more complicated and improbable, as flagellar biogenesis would have had to be conserved and modified at the same time (see Fig. 8). No satisfactory mechanistic explanation has ever been given of how it could possibly have occurred.

Fig. 6 contrasts cell envelope structure in posibacteria and negibacteria. In Posibacteria, except for the almost entirely parasitic Mollicutes (mycoplasmas and spiroplasmas, which lost murein walls) the murein peptidoglycan layers are very thick and are attached to the cytoplasmic membrane by covalently attached lipoproteins with their lipid tails embedded in the outer leaflet of the phospholipid bilayer. In negibacteria the murein is usually much thinner and attached instead to the outer membrane (OM) by covalently attached murein lipoproteins with their lipid tails embedded in the inner phospholipid leaflet of the OM lipid bilayer; unlike in mycoplasmas, lipoproteins are retained in negibacteria even when murein is lost (most Planctobacteria). In Chlorobacteria and Hadobacteria the outer leaflet of the OM bilayer is also simple phospholipid, but in all six other phyla it is lipopolysaccharide (within Spirochaetes a greatly modified version is present in *Leptospira *[58], whereas the obligately parasitic spirochaetes have totally lost it; a few proteobacteria have simplified it to lipooligosaccharide). Unlike the cytoplasmic membrane, the OM is pierced by hollow cylindrical β-barrel porin proteins that allow small molecules to diffuse freely across it [59]. At intervals the OM is in direct and strong adhesive contact with the inner membrane at points known as Bayer's patches where there is a hole in the thin murein wall. As OM proteins and lipids are all synthesized by enzymes associated with the inner, cytoplasmic membrane, they have to be transported to the OM secondarily, through the periplasm for proteins [59] and probably via the Bayer's patches for lipids. Posibacteria entirely lack both the OM and this transport machinery. The OM and Bayer's patch structure can have evolved only once in prokaryote history as its structural and biogenetic complexity is so great. Transition analysis asks would it have been easier for a negibacterium to have lost the OM (evolution from bottom to top in Fig. 6) and make its wall thicker or for a posibacterium simultaneously to add an OM to a cell without them and simultaneously make the wall thinner and invent machinery for export of both lipids and proteins to it and to make the proteins that would make this complex system function (evolution from top to bottom in Fig. 6)?

As negibacteria can evolve murein walls predominantly much thicker than usual (e.g. *Deinococcus*), while still retaining thinner Bayer's patch regions to allow the OM to grow, I argued that if such a negibacterium mutated its wall growth machinery so as suddenly to increase its thickness still more dramatically it could overnight become so thick as to break away the OM from its attachments to the cytoplasmic membrane at the Bayer's patches [29,30]. Thereafter there would be no biophysical mechanism for newly made OM lipids to diffuse in a continuous bilayer to regenerate the lost OM, so it was permanently lost and could never reacquire an OM. Most OM proteins would become useless and their genes inevitably degenerate and be deleted. The new unimembranous bacterium was the first posibacterium – the ancestor of all Posibacteria and ultimately neomura also. Thus the initial step of the transition from a negibacterium to a posibacterium could have been very simple mechanistically; loss of the outer membrane could have occurred by a single mutation causing murein hypertrophy. Murein lipoproteins that originally linked the OM to the murein could be retained for linking the thicker wall instead to the cytoplasmic membrane and modified as necessary; a key modification would be the longer retention of the signal peptide to anchor them to the cytoplasmic membrane at least until after they were cross-linked to the murein. As discussed below, all posibacteria have related machinery for achieving this, which establishes their monophyly. In negibacteria the signal sequence must be cleaved after protein secretion to allow the lipoprotein to move to and diffuse within the OM bilayer (with the help of periplasmic chaperones [60]) prior to being cross-linked to murein.

Evolution in the opposite direction from a posibacterium would have required numerous mutations in at least dozens of genes to evolve a lipid, protein, and lipoprotein export machinery; as the closest negibacterial relatives of Endobacteria are Selenobacteria with the exceedingly complex lipopolysaccharide, this would also have had to evolve at that juncture! Of course, this machinery had to have evolved sometime. The key question is: was it mechanistically easier to do so suddenly by the saltatory addition of an extra membrane to a unibacterial cell? Or is it evolutionarily more understandable if it arose more gradually over many generations, and did so in three distinct stages: (1) forming a simple outer membrane with no lipopolysaccharide by differentiation between two pre-existing membranes as in the obcell theory of the origin of negibacteria to make the first Chlorobacteria [30,31], and (2) later becoming more complex by adding the Omp85 mechanism for inserting OM β-barrel proteins in the common ancestor of Hadobacteria and all other life-forms and (3) then evolving impermeable lipopolysaccharide and associated complex secretion/import machinery in the common ancestor of Cyanobacteria and all other life-forms (Fig. 3)? I have long considered a transition from posibacterium to negibacterium to be so difficult mechanistically as to be almost impossible in practice. Apparently only one person has ever tried to suggest how it might have happened: Dawes [61] suggested that the OM could have evolved from the forespore membrane that encloses the spores of typical endospore forming Gram-positives (Teichobacteria) prior to their germination. However this has never seemed plausible to me, as the engulfing forespore membrane could only have been retained as an OM if Bayer's patches and their lipid export machinery and porins all evolved in one cell generation; failing that, such a hopeful monster would immediately have lost the OM again. The problem is even greater than that as the transitional intermediate between negibacteria and posibacteria must have had flagella (Fig. 3). So flagella that originally supposedly evolved in posibacteria would have had immediately to penetrate the saltatorily formed OM, which they now do with the help of a lipoprotein L-ring (see discussion below on flagellar origins). It is hard to accept that the negibacterial mechanisms for both OM and flagellar biogenesis, including a key change in the mechanism of lipoprotein secretion, evolved saltatorily in a single cell generation. Therefore I have long rejected the widespread assumption that unibacteria are ancestral to negibacteria [1,5,29-31,56,62,63]. None of the thousands of implicit supporters of that majority view has ever tried to explain how such an exceedingly improbable transition might have occurred. The onus is on them to do so if they wish to continue to hold that view despite the extensive contrary arguments. Can Dawes' theoretically possible speculation be converted into an evolutionarily acceptable theory? I strongly doubt it; to me it is no more plausible than the other idea he discussed, that the nuclear envelope also evolved from the forespore/spore two membranes, which nobody accepts.

**Figure 7:**
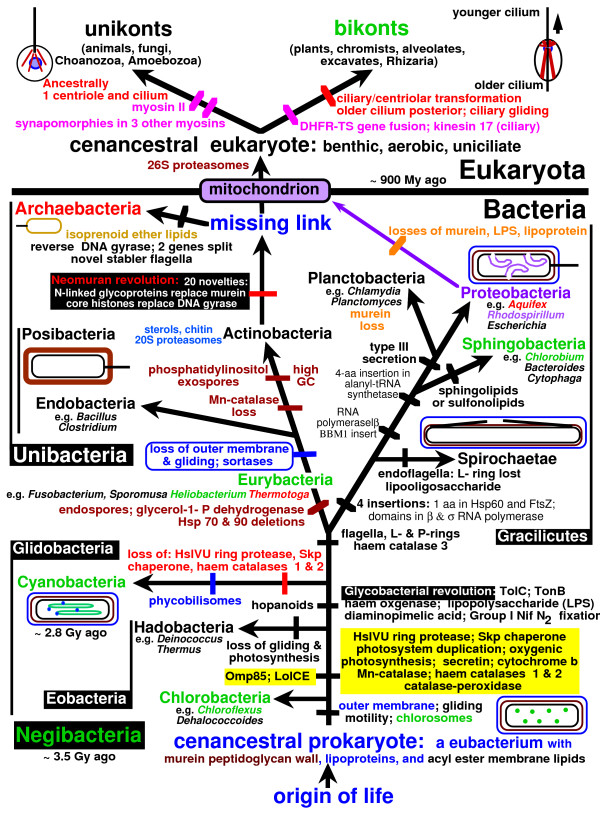
The rooted tree of life emphasizing key novelties and synapomorphies. Thumbnail sketches show major variants in cell morphology (microtubular skeleton red; peptidoglycan wall brown; outer membrane blue). The most likely root position is as shown; the possibility that it may lie within Chlorobacteria instead cannot yet be ruled out. Lowest level groups including or consisting entirely of photosynthetic organisms are in green or purple. The frequently misplaced hyperthermophilic eubacteria are in red; indel analysis confirms that *Aquifex *is a very divergent proteobacterium [79]. The new negibacterial infrakingdom Gracilicutes segregates four phyla from the other negibacteria. Planctobacteria probably lost or reduced murein twice, as free-living Verrucomicrobia have murein. Note that 12 synapomorphies support the earliest branching of Chlorobacteria. The fact that mitochondria were present in the cenancestral eukaryote and that their ancestors, α-proteobacteria are a relatively recently derived of the eubacterial phylum Proteobacteria, proves that eubacteria must be significantly older than eukaryotes and decisively refutes suggestions that eubacteria may be derived from eukaryotes. As α-proteobacteria are nowhere near the root of the tree (irrespective of whether it is rooted beside or within chlorobacteria or as some mistakenly think between neomura and eubacteria) eukaryotes are substantially younger. The age of ~900 My for eukaryotes is based on a recent Bayesian analysis of 143 proteins multiply calibrated from the fossil record [35] and my own critical interpretation of the direct fossil record [129]. This tree, though constructed from rare discrete cladistic characters, is remarkably similar to a 31-protein, 191 species universal sequence tree published while this paper was being reviewed [175]; see responses to comments by referee 3 for discussion of the few differences, all but one (the position of *Aquifex*) in regions poorly supported on the sequence tree.

However one aspect of his theory does seem correct: this is that the transition between negibacteria and posibacteria almost certainly occurred in an endospore-forming bacterium. This is strongly indicated by the fact that Selenobacteria (phylum Eurybacteria: Tables 1, 2; Fig. 7) have a fairly typical Gram-negative envelope with an outer membrane and thin sacculus [64], yet have endospores that are indistinguishable from those of Endobacteria [65-67]. Furthermore they strongly group with and appear to be paraphyletic to Endobacteria on rRNA and protein trees. Thus there is little doubt that the endospore-forming negibacterial Selenobacteria are specifically related to the posibacterial Endobacteria and probably also ancestral to them, in which case the transition did occur in the direction from negibacteria to posibacteria, shown in Figs 2, 3, 5 and 7 and first argued in detail two decades ago [29,30]. Woese, indeed, suggested this for Endobacteria only [68], but neither he nor others have yet accepted that it is true also for actinobacteria [29], as they do not branch with Endobacteria on rRNA trees, though sometimes they do so with Endobacteria plus Selenobacteria. The Selenobacteria/Endobacteria branch is generally called the 'low-GC Gram positives', but this is very misleading in cell biological terms as Selenobacteria have typical negibacterial walls and negative or very weakly positive Gram-staining; moreover not all members of this branch are low in GC). The negibacterial envelope ultrastructure of Selenobacteria such as the heterotrophic *Selenomonas*, *Sporomusa*, and the phototrophic Heliobacteria has been known for some time, which led me to exclude them from Posibacteria and to group them in the phylum Eurybacteria [69] with *Fusobacterium*, which also has a Gram-negative envelope, but unlike the others lacks flagella. Later however, through doubt whether their outer membranes were really related to those of negibacteria, I more conservatively included them and Thermotogales in Posibacteria [1]. Advances in envelope chemistry of *Selenomonas *clearly show that it is a genuine negibacterium, though with significant differences from other negibacteria, e.g. incorporation of cadaverine in its murein and absence of the Braun lipoprotein [64]. Genomic evidence discussed below for *Thermotoga *likewise indicates that its toga is a highly modified OM, so I now exclude it also from Posibacteria. The fact that its toga balloons away from the cell surface may be a consequence of its radically modified peptidoglycan [70] preventing murein lipoproteins from attaching it closely.

Thus the above evidence establishes the origin of Endobacteria from Selenobacteria, but what were the ancestors of Actinobacteria? Are they sisters of or derived from endobacteria, despite not grouping with them on many sequence trees, or did they evolve independently from a separate group of negibacteria? Clearly the proposed mechanism of OM loss by murein hypertrophy is mechanistically sufficiently simple that it might in principle have happened twice. However, as murein hypertrophy is likely also to disrupt cell division, viability would probably need to be simultaneously maintained by independent mutations in the septation machinery. Thus, although murein hypertrophy offers a biophysically very plausible mechanism for OM loss, the generation thus of fully viable offspring could have been evolutionary very difficult (though much less so than a hypothetical transition from posibacterium to negibacterium) and thus relatively late and unique in history. One major feature of the biogenesis of the similarly thick walls of actinobacteria and endobacteria strongly favours a common origin. This is the possession of a universal mechanism for covalent anchoring of surface proteins to the cell wall of Posibacteria. This requires sortase enzymes, which are extracellular transpeptidases positioned in the cytoplasmic membrane. Surface protein precursors that enter the secretory pathway via N-terminal signal peptides have specific C-terminal sorting signals with an LPXTG motif or related recognition sequences, which stimulate sortase-mediated cleavage and the covalent attachment of their C-terminal end to murein peptidoglycan cross-bridges. Genomes of all Posibacteria encode multiple sortase genes, which have diversified to use multiple different substrate classes with different sorting signal motif sequences, and are involved in anchoring a diverse array of structures, including pili on the posibacterial surface [71]. Sortase diversity is greatest in Endobacteria, which have four different sortase classes; Actinobacteria have only two sortase classes, one shared with Endobacteria [71]. The chlorobacterium *Chloroflexus *has one protein with an N-terminal region homologous with sortases, but there is no evidence that it acts as a sortase. A small subclade of proteobacteria has one sortase-related enzyme, which does not fall into any of the five posibacterial sortase paralogue classes, plus a few proteins with a putative sortase recognition motif; it may be a very divergent sortase, but biochemical evidence for such a role is wanting [71]. Homologues of sortases are otherwise entirely unknown from negibacteria despite scores of complete genomes being now available.

Phylogenetic analysis is needed to see whether the isolated proteobacterial sortase-like proteins could have been acquired by lateral transfer or are rare relics of a negibacterial ancestor of the posibacterial sortase family. Present evidence suggests that a major diversification of sortase enzymes, and possibly even the origin of the whole sortase-based protein attachment mechanism, took place in a common ancestor of Actinobacteria and Endobacteria at the time when murein thickening eliminated the outer membrane. Sortase family 3 [71] is a clear synapomorphy for Posibacteria. Overall the evidence is consistent with the view that Actinobacteria are either sisters of or derived from Endobacteria and that their failure to group together on most sequence trees is a phylogenetic artefact.

Although I have argued above (and present even more compelling arguments below based on flagellar evolution) that posibacteria evolved from negibacteria, it is important to note that it is not quite so evolutionarily difficult as I once thought [29,30] to add some kind of extra outer lipid membrane to a basically unimembranous cell. This is shown both by the case of the archaebacterium *Ignicoccus *discussed in a later section and by the presence in mycobacteria and corynebacteria (which form a related subgroup of actinobacteria) of a unique outer lipid layer. Although its structure is less well known than is the much simpler OM of negibacteria, it is clear that this 'mycomembrane' is chemically and structurally utterly different from the negibacterial OM and has evolved independently [72]. The lipids are not phospholipids but mycolates or corynomycolates [73,74] and the lipid layers are thicker. Polysaccharides are abundant outside it as are lipopolysaccharides, but these are chemically unrelated to and should not be confused with those of negibacteria. The major cell wall carbohydrates are arabinogalactans [73], as in plants. Although the protein channels that allow nutrient uptake through this thick impermeable layer are misleadingly called porins they are unrelated to the porins of Negibacteria in sequence or structure [75]. The most abundant mycobacterial porin MspA has a much longer cylindrical pore than negibacterial porins and no clear protein relatives in any other group [76,77]. Far from weakening the contrast between negibacteria and posibacteria, the existence of the non-homologous mycomembrane, which clearly evolved in response to the same selective pressures for impermeability as the negibacterial lipolysaccharide (during the origin of glycobacteria – see Fig 5 legend – as discussed in detail in a later section) shows that such selective pressures do not necessarily produce a membrane with the specific properties of the glycobacterial/negibacterial OM. Thus the fact that all negibacteria except Chlorobacteria have a common mechanism for targeting their OM β-barrel proteins (discussed in detail later), which is not found in these actinobacteria or *Ignicoccus *is very strong evidence for their monophyly. What the existence of the mycomembrane does mean, however, is that arguments for the ancestral character of the negibacterial envelope must rest on the new polarizations within the tree discussed in this paper, not on the original argument based on the difficulty of adding a second membrane [29,30]. Thus it is not the number of membranes per se that is important but their structure and biogenetic mechanisms; this allows us easily to distinguish homology (within negibacteria) and analogous convergence (*Ignicoccus *and mycobacteria/corynebacteria). Despite such superficially similar convergence the distinction between negibacteria and posibacteria remains fundamental.

### Monophyly of Posibacteria

The monophyly of Posibacteria plus Eurybacteria is weakly shown by some 16S rRNA and protein trees, but is often absent from single or multigene protein trees (but seldom more than weakly or moderately contradicted); commonly it is broken by a usually weak association of Cyanobacteria and Actinobacteria, which seems devoid of biological rationale but may reflect base-compositional similarities. The failure of posibacteria to form a clade on multigene trees might be taken as evidence that they each independently lost the outer membrane, but the shared sortase mechanism for covalently attaching lipoproteins to their thick murein walls discussed above renders this highly improbable. It seems more likely that Actinobacteria are excluded from the Endobacteria/Eurybacteria (usually misleadingly called the low GC Gram-positives) by their exceptionally high GC content. Perhaps also a systematically elevated rate of molecular evolution may draw them towards their true relatives, the neomura, which have very long branches on trees for all molecules drastically modified during the neomuran revolution [1]. I see no other way of reconciling the compelling evidence from cell wall and proteasome evolution with most sequence trees. If Endobacteria and Actinobacteria diverged almost immediately after the origin of posibacteria, such biases would probably overwhelm any historical signal for their relationship, a phenomenon known in eukaryotes [2,46] – if the bias is sufficiently strong, this artefact could even happen if Endobacteria are paraphyletic ancestors of actinobacteria, which might therefore be substantially younger than many sequence trees suggest; the contradictory branching order among the three glidobacterial phyla, Endobacteria/Eurybacteria, and Actinobacteria seen in different single and multigene trees is another reason for not taking any of them too seriously. Exoflagella without an L-ring [78] for binding the OM are a synapomorphy for Posibacteria, as are their single envelope membrane of acyl ester lipids and the sortase machinery.

**Figure 8:**
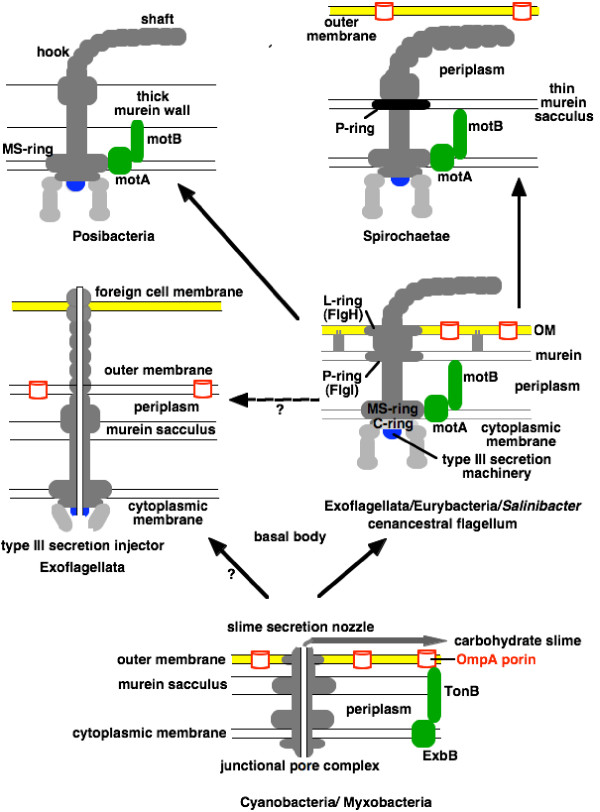
Schematic comparison of the three different basal body structures of eubacterial flagella with the putative ancestral junctional pore complex and the related type III secretion injector. The exoflagella of Proteobacteria and Planctobacteria (Exoflagellata), Sphingobacteria, and Eurybacteria project through the outer membrane, with which they are associated by a lipoprotein L-ring (made of FlgH protein units). Spirochaetes have endoflagella within the periplasmic space that do not penetrate the outer membrane and thus need no L-ring. Exoflagella and spirochaete endoflagella both have a P-ring (made of FlgI protein units) thought to act as a bushing for free rotation within the thin peptidoglycan wall (sacculus). Both P-ring and L-ring are absent from the exoflagella of Posibacteria (Actinobacteria and Endobacteria). Posibacterial flagella would automatically have become external when the ancestral outer membrane was lost. The more complex multiprotein shaft of spirochaetes, clearly a derived character (see text) is shown by its greater thickness. If junctional pore complexes also use a basal type III secretion apparatus, flagella and type III injectors probably evolved from them independently. If junctional pore complexes lack type III secretion homologues, it is likely that they evolved during the origin of flagella only and that type III injectors evolved later in the ancestral exoflagellate by simplification of flagella (dashed arrow); see text for discussion. The diagram assumes that ExbB/TonB/OmpA only associated with the basal body of the flagella and evolved into the flagellar stator MotAB during the origin of flagella.

Substantial shared deletions in chaperones Hsp90 and Hsp70 of all Posibacteria compared with all negibacteria except Eurybacteria, e.g. *Fusobacterium *and *Thermotoga *[79] suggest that Endobacteria, Actinobacteria, and Eurybacteria are all related (Fig. 8) and that sequence trees that group either Endobacteria or Actinobacteria with cyanobacteria or eobacteria that lack these deletions are artifactual. Actinobacteria and Endobacteria are also the only bacteria with resistant resting spores, except for myxobacteria; sporulation and spore germination programmes are so complex that spores probably evolved once only in their common ancestor [1]. These developmental programmes of endobacteria and actinobacteria should be compared in detail to check that they are synapomorphic for Posibacteria. That actinobacterial spores are exospores and less resistant than endobacterial endospores does not preclude a direct relationship; in fungi basidiomycete exospores and ascomycete endospores ultimately had a common origin. If posibacterial endospores and exospores are related, endospores must be the ancestral state, as the unique mode of origin of the endospore by a forespore cell engulfing its sister evolved prior to the divergence of the eurybacterial Selenobacteria and Endobacteria, and on sequence trees Endobacteria nest within Selenobacteria. This developmental mechanism by forespore engulfment is so complex that it is unlikely to have arisen convergently. As endospore formation existed prior to the loss of the OM and origin of the sortase machinery by the ancestral posibacterium, actinobacterial exospores must either have been derived from endobacterial endospores or, less likely, evolved independently.

The recent demonstration that the most early diverging actinobacterium, the filamentous *Symbiobacterium*, has endospores and highest BLAST hits to Endobacteria for nearly half its conserved proteins [51] has now demolished the classical distinction between Endobacteria and Actinobacteria [1,49]. *Symbiobacterium *is a high-GC Gram-positive with posibacterial envelope structure [50]. Unless its strong grouping with Actinobacteria on 16S rRNA trees [50] which showed Posibacteria as holophyletic, but with weak sampling of outgroups rather than low-GC Gram-positives, is a base composition artefact, it indicates that Actinobacteria evolved their high-GC genomes before losing endospores and thus confirms that they evolved from ancestors with the endobacterial phenotype. If we use presence of endospores and highest BLAST hits to the most proteins to demarcate between the two posibacterial subphyla, they would be Endobacteria. If we used their high-GC genomes, grouping on 16S rRNA trees, and filamentous phenotype, they would be Actinobacteria. Thus the classical distinction between Endobacteria and Actinobacteria has now become entirely arbitrary and any justification there might once have been for placing them in separate phyla is now totally gone. Unfortunately they lack both HslVU and proteasomes, so we cannot use them as a demarcation criterion. Perhaps the presence of phosphatidylinositol in Actinobacteria but not Endobacteria may provide the best distinction and synapomorphy for Actinobacteria.

One previously suggested synapomorphy for Posibacteria is unsound. Three-amino-acid insertions in SecF [79] have entirely different sequences in Actinobacteria and Endobacteria and are not even universal in Endobacteria; as similarly unrelated insertions occur at precisely the same location in a few scattered negibacteria, there have been multiple unrelated insertions at that site.

Although I have argued that Posibacteria are monophyletic (having a single origin), they cannot also be holophyletic (i.e. additionally including *all *their descendants), because neomura evolved from them; thus Posibacteria are paraphyletic, but none-the-worse for that as a taxon: they are an excellent taxon, but *not *a clade – taxa must not be polyphyletic but can permissibly be either paraphyletic or holophyletic: see [69] for a discussion of these important distinctions. Much more study is needed of early diverging actinobacteria to establish the sequence of evolutionary events, not only to provide better criteria for classification of Actinobacteria (a recent attempt to subdivide them into classes was premature and probably not phylogenetically sound [1]), but also to determine the precise position on the tree at which neomura evolved from them; four characters of those that pointed to an actinobacterial ancestry for neomura [1] are so far known only in Actinomycetales (proteasomes, chitin, cholesterol biosynthesis, H1-histone-like basic proteins), making it likely that neomura evolved after the early actinobacterial divergences and are sisters of or derived from Actinomycetales only. A fifth typically eukaryotic character, the calcium-binding regulator calmodulin, has clear relatives not only in actinobacteria as earlier thought [1], but also in cyanobacteria and even a few proteobacteria; as there are no convincingly strong BLAST hits in archaebacteria and the rare α-proteobacterial sequences are much more divergent than the actinobacterial or cyanobacterial ones, calmodulin probably entered eukaryotes vertically from actinobacteria [1] or by lateral gene-transfer from cyanobacterial prey in the transitional prekaryote.

### Fusobacteria as relatives of Posibacteria: key evolutionary importance of Eurybacteria

Fusobacteria are especially significant for understanding the negibacteria-to-posibacteria transition as they are morphologically clearly negibacteria with an OM with Omp85, but more of their genes appear related to posibacteria (mostly to Endobacteria), than to other negibacteria (mostly to Gracilicutes, especially Proteobacteria) [80]. The simplest interpretation of this superficially puzzling contrast is that *Fusobacterium *is close to the boundary between posibacteria and negibacteria and to the origin of Gracilicutes, but cladistically closer to Posibacteria (Fig. 7). Such sisterhood is supported by synapomorphic indels in Hsp70 and Hsp90 chaperones [81]. There are two simple explanations of their unusual contrasting pattern of BLAST hits for different genes. First, variation in degree of divergence of different gene lineages after the unibacterial/gracilicute split, secondly the loss of a large number of negibacteria-associated genes when the ancestral posibacterium evolved from a negibacterial ancestor after the posibacterial and fusobacterial lineages diverged; Mira et al. identified 28 such clusters of genes [80]. Rejecting OM loss solely because it implies strong conservation of OM-related genes after the gracilicute-fusobacterial split [80] is invalid; many are very strongly conserved. One need not invoke lateral gene transfer on nearly such a large scale as recently suggested [80]. In particular, it is biophysically highly implausible that its ancestor originally had one membrane and acquired an OM by such transfer [80]. Postulating lateral transfer of 28 clusters of genes with OM-related function is also dramatically less likely than any documented case of lateral transfer.

Grouping Fusobacteria instead with Gracilicutes because of similar insertions in RNA polymerase [82] was also unsound. One 'insertion', in the β'-subunit, is not really an insertion but an internal triplication of a pre-existing SBHM domain. Tandem multiplication of a pre-existing domain is mutationally much simpler and more likely to be convergent than insertions at one site. At the same position the typically single SBHM was thus multiplied to an octomer in the ancestral cyanobacterium, clearly independently [82]. The tree for the β'-subunit strongly excludes *Fusobacterium *from Gracilicutes [82], thus clearly supporting convergent duplication, not shared ancestry for this triplication. The second 'synapomorphy' is a BBM2 insertion in the β-subunit, but even the authors [82] propose four independent BBM2 insertions into that half of the molecule (three closely clustered) and two others into the β'-subunit. *Thermotoga *alone has four such insertions, two unique and one at the same position as the supposed *Fusobacterium*/gracilicute synapomorphy. The β-subunit tree strongly excludes *Thermotoga *from the gracilicute branch, but not *Fusobacterium *[76]. They accepted the independent insertion of BBN2 at the very same site in *Thermotoga*, but not for *Fusobacterium*; however, according to my present interpretation (Fig. 7), *Thermotoga *and *Fusobacterium *are mutually related and might even be sisters; if so, only one such insertion occurred in their common ancestor. The precise position of *Fusobacterium *on the β-subunit RNA polymerase tree [82] may therefore be a tree-construction error. The α-subunit RNA polymerase tree strongly excludes *Fusobacterium *from Gracilicutes [82]. Grouping them together as Group I [82] was unjustified, as was the suggestion of lateral gene transfer. Notably, *Fusobacterium *does not share the long gracilicute insertion in the D/σ^70 ^subunit [82]. Genome sequences of other genera of Fusobacteria are needed to confirm that they are cladistically closer to Posibacteria than to Gracilicutes.

Because of their unique combination of characters Fusobacteria cannot be placed in Posibacteria, as sometimes suggested [79] or in Gracilicutes. I previously grouped Fusobacteria with Selenobacteria as the negibacterial phylum Eurybacteria [69]. I now modify Eurybacteria by adding Thermotogales, which now must be excluded from Posibacteria because the discovery of flagellar L-ring and P-ring proteins and OmpA-related OM proteins unambiguously establishes their negibacterial envelope character. *Fibrobacter *however, originally also included, as early rRNA trees failed to establish its affinity with any other negibacterial phylum, is now transferred to Sphingobacteria based on strong protein indel evidence [83] and weaker confirming evidence from the latest 16S rRNA tree . Thus Eurybacteria now comprise three distinct groups, ranked here as classes: the phenotypically conservative Selenobacteria and Fusobacteria and the phenotypically aberrant Togobacteria. Selenobacteria comprise the always endospore-forming photosynthetic Heliobacteria, the endospore-forming free-living heterotrophs like *Sporomusa *and gut endobionts like *Selenomonas*. The *Heliobacterium *genome project now under way should reveal whether its flagella also have P- and L-rings, as expected, and provide a host of genes that ought to stabilise sequence trees and further test the proposed relationship between these three classes. The evolutionary importance of the negibacteria-posibacteria transition is so great that we need extensive genomic and deeper cell biological studies of a much greater diversity of Selenobacteria, which offer the key to understanding this major quantum evolutionary step: the most important of all in bacterial evolution other than the still more dramatic neomuran transition and the origin of the negibacterial cell itself.

### Coevolution of flagella and cell envelope structure

The hollow eubacterial and solid archaebacterial flagella are unrelated [84,85] and evolved separately. Eubacterial flagella are a synapomorphy for Posibacteria, Eurybacteria and Gracilicutes; their great complexity depends on about 50 different proteins (including regulatory ones; for review see [86]) and was a reason for excluding the root from all three groups [1]. But it was not a decisive one, for flagella have been repeatedly lost within them all and therefore, in principle, one or more of the three glidobacterial phyla that never have them might also have lost them. Here I introduce new evolutionary arguments that polarize the direction of evolution from negibacteria to posibacteria much more decisively than previously and which stem from the fact that posibacterial, *Thermotoga*, and gracilicute flagella are undoubtedly homologous and that the transition between Negibacteria and Posibacteria must therefore have involved an intermediate stage that possessed flagella. I propose a new explanation of flagellar origin: that flagella first evolved in the common ancestor of eurybacteria and Gracilicutes by combining in a novel way two pre-existing macromolecular complexes of the glidobacterial cell envelope.

As Fig. 8 makes clear, there are three fundamentally different structural relationships between flagella and the cell envelope in eubacteria, and three correspondingly different basal body structures. The flagella of Posibacteria are structurally simpler than those of the gracilicutes (spirochaetes, sphingobacteria (*Salinibacter *only) proteobacteria, planctobacteria) and *Thermotoga*, because they lack the P-ring that acts as a bushing for allowing free rotation within the thin peptidoglycan layer (the sacculus) that characterises all gracilicutes, except those Planctobacteria that have secondarily lost it. Unlike in Posibacteria, flagella of Exoflagellata (Proteobacteria plus Planctobacteria), *Salinibacter*, and *Thermotoga *have an L-ring [78], formed by FlgH lipoprotein, which embeds them neatly into the OM in a manner allowing free rotation.

I shall argue that the ancestral condition was as in exoflagellate, *Salinibacter*, and eurybacterial flagella with both an L-ring and a P-ring. The L-ring was lost when the flagellar shaft was secondarily internalised to the outer periplasm by the spirochaete cenancestor. The cenancestral posibacterium lost the L-ring when it completely lost the OM in which it is embedded and lost the P-ring when the thin murein sacculus with which it interacts was drastically thickened. This polarisation of flagellar diversification is entirely consistent with the negibacteria-posibacteria transition in envelope structure discussed above. The evolutionary origin of the flagellum itself provides an independent powerful argument for the root being in negibacteria. Fig. 8 illustrates the new explanation for the origin of flagella. Eubacterial flagella have two fundamentally distinct parts with different evolutionary relationships that had to come together to make the first functional proton-driven rotary flagellum. The first is the basal body – the rotor, which is definitely related to the type III secretion injection apparatus of Exoflagellata [87], and as I propose here for the first time, possibly also to the slime-secretion nozzle of cyanobacteria used for one of the two major forms of gliding motility. The second is the proton conductor stator that provides the rotary force, which is clearly related to the TonB/ExbB complex that in many negibacteria couples cytoplasmic membrane protein motive force to import across the OM. I suggest that the first flagellum originated by the novel combination of a slime-secretion nozzle and a modified TonB complex that became coupled to it, thus causing it to rotate. Later the tip of the nozzle evolved into the hook and shaft of the flagellum. As both components that combined to make the flagellum are separately present only in Negibacteria, flagella must first have evolved in negibacteria. Thus both the posibacterial flagella and their thick walls must be secondarily derived. This argument therefore decisively excludes the root from Posibacteria.

### Evolutionary relationship of eubacterial flagella and type III secretion injectors

Flagellins that constitute the main flagellar shaft and hook components are secreted not by the signal mechanism, universal for life – having arisen prior to the cenancestor [30,31,88], but by transport of leaderless proteins through a hollow tube to the growing tip [78]. This flagellar protein secretion system is made of eight proteins at the base of the hollow basal body, which are conserved among all three types of eubacterial flagella and were clearly present in their common ancestor. Seven of these are also conserved in the type III secretion system used by parasitic members of Exoflagellata to inject proteins into host eukaryotic cells [86]. The single protein not thus shared (FliO) might be optional as it appears not to be present in the lateral flagella of *Chromobacterium violaceum *and *Vibrio parahaemolyticus *(but is present in their polar flagella) or in *Aquifex aeolicus*; its apparent absence from these lateral flagella must be secondary, suggesting that it was also secondarily lost by *Aquifex *(such loss is not a necessary consequence of hyperthermophily as it is present in *Thermotoga*). FliH also appears to have been lost by *Aquifex*, *Caulobacter *and some lateral flagella [86]. There are also structural and sequence similarities between flagellins and the type III injection tube subunits. Overall the type III secretion system is clearly related to eubacterial flagella but markedly simpler (~20 proteins [87]), partly because it does not need so many regulatory proteins and lacks the stator of the motor that drives rotation (MotAB in proton-driven flagella; PomAB in sodium-ion-driven flagella). A complex rotating pentameric HAP2 cap prevents flagellar subunit loss to the exterior during tip assembly [78]; the type III system must avoid wasteful loss of subunits to the environment more simply.

The greater simplicity of the type III system stimulated the suggestion that it might be ancestral to flagella; as the system is also present in a few free-living bacteria it could be more ancient than eukaryotes, against which the best studied examples are all directed, so that suggestion is not totally implausible. If flagella did evolve from type III secretion injectors, it would place the root of the whole tree of life within Exoflagellata, as only they are known to possess it. An alternative possibility is that flagella are the older state and that the type III system evolved from them, which would conversely place the root outside Exoflagellata, prior to their divergence. As flagella and type III injectors each has unique proteins not shared by the other, it is much more parsimonious to suggest that neither is directly ancestral to the other, but that each evolved independently from a simpler common ancestor with a different function from both and that each added different novel proteins to the ancestral core in the process of adapting it to new functions [87]. What this ancestral structure is has not yet been established by sequence or structural evidence. The most likely candidate in my view is the nozzles that secrete carbohydrate fibrils onto the substrate to mediate gliding motility in Cyanobacteria [89] and in the myxobacterial Proteobacteria. The idea that gliding motility may have preceded flagellar motility is not new, but the following explanation of how one form of gliding motility could have evolved into flagella is.

### Gliding motility and the origin of eubacterial flagella

Gliding motility occurs in Chlorobacteria, Cyanobacteria, Eurybacteria, Sphingobacteria, and myxobacterial Proteobacteria, but not in Unibacteria; probably at least three different secretory mechanisms are involved [78]. Some cyanobacteria glide by secreting slime by junctional pore complexes [89], whereas others glide by extension, adhesion and retraction of type IV pili related to those responsible for twitching motility in Proteobacteria [90]. Myxobacteria also have two forms of gliding; social gliding depends on type IV pili at the front of the cell and adventurous gliding uses slime secretion through OM pores at the rear [90,91]. Gliding in much faster in Flavobacteria and other Sphingobacteria than in Proteobacteria; it depends on an ABC transporter in the cytoplasmic membrane and on outer membrane lipoproteins; the proteins involved seem unrelated to those responsible for gliding in myxobacteria or to flagella [92]. Sphingobacterial gliding is probably unique to the phylum and a derived state: I argue in a later section, when discussing lipopolysaccharide evolution, that its origin was associated with changes in cell envelope lipid composition and the loss by most of them of flagella, widespread characteristics of the phylum – it used to be thought that flagella are always absent from Sphingobacteria [1], but they are present in the newly described halophile *Salinibacter *[93], which supports other reasons given later for thinking that Sphingobacteria were ancestrally flagellate, like all Gracilicutes. The molecular nature of the slime secretion machinery is unknown in Cyanobacteria and Myxobacteria, but they both have hollow cylindrical junctional pore complexes embedded in the cell envelope from which the slime is actively extruded like toothpaste from a tube [89,91]. In cross section the extrusion nozzles look very similar, but only the cyanobacterial ones have been seen in lateral view. Remarkably they appear to consist of four stacked hollow rings [89], just as does the basal body/rod region of eubacterial flagella. I consider it improbable that two such stacked ring structures evolved entirely independently and therefore propose that such a structure was present in the common ancestor of Cyanobacteria and Myxobacteria, and that flagella evolved directly from it to generate the immediate common ancestor of Gracilicutes and Eurybacteria. Later in the common ancestor of Exoflagellata the junctional pore complex evolved independently into Type III secretion injectors. This assumes that a primitive form of type III secretion machinery will be discovered in association with junctional pore complexes; if it is not, then I would argue that the injectors evolved instead from flagella by simplification. All the genes shared by flagella must have undergone gene duplication prior to that common ancestor, as it initially must have retained both systems (to explain the presence of both in Proteobacteria, assuming no secondary acquisition of pore complexes by lateral gene transfer).

If this is correct, it would establish that flagella evolved in negibacteria not posibacteria, and therefore that the root of the tree is also within negibacteria. Two further arguments make this conclusion inescapable. These concern the origin of the flagellar motor.

### Negibacterial origin of eubacterial flagellar motors

Flagellar rotation can be driven by protons or sodium ions. The proton- and sodium-driven motors are separate and can coexist in the same cell. Both probably evolved early in flagellar evolution as posibacteria and Gracilicutes have both types. Both have two components: an ion channel (MotA or PomA respectively) in the cytoplasmic membrane, which associates with ancillary proteins and the outside of the bacterial rotor so as to drive it; and a protein that binds to the ion channel and attaches it rigidly to the murein cell wall to prevent it from rotating (MotB or PomB [86]). I now argue that these components of the motor evolved from functionally and structurally similar proteins of the TonB complex, which is found exclusively in Negibacteria (never Unibacteria). It is absent from the two negibacterial phyla here argued to be the most primitive (Hadobacteria and Chlorobacteria), but would have been present in the putatively non-flagellate common ancestor of Cyanobacteria and Gracilicutes/Eurybacteria. Loss of the TonB complex during the origin of Posibacteria by OM loss is to be expected as its function is to transmit energy from the cytoplasmic membrane ion channel (the ExbBD complex) to OM β-barrel proteins involved in ligand import or signal transduction (so called Ton-B-dependent receptors [59]); clearly with the receptors gone TonB was useless and quickly lost also. TonB spans the periplasmic space, just like MotB, and like MotB binds a proton channel in the cytoplasmic membrane proximally, binds to the murein wall within the periplasmic space, and to OM proteins distally. Thus it is functionally the same as MotB but not obviously related in sequence.

Mot A is clearly homologous in sequence to ExbB [86] and I suggest evolved from a gene duplicate of it during the negibacterial origin of flagella. The proximal proton channel binding domain of MotB has no obvious relatives, but I suggest that it evolved from the corresponding domain of TonB and diverged so substantially during the remodelling necessary to attach the complex to the basal body that this derivation is no longer reflected in its sequence; 3D structure might be more similar. The distal domain of MotB is very different from TonB, but is related to OmpA, a widespread negibacterial OM protein. I suggest that MotB first evolved by gene fusion between duplicates of the TonB and OmpA genes. This would have differentiated its OM attachment properties from those of TonB and prevented it from interfering with the latter's specific binding functions to OM importers/transducers. As Posibacteria lack an OM (as discussed above the outer lipid layer of Mycobacteria is unrelated) or freestanding OmpA paralogues, they could not have provided such a domain to the ancestral MotB. Thus neither of the ancestors of the flagellar motor proteins (TonB complex, OmpA) was present in Unibacteria, so the proton-driven stator of eubacterial flagella must first have evolved in a negibacterium. If ancestors of both the stator and, as argued above, the basal body rotor (i.e. slime-secretion nozzles) were present only in negibacteria, clearly they must have come together in negibacteria. This proves independently of the powerful cell wall arguments discussed above that negibacteria were ancestral to posibacteria, not the reverse.

Merely modifying the TonB complex in the manner just described would be sufficient to make it interact with the basal ring of a pre-existing secretion nozzle and to cause it to rotate. Assuming that the cell remained only locally attached to the substratum by the slime this would cause the whole cell to rotate as seen today in bacteria tethered by their flagella. Such whole-cell rotation rather than swimming is likely to have been the original function of flagella as it would not require any complicated controls such as reversibility or directionality of swimming or chemotaxis, phototaxis or gravity or magnetic reception to become immediately useful. Merely rotating would stir the boundary layer close to surfaces and make nutrient uptake more efficient, providing an immediate selective advantage for evolving the flagellum even prior to the evolution of the flagellin shaft. Thus simply coupling a pre-existing proton channel, already fixed to the murein by TonB and able to transmit a shearing force to the outside of the rotor would in one evolutionary step generate a functionally beneficial protoflagellum; such coupling could in principle have occurred by modifying just one protein associated with the ExbBD complex.

### Evolution and diversification of the flagellar hook and shaft

Swimming could then simply evolve by multiple gene duplications and divergences to generate all the flagellar rod/hook proteins and the helical flagellin shaft. The rod that attaches the hook to the basal body is made of four mutually paralogous proteins (FlgB, FlgC, FlgF, FlgG [86]). The hook is made of FlgE and the first hook-filament junction protein is FlgK, both related to the rod proteins, showing that all six arose by successive gene duplications of a single common ancestral gene. An unrelated gene FliK regulates hook length; as its sequence is related to that of YscP, the similarly functioning molecular ruler for the injection tube of type III injectors [86], it is likely that precursors of the rod/hook proteins and a common system of length regulation were already present in the common ancestor of both – here argued to be a slime-secretion nozzle. The second hook-filament junction protein (FlgL) is related to the shaft (filament) protein flagellin (FilC) [80] and probably gave rise to it by gene duplication. Spirochaete flagella are more complex than others in having three flagellins (typically only one flagellin in the rest; *Caulobacter *is a notable exception with several) surrounded by a fourth sheath protein [94], and two or four FliG homologues not one [86]; FliG is one of the three proteins making the C-ring at the base of the basal body – the others are FliM/N, necessary for rotation and the flagellar switch [86].

This much greater complexity of spirochaete basal bodies and shafts is a synapomorphy for spirochaetes, which means that their endoflagella must be derived rather than ancestral; thus the ancestral negibacterium cannot have been a spirochaete. Typical spirochaetes such as *Treponema *lack a lipoprotein L-ring, which was obviously lost when they evolved endoflagella that unlike ancestral flagella no longer penetrate the OM. Curiously, unlike them, the early diverging *Leptospira *retains the L-ring protein FlgH [80]; irrespective of whether this means that it also still retains an L-ring unlike other spirochaetes, despite periplasmic internalization, or instead that this protein has become detached and acquired a new function in the OM or elsewhere, this provides further good evidence that spirochaetes evolved secondarily from ancestors with exoflagella and L-rings, and proves that L-rings can be lost as postulated for the ancestor of Posibacteria.

That Planctomycetes have secondarily lost peptidoglycan and replaced it by a protein exoskeleton is now clearly shown by the discovery of relict enzymes for murein peptidoglycan synthesis [95], thus confirming the theory that the ancestral eubacterium already had a murein peptidoglycan wall [30]. The planctomycete genome sequence [95] also revealed the presence of P-ring genes even despite the loss of the peptidoglycan sacculus. As Planctomycetales probably lost peptidoglycan hundreds of millions, possibly billions of years ago, this suggests that loss of the P-ring is evolutionarily very difficult, unless either the L-ring, or the entire flagellum is also lost as in most Sphingobacteria. Biogenesis of the more distal part of the flagellum, including the L-ring, has apparently become developmentally dependent on the P-ring proteins; mutations in FlgI protein are highly disruptive, and when that protein is eliminated the L-ring protein (FlgH) is destabilised and cells are immotile. Blocking lipoprotein synthesis inhibits flagellar assembly at a much earlier stage than L-ring assembly, indicating that lipoproteins are fundamentally important for proteobacterial flagellar biogenesis.

### Sphingobacteria ancestrally had flagella

That all Sphingobacteria evolved from ancestors that once had flagella is suggested by the presence throughout non-flagellate members of the phylum of a protein related to MotB with a C-terminal OmpA domain, but faster evolving that MotB, as shown by my BLAST study, but mutually more closely related than to proteins in other phyla. MotA homologues could not be detected in non-flagellate Sphingobacteria (but are present in *Salinibacter*, which exceptionally for sphingobacteria has flagella [93], as are the usual flagellar proteins expected for negibacterial exoflagella, such as L- and P-ring proteins and at least two proteins that though annotated only as OmpA family/domain proteins could be MotB or MotB relatives [96]; affinity of *Salinibacter *flagellar proteins with those of δ- and ε-proteobacteria is expected from the phylogeny of Fig. 7, so was insufficient reason for suggesting lateral transfer [96]). The MotB homologue is longer in *Cytophaga*, having an extra N-terminal domain (a tetratricopeptide repeat). In other genera it is usually similar in length to MotB, but in a few flavobacteria an Smc/transforming-acidic-coiled-coil domain, not generally found in MotB, is present within the N-terminal half of the molecule. This diverse pattern of evolution in putative MotB relatives of non-flagellate Sphingobacteria, coupled with the absence of MotA, suggests that they may have evolved from MotB and were recruited for new functions conserved within non-flagellate Sphingobacteria, immediately on the loss of MotA, thus preventing the total loss of MotB. Sequence phylogenies of the proteins might clarify this further.

### Contrasting origins and biogenesis of eubacterial and archaebacterial flagella

I now suggest that the highly probable historical fact that eubacteria evolved in cells with two envelope membranes may help explain some of the differences from archaebacterial flagella, which certainly evolved in cells with only one membrane.

The complex biogenesis shared by flagella and type III secretion [78], would itself probably have been harder to evolve in a unimembranous cell than in a negibacterium with its two bounding membranes. Even the putative precursor of both must have been quite complex and must have evolved a mechanism to prevent loss to the environment of its tip-growing subunits exported to the end through the central hole. I suggest that tip-capping machinery first evolved in its ancestor when an even simpler precursor may have been used to secrete proteins or polysaccharides into the inner periplasm. Later by evolving a specific murein hydrolase, as used by developing flagella to penetrate the sacculus, these could be secreted into the outer periplasm instead. Later still, by evolving a lipoprotein ancestor of the L-ring protein FlgH, the tip of the nozzle could penetrate the OM and secrete slime to the exterior. Thus even the evolution of the relatively complex putative precursor of flagella can be explained in simple mechanistically feasible and individually selectively advantageous steps. Thus, as I asserted previously, there is no irreducible complexity in eubacterial flagella [97]: the most complex macromolecular machine of all in bacteria – creationists, who use it as a spurious argument against evolution [98], take note!

The very much simpler archaebacterial flagella evolved in a unimembranous cell from a type IV secretion mechanism related to eubacterial pili, involving the signal mechanism but a specialised signal peptidase; significantly their flagella are solid and probably assemble at the base, like pili [78], avoiding the leakage problems for initiating type III secretion in Unibacteria. The actinobacterium that evolved into neomura either lacked flagella or they failed to survive replacement of murein by glycoprotein and/or the origin of archaebacterial lipids, or were simply depolymerised by the hot acid environment of the cenancestral archaebacterium [1]. This basal growth by solid flagella may be an essential feature of initial flagellar evolution in a unimembranous cell, as the early stages of evolution of a tip-growing hollow flagellum prior to the necessarily later origin of a cap could have been strongly disfavoured by loss of proteins to the exterior; in a cell with two membranes the initial stages of evolution of the hollow flagellum and cap could have been in the periplasmic space where secreted proteins could accumulate without being totally lost. If so, the hollow eubacterial flagella perhaps could initially only have evolved in a negibacterium with two membranes.

The traditional assumption that cells first had one membrane predated discovery of the OM and its universality in eight of the ten bacterial phyla; given a root outside proteates, that model must assume the origin of posibacterial exoflagella without L- or P-ring prior to the last common ancestor of life and overcome the difficulties emphasized above of adding them simultaneously with the origin of the OM and its secretion mechanisms in any hypothetical transition to exoflagella. It would also fail to have any precursors such as TonB or a slime-secretion nozzle for the evolution of flagella. It must also assume that spores had evolved prior to the cenancestor, but were not retained by the other nine bacterial phyla (unless the probably convergent spores of Myxobacteria were such 'relics'). The first three assumptions are mechanistically implausible and all four selectively implausible. Thus a negibacterial origin of eubacterial flagella and the derivation of posibacteria from negibacteria are very hard to evade.

### Hyperthermophilic eubacteria are derived and not mutually related

Having both L-ring and a P-ring and a peptidoglycan sacculus without cadaverine [80], and an OM without sulfonolipids, provides a combined structural synapomorphy for Proteobacteria (initially established solely from rRNA trees; now also supported by insertions in inorganic pyrophosphatase [9] and RNA polymerase [82]). Proteobacteria, including *Aquifex *– which has flagella, can be unambiguously defined phylogenetically as a clade that ancestrally possessed an outer membrane with lipopolysaccharide but not sulphonolipids, a thin periplasmic murein wall with diaminopimelic acid but no cadaverine, and exoflagella with L-ring and P-ring; only flagella are sometimes secondarily missing. My BLAST analysis of *Aquifex *P-ring and L-ring proteins shows the highest hits with deltabacteria, and lower ones with epsilobacteria, consistent with its classification within Thiobacteria [1]. Thus flagellar evolutionary considerations clearly support the inclusion of both Aquificales and Geobacteria, which have the same envelope structure, in a broadened Proteobacteria [1], which now for the first time has a structural rationale for its circumscription and clear distinction from all other gracilicute phyla.

It has been much debated whether *Thermotoga *is a posibacterium or a negibacterium [1] and whether its thin toga is related to the negibacterial outer membrane or not. Originally I placed it in its own subphylum independently of both Negibacteria and Posibacteria [5], but influenced by indel arguments [81,99], the absence of lipopolysaccharide, and the initial lack of clear evidence for porins or phospholipids in the toga, and its very different morphology from the negibacterial OM, assumed that it had evolved independently and therefore placed Thermotogales in Posibacteria [1,69]. But flagellar evidence now makes this untenable, as does the fact that its major OM protein is a porin related to OmpA of advanced negibacteria. The fact that the *Thermotoga *genome [100] encodes both P-ring and L-ring proteins clearly shows that the toga is related to the outer membrane of gracilicutes, and implies that lipolysaccharide has been lost. The complexity of flagellar structure and biogenesis and its coadaptation to two distinct membranes is so great that it could neither be convergent between *Thermotoga *and Gracilicutes nor acquired by lateral gene transfer. Lateral transfer has given *Thermotoga *many foreign genes [101,102], but these are for soluble enzymes that are useful individually and not a part of a giant macromolecular complex integrated into two distinct membranes. My BLAST analysis of the very conservative L-ring and P-ring proteins of *Thermotoga *shows the highest hits with deltabacteria (and then γ-proteobacteria), but not with *Aquifex*. The grouping of *Thermotoga *and *Aquifex *on some genomic trees may be partially attributable to lateral transfer between them and thermophilic archaebacteria [103]. However, as such grouping is also seen when putatively laterally acquired genes are excluded [22], it probably reflects either strong thermophilic biases and convergence of amino acid composition caused by the exceptionally high GC content of both taxa [1,2] or a genuine relationship between the two genera. The absence of 4–5 characteristic gracilicute insertions from *Thermotoga *(discussed below) makes it unlikely that it is really related to *Aquifex *or other Proteobacteria. Instead the shared presence of glycerol-1-P dehydrogenase with Posibacteria and their grouping together on that gene tree, plus a shared deletion suggest, albeit not decisively, a specific relationship with Posibacteria. As *Thermotoga *is thus probably related to but not actually a posibacterium I place it in the paraphyletic phylum Eurybacteria, from which posibacteria evolved. Overall, it is very likely that the deep branching of both Thermotogales and Aquificales and their grouping together on many sequence trees are both artefacts of convergent hyperthermophilic biases. My placement of each in a derived position in two independent ancestrally non-hyperthermophilic phyla is congruent with other evidence that the ancestral eubacterium was probably not hyperthermophilic [104].

### The argument so far

Let me summarize: proteasome evolution excludes the root from the proteate clade; cell envelope evolution and flagella evolution independently of each other exclude it from Posibacteria; eubacterial flagellar evolution also excludes it from Gracilicutes and Eurybacteria, and places it among the non-flagellate eubacteria that have gliding motility by slime secretion from junctional pore complexes (i.e. Glidobacteria). Later sections will present two further transition analyses that narrow down the position of the root still further within the Glidobacteria. The Omp85 argument is especially strong and excludes it from all negibacteria except Chlorobacteria. I shall also give further evidence that Gracilicutes are derived compared with Glidobacteria. All these polarisations of the tree are congruent. Combining them leaves only two possible places for the root: within Chlorobacteria or between them and all other organisms. Thus Chlorobacteria are either the ancestors of or the sisters of all other organisms, and the last common ancestor of all cells was bounded by two membranes not one. We can conclude that the OM was probably simply made of phospholipids, as in Chlorobacteria, and that murein and lipoprotein were both present, whereas lipopolysaccharide was probably absent.

The OM would have been attached to a peptidoglycan wall of intermediate thickness by murein lipoproteins. Cotranslationally secreted lipoproteins are found in all eubacteria (with the sole and unsurprising exception of Mollicutes, which evolved by losing murein from a teichobacterial ancestor that had already lost the OM) and their special signal peptidase II was clearly present in the last common ancestor of all life. It was lost during the neomuran revolution during the overhaul of the signal recognition particle during the origin of cotranslationally secreted N-linked glycoproteins when the murein wall was lost. There is a pleasing simplicity in the distribution of cotranslationally secreted conjugated proteins across the tree of life. The basal domain eubacteria makes only cotranslationally secreted lipoproteins and has separate signal peptidases for lipoproteins and unconjugated proteins. The derived clade neomura makes only cotranslationally secreted glycoproteins and uses the same signal peptidase for them as for unconjugated proteins. The neomuran revolution was the largest quantum evolutionary change in cotranslational secretion in the history of life and unsurprisingly therefore caused dramatically rapid and unprecedented changes in the translation apparatus itself, i.e. in ribosomal RNA and proteins [1,5,29]. Using the false assumption of a molecular clock, these major transient changes have often been misinterpreted as evidence for an early divergence of neomura and eubacteria. The name neomura (new walls) is entirely appropriate given the six separate polarisations that independently and congruently show that their walls evolved after the peptidoglycan walls of eubacteria: the prokaryote to eukaryote, eubacterial to neomuran, non-proteate to proteate, negibacterial to posibacterial, eobacterial to gracilicute/eurybacterial and chlorobacterial to Omp85-containing negibacteria. That six independent robust polarisations (plus a seventh strong but somewhat less decisive one, also discussed below: the origin of glycobacteria) are all congruent with each other and with the fossil record [1] shows that eobacteria are immensely older than archaebacteria. Thus there is only one primary domain of life: the paraphyletic eubacteria. Eukaryotes and archaebacteria are far younger and holophyletic sisters, secondarily derived from within one eubacterial phylum, the substantially older Posibacteria. Before discussing the polarisations within Glidobacteria more needs to be said about Unibacteria.

### Unibacteria: links between posibacteria and archaebacteria

The subkingdom Unibacteria comprises all bacteria bounded by a single membrane only (i.e. Posibacteria and Archaebacteria), whereas Negibacteria include all eight phyla with an additional outer membrane – the vast majority of bacteria. It has long been clear that Unibacteria are paraphyletic, as eukaryotes (also bounded by a single membrane) evolved from them. Unibacteria and eukaryotes are the only organisms with a unimembranous cell envelope. The arguments detailed above establish clearly for the first time that the unimembranous state is derived, whereas the bimembranous state of negibacteria is the ancestral state for all life, as first suggested by Blobel [57] from considerations of the evolution of cotranslational protein targeting to membranes. Thus the textbook picture of the first cells having but a single membrane is probably wrong. Far from making it more difficult to understand the origin of life, this probably makes it easier [30,31].

It was also clear that Negibacteria are technically paraphyletic long before they were proposed as ancestors of Posibacteria, as mitochondria and chloroplasts both evolved from negibacteria and both retained the OM throughout their enslavement by eukaryote host cells [105,106], even after the whole genome was lost by the hydrogenosome and mitosome descendants of mitochondria [106]. Thus the OM is exceedingly stable in evolution and has only very rarely been lost [29] – just once, making the distinction between Unibacteria [69] with a single bounding membrane and Negibacteria [29] much more important than is usually realised. Conversely the gulf between archaebacteria and eubacteria, although also very important [1], is of less fundamental significance than widely supposed. Most of the special features of archaebacteria that initially led to their separation from eubacteria are major, but often largely quantitative changes in the evolution of a specific subset of their molecules: in DNA-handling enzymes and those related to ribosomes, and not major qualitative cellular innovations. I have argued that these marked and concerted changes stem from only two more fundamental key neomuran innovations; chromatin and co-translational synthesis of surface N-linked glycoproteins [1]. They are puzzling only to those who mistakenly believe in a universal molecular clock and ignore the key importance for cell evolution of quantum evolution (transiently hyperaccelerated change) and intermolecular coevolution. Only three major innovations of substantial biological significance, not shared with eukaryotes, distinguish archaebacteria from posibacteria; novel flagella, discussed above; reverse DNA gyrase, a derived enzyme that evolved by fusion of two ancestrally eubacterial enzymes [1]; and novel isoprenoid ether lipids, all three arguably adaptations to hyperthermophily and acidophily [1]. The difference in lipids from eubacteria has sometimes been fundamentally misinterpreted as evidence for the early origin of archaebacteria, or even the independent origin of neomuran and eubacterial cells according to one extreme view [107,108], which is entirely untenable as it ignores most cell biology [18] and the evidence from proteasomes and a dozen other characters for a relationship between archaebacteria and actinobacteria [1]. The contrast between the eubacterial murein/lipoproteins and neomuran glycoproteins also should not be misinterpreted as evidence for independent origins or even for early divergence between the two. As I pointed out long ago [29] there is an important similarity in biogenetic mechanism that implies some kind of genetic continuity between them. In both cases the oligosaccharide/glycan precursors are assembled while being covalently attached to a polyprenol phosphate carrier in the membrane to which N-acetylglucosamine is the first residue to be attached (always in eukaryotes, usually in archaebacteria). Sequence similarity is detectable by BLAST between the transferase that catalyzes this step in eubacteria and homologues in crenarchaeotes. Furthermore even though the carriers are the much longer dolichol in neomura (undecaprenol or decaprenol in eubacteria) the enzymes that make them are highly conserved in both archaebacteria and neomura [109,110], thus confirming the conjecture of some conservation of surface glycan biogenesis across the eubacterial/neomuran divide [29].

An unavoidable conclusion from the holophyly of proteates demonstrated by proteasome evolution is that isoprenoid-ether membrane lipids replaced acyl-ester lipids during their origin, in agreement with my previous polarization of the transition from actinobacteria to archaebacteria via the neomuran revolution [1,30]. Even during this dramatic change Archaebacteria retained machinery for making and degrading fatty acids [18] and numerous archaebacterial genomes encode proteins homologous to phospholipid synthetases; eubacteria and archaebacteria both make isoprenoids. The idea of an unbridgeable gulf in lipid biosynthesis between them is a myth. What was novel in the first archaebacterium was joining isoprenol to glycerol phosphate with new stereochemistry (to sn-glycerol-1-phosphate, not sn-glycerol-3-phosphate like acyl-ester lipids). All major groups of Posibacteria, plus *Thermotoga*, but no other negibacteria or eukaryotes, have close relatives of the enzyme making this unusual stereoisomer [18]; this glycerol-1-phosphate dehydrogenase is a previously unrecognized synapomorphy for Posibacteria plus some Eurybacteria: invocation of lateral transfer in its history [18] is unjustified. Its presence in both Actinobacteria and Endobacteria is consistent with posibacterial monophyly and provides a modest argument for excluding the root from Posibacteria plus Eurybacteria. I suggest it originated where shown in Figs 3 and 7 by gene duplication from one of three universally distributed sisters [18]; those paralogue subtrees put the root in eubacteria, one between cyanobacteria and the rest, one between Planctobacteria and the rest, and one within Proteobacteria; never between archaebacteria and eubacteria [18], further exemplifying the conflicts stressed above among such trees.

Previously I pointed out that the presence of phosphatidylinositol in all eukaryotes and all actinobacteria, but no other eubacteria was evidence for a specific relationship between actinobacteria and neomura [1], as were 13 other characters in addition to proteasomes (one of these, calmodulin, is now excluded from the list as it has been found in some other eubacteria). I assumed that the ancestral archaebacterium had lost phosphatidylinositol when it evolved isoprenoid ethers. In fact some archaebacteria have isoprenoid ether analogues of phosphatidylinositol [111]; as their CDP-inositol transferases are homologous to those of eukaryotes, it is likely that they also were inherited from actinobacterial ancestors and retained during the changeover from acyl ester glycerophospholipids to isoprenoid ether glycerophospholipids in the ancestral archaebacterium: further evidence of evolutionary continuity in membrane lipid synthesis between eubacteria and archaebacteria. The inositol phospholipids of the myxobacterium *Stigmatella aurantiaca *(δ-proteobacterium) are dialkyl ether lipids [112] and thus not homologous with the acyl ester phosphatidylinositol of eukaryotes and actinobacteria, so do not weaken acyl ester phosphatidylinositol as a sound synapomorphy for actinobacteria plus neomura (given that it was inevitably lost with other acyl esters when the ancestral archaebacterium lost that biosynthetic machinery [1]).

However, another character that was previously used to group Posibacteria with neomura turns out to be less decisive than originally thought: the signal recognition particle (SRP) RNA [1]. Negibacteria have a short hairpin-shaped 4.5S SRP-RNA with only one GTPase protein that binds to universally conserved helix 8 at the hairpin head, essential for co-translational protein secretion. This is probably the ancestral state for all life, and the more complex neomuran double-headed 7S SRP-RNA, with an extra adjacent helix 19 having its own binding protein, is derived, originating later in the neomuran revolution [1]. *Bacillus subtilis *and other Bacillales (Endobacteria) lack helix 19, but have an extra 5' domain resembling the positionally corresponding Alu domain of neomuran 7S SRP-RNA. SRP-RNA specialists regard these domains as homologous [113]; if true it would be a synapomorphy for Posibacteria plus neomura and *Thermotoga *[1]. However, as this extra domain is absent from some Endobacteria (Lactobacillales) and all Actinobacteria [113], either the 3-loop structures are convergent, not homologues (extreme Alu-domain variability in neomura makes homology debatable), or this domain was secondarily lost by Lactobacillales and Actinobacteria (or neomura evolved not from actinobacteria but from endobacteria, which proteasomes and numerous other characters disprove [1]). The presence of an Alu-like domain indistinguishable from that in Bacillales in *Thermotoga *SRP-RNA [113] suggests that this domain may have evolved in the common ancestor of Posibacteria and *Thermotoga*. I stress that although the evidence presented here and previously [1,28] that Actinobacteria and neomura together are a clade is strong, we lack firm evidence that Actinobacteria are paraphyletic ancestors of neomura, as previously assumed [1] and suggested above, and not simply their holophyletic sisters. If extant Actinobacteria were actually holophyletic, only a single loss of the Alu domain in their common ancestor would be necessary on the assumption that this domain evolved in the ancestor of Eurybacteria and Posibacteria. Without stronger evidence for homology between the variable neomuran structures and the more uniform ones of Bacillales/*Thermotoga*, it is unclear whether or not it can be used to argue that Eurybacteria, Posibacteria and neomura together constitute a clade. It would be valuable to seek this domain in SRPs of other Eurybacteria to see whether it is present in all, or just in *Thermotoga*, and also more broadly in deeper branching Endobacteria. Note that in a recent 16S rRNA tree [114], actinobacteria are sisters of archaebacteria plus *Aquifex *and *Thermotog*a; given that this position of *Aquifex *and *Thermotoga *is almost certainly a long-branch artefact, it cannot be argued that the evidence from proteasomes for the holophyly of proteates is contradicted by 16S rRNA trees.

The possibility that Actinobacteria are sisters of neomura, not ancestral to them as previously suggested [1], needs to be seriously considered, as there are 14 proteins found in all sequenced Actinobacteria, but no other organisms (K. Chater pers. comm.), which would favour their holophyly. A sister relationship would also allow the endobacterial glycosyltransferases discussed in the next section and the SRP Alu domain to have been both present in the last common ancestor of neomura and actinobacteria, and each to have been lost only once in the ancestor of actinobacteria.

The outer sheath of the archaebacterium *Ignicoccus *is not an exception to the unimembranous character of archaebacteria, even though it is sometimes misleadingly referred to as an outer membrane [115]. It differs profoundly from the negibacterial outer membrane in chemistry, ultrastructure, and in its general lack of close contact with the cell surface. Although it may contain diether isoprenoid lipids in addition to much protein, its biogenesis differs profoundly from the non-homologous OM of negibacteria and it clearly originated independently. It thus does not lessen the clear-cut separation of unibacteria and negibacteria. Nor does it affect the validity of the arguments from cell wall/OM and flagellar evolution that posibacteria evolved from negibacteria, not the reverse. It appears to be an unusually interesting modification of the crenarchaeote cell wall rather than a true membrane.

### Recency of archaebacteria

I stress that the above arguments concerning evolution of proteasomes, Omp85, flagella, and the cell envelope that together unambiguously root the tree of life within negibacteria are mutually reinforcing and do not rely at all on fossil evidence, yet fully agree with it [1]. Thus criteria of congruence between independent lines of evidence are well met. The proteasome argument uses the principle of paralogue rooting, but relies on the irreversibility of the divergence in structure and function of the core proteasomal subunits to polarize evolutionary direction. It is therefore superior to methods using sequence trees, which gave conflicting and debatable results because of tree reconstruction problems [1,13,116]. The negibacterial root of the tree establishes that the cenancestral cell had acyl ester lipids like eubacteria and eukaryotes. The above analysis disproves wild speculations that archaebacterial and eubacterial cells evolved separately [107,108], which exaggerate differences in their lipid and other biology. It is worth emphasizing four features of membrane biology that were present during the eubacteria to neomuran transition: (1) the membrane-embedded SRP receptor and the transmembrane protein channel for cotranslational secretion (2) membrane division by FtsZ; (3) a respiratory chain with membrane-embedded cytochrome b, plus Rieske protein; (4) a mechanism for transporting hydrophilic carbohydrate wall precursors (which included N-acetylglucosamine residues) across a lipid membrane by means of carrier isoprenols (undecaprenol/dolichol) synthesized by an enzyme that is clearly homologous between eubacteria and archaebacteria. The last point confirms my argument that there was some continuity between the mechanisms of formation of murein peptidoglycan walls by eubacteria and of glycoprotein walls by archaebacteria [29], despite the radical nature of this changeover.

Furthermore, the integral membrane protein subunit of the eukaryote enzyme UDP-N-acetylglucosamine transferase that actually transfers N-acetylglucosamine to dolichol phosphate is distantly homologous to a eubacterial transferase (MurG) involved in murein synthesis, which has two glycosyltransferase domains [117]. However, the eukaryotic protein is even more closely related to the single domain glycosyltransferase that makes the capsular polysaccharide (EpsE: [118]) of certain endobacteria by transferring glucose from UDP-glucose to a C35 isoprenol phosphate carrier; its highest BLAST hits are all to endobacteria, except for one to an archaebacterium, and there are no BLAST hits to any α-proteobacteria; thus this enzyme might have been inherited vertically from the posibacterial ancestor of the host component of the eukaryote cell. As single-domain homologues are so taxonomically restricted, they may have evolved from and coexisted with MurG (present throughout eubacteria) in Posibacteria after they diverged from Proteobacteria by gene duplication and C-terminal truncation. The second, non-membrane subunit of the eukaryotic enzyme appears closest to other posibacterial single domain glycosyltransferases (WciR of *Streptococcus *and EpsF of *Lactococcus*). As these single-domain transferases are all related to the C-terminal end of MurG [117], MurG probably duplicated and split into two separate single domain glycosylases within Posibacteria. Possibly both of them were recruited by the ancestral neomuran for the first step of N-linked glycoprotein synthesis, rather than MurG itself being directly modified as assumed by the original version of the neomuran theory [29]. Despite this difference in detail, this new evidence fits the prediction of vertical inheritance of an integral membrane posibacterial wall synthesis enzyme by eukaryotes during the neomuran revolution [29].

The fact that 683 protein fold superfamilies are shared by eubacteria and archaebacteria, whereas only nine are unique to archaebacteria [119] further emphasizes that rRNA trees grossly exaggerate their molecular divergence. The neomuran revolution clearly took place in a highly developed cell with well over a thousand proteins and advanced membrane biology, including isoprenoid lipid carriers for surface carbohydrate secretion and fully developed prokaryotic division machinery. As Peretó et al. [18] also convincingly argued, the transitional organism was not a progenote without lipid membranes.

Three separate polarizations within the tree (the derived nature of archaebacterial reverse gyrase [1]; the neomuran revolution, discussed previously in detail [1]; the distinctly earlier origin of proteasomes from HslV) together establish that archaebacteria were derived from actinobacteria-like posibacteria (most likely specifically from actinomycete relatives) and thus are the most recent unibacterial phylum. Three further polarizations (the negibacteria to posibacteria transition, the glidobacterial ancestry of flagella, and the still earlier origin of Omp85) independently and together establish that unibacteria evolved from negibacteria. These six polarisations together show that archaebacteria are the youngest and chlorobacteria probably the oldest bacterial phylum. The conclusion that archaebacteria are the youngest bacterial phylum is immensely stronger than the conclusion that chlorobacteria are the oldest phylum as it stems from numerous independent polarisations that would be very hard to overturn, not just one.

I now present a new argument that for the first time locates the negibacterial root, based on the recent discovery that Omp85 homologues are essential for inserting β-barrel proteins into the outer membrane of proteobacteria, mitochondria, and chloroplasts [120].

### Chlorobacteria have the simplest outer membranes

Chlorobacteria [1] comprise filamentous 'non-sulphur' green bacteria, e.g. *Chloroflexus*, *Oscillochloris*, *Chloronema *and *Heliothrix*, which can be photoheterotrophs or photoautotrophs; colourless heterotrophs, including thermophiles (e.g. *Thermomicrobium*, *Herpetosiphon*); and chlororespirers (*Dehalococcoides*). Given the importance of Omp85 for insertion of all OM β-barrel proteins, it is striking that my BLAST analysis reveals no clear homologues in the complete *Dehalococcoides *[121] and *Chloroflexus *genomes , even though Omp85 is highly conserved and readily detectable in all other negibacteria including the hadobacteria *Deinococcus *and *Thermus *(e.g. Omp85 of the primitive cyanobacterium *Gloeobacter *has no BLASTP hits at all with Chlorobacteria but strongly hits sequences from every other negibacterial phylum; Omp85 of the sphingobacterium *Bacteroides *hits no Chlorobacteria but strongly hits other negibacterial phyla; Omp85 of the deltabacterium *Geobacter *hits all negibacterial phyla but Chlorobacteria. Moreover only five putative OM proteins have so far been detected in *Chloroflexus *(fewer in *Dehalococcoides*), several times fewer than in other negibacteria, including Hadobacteria, and do not include obvious homologues of typical OM β-barrel proteins. It is very unlikely that Omp85 homologues were lost by Chlorobacteria, as they have been retained for OM protein targeting even in the highly modified mitochondria and chloroplasts, descendants of anciently enslaved negibacteria, and their loss by both *Escherichia coli *and yeast is lethal [120]. I therefore argue that their absence is strong evidence that Chlorobacteria are the most primitive negibacteria of all and that they diverged from all other negibacteria before Omp85-based protein targeting evolved. This is consistent with their being the most divergent of all photosynthetic eubacteria on rRNA trees.

As advanced negibacteria (glycobacteria) have only β-barrel proteins in their outer membrane (also one α-,β-barrel) [122], determining what proteins Chlorobacteria use as outer membrane pores and their targeting machinery should throw considerable light on the origin of the first negibacterium. Since β-barrel proteins are thermodynamically self-inserting above 30C [122], and Chlorobacteria are either thermophiles or grow remarkably slowly, they may manage, as the first negibacterium must have done [31], without help from such strong catalysts of insertion as Omp85. If Chlorobacteria all lack Omp85 homologues and they were not lost, the root of the negibacterial part of the universal tree must lie either between them and all other negibacteria or within Chlorobacteria. This argument helps locate the universal root by excluding it from or among any of the negibacterial phyla other than Chlorobacteria. Thus the root cannot be within proteates, posibacteria (or between them and negibacteria) or the Omp85-containing negibacterial phyla. The only remaining possibilities are within either Chlorobacteria or between them and all other organisms. Which of these is true must be determined by future transition analyses using other characters not considered here.

It is possible that Chlorobacteria do not entirely lack Omp85 homologues, but have a relative too divergent for detection by simple BLAST. This possibility is seriously raised by the fact that the mitochondrial homologue Sam50 is not detected by all prokaryote BLAST queries; moreover, using the *Schizosaccharomyces pombe *sequence as BLAST query readily detects bacterial homologues, but only within Gracilicutes, with the highest hits all from α-proteobacteria as expected. The relatively high e-values of ~10^-13 ^for its hits with plants indicates that Sam50 has a markedly elevated evolutionary rate compared with negibacterial or chloroplast Omp85 homologues, which may be because its function changed greatly with the evolution of mitochondrial protein import and interaction with novel proteins. However as Chlorobacteria are exclusively free-living, not enslaved endosymbionts, there is no particular reason to expect such an elevated rate of evolution for them; if they do have very divergent Omp85s, this is likely to reflect either a very early divergence and/or a much less constrained pattern of interactions with other proteins than in any other negibacteria. If that were to prove the case, it would support rather than contradict the thesis that they are the most primitive negibacteria in terms of OM structure. Indeed the presence of such a highly divergent Omp85 would rule out any possible objection to my conclusion on the grounds that it might have been lost from Chlorobacteria, but without such loss being as harmful as I would expect it to be.

The apparent absence of a full sized homologue in *Thermotoga *is perhaps not surprising, as there has long been doubt whether it is a negibacterium or not [1], as its toga differs so greatly from a typical OM, both in chemical composition and in not being closely attached to the cytoplasmic membrane via a peptidoglycan sacculus. Although it has sometimes been classified as a posibacterium, I argued above that *Thermotoga *flagella structure shows that it is a negibacterium and that other evidence suggests that its frequent grouping on sequence trees with the also hyperthermophilic *Aquifex*, may be an artefact and that it is really related to Selenobacteria. Its novel togal morphology probably entailed drastic changes in OM morphogenesis when it originated, so Omp85 itself may then have changed equally dramatically (assuming that the small 403-amino-acid protein, AAD36794, detected by BLAST with a very weak e-value of 1.6, is derived from it). *Aquifex*, which often groups with *Thermotoga *on sequence trees, has a far better conserved full length Omp-homologue (NP_213890) with highest hits to deltabacteria (although I classified it in Epsilobacteria [1]). As, by contrast, the chlorobacterial OM is morphologically normal, there is no reason to invoke an analogous drastic secondary change in their OM morphogenesis to explain the apparent absence of Omp85 from Chlorobacteria (both BLAST and PSI-BLAST were used to search in Chlorobacteria).

In all bacterial phyla except Chlorobacteria BLAST revealed homologues of the FtsX-domain-containing cytoplasmic membrane proteins LolC and LolE, which suggests that the OM lipoprotein transport machinery also of chlorobacteria may be more primitive than in any other bacteria. In proteobacteria of subphylum Rhodobacteria (purple bacteria) these associate with an ABC family membrane ATPase LolD that provides energy for release of OM lipoproteins from the cytoplasmic membrane into the periplasm. There they bind to a chaperone LolA, which releases them to a related OM lipoprotein LolB [123]that places them in the OM. It is unclear whether LolA and LolB are present outside Rhodobacteria as they evolve too rapidly for detection by BLAST over large evolutionary distances. Either LolC and E originated only after Chlorobacteria diverged (Fig. 7) or they have too divergent homologues to detect. Like all other bacteria they have plenty of ABC ATPases, one of which could have been recruited later to help with lipoprotein release.

### Negibacterial monophyly and lipopolysaccharide evolution

Six negibacterial phyla have lipopolysaccharide in the outer leaflet of their outer membrane. As its synthesis is so complex [59], it could not have evolved twice independently or be transmitted by lateral gene transfer. Therefore the monophyly of these six phyla (known collectively as glycobacteria [1], i.e. all negibacteria except Chlorobacteria and Hadobacteria) has long been clear [30] (though often overlooked). My finding Omp85 in Hadobacteria (by BLAST) indicates that glycobacteria plus Hadobacteria all had a common ancestor that was the first to evolve Omp85. On rRNA trees Chlorobacteria are deeply divergent from other negibacteria, but do not group with either of the two posibacterial subphyla (Actinobacteria or Endobacteria) or with neomura. Therefore there is no reason to doubt that the eight negibacterial phyla are collectively monophyletic despite the absence of a well-conserved Omp85 from Chlorobacteria. The arguments presented above clearly establish that negibacteria are not the holophyletic descendants of posibacteria, as Gupta assumed [79], but their paraphyletic ancestors as Blobel [57] suggested and I explained in detail [1,29,30].

With a negibacterial root to eubacteria, Omp85 would inevitably have been lost when posibacteria lost the outer membrane, which would have been entirely harmless because its essential function of inserting β-barrel proteins would simply have gone. I postulated that was its only loss in the history of life [1,5,29,30]. The greater simplicity of the chlorobacterial OM, with neither lipopolysaccharides nor apparently an Omp85 protein-targeting mechanism, is what one expects of the earliest negibacteria, and is unlikely to have been caused by secondary loss of Omp85 alone while retaining an OM; there is no evidence that such loss has ever occurred in the whole history of life. Although the unique absence of a well-conserved homologue of Omp85 from the OM of Chlorobacteria is strong evidence that they are the most ancient negibacteria, it does not by itself prove that they are the most ancient eubacteria of all. To do that we must also exclude the root from all Posibacteria; proteasome evolution excludes it only from Actinomycetales and neomura, and thus left open the possibility that it might be beside or within Endobacteria (or even among early actinobacteria). As argued above, negibacterial envelope biogenesis is so complex as to polarise the transition from negibacteria, not the reverse, and thus exclude the root from all unibacteria. My new arguments concerning flagella evolution also clearly place the root within negibacteria, specifically among Glidobacteria. When discussing the origin of flagella I pointed out that the root cannot lie within Spirochaetae, or within Sphingobacteria, which have a unique form of gliding motility not related to flagella. I now give further evidence that, although not as compelling as the polarizations discussed above, nonetheless also indicates that Gracilicutes are a major derived group within Negibacteria.

### Gracilicutes are probably a clade

Three negibacterial phyla that share a rare 4-amino acid insertion in alanyl-tRNA synthetase [1,81] together correspond closely to the classical division Gracilicutes, so I designate them 'core gracilicutes' (Proteobacteria, Sphingobacteria, Planctobacteria). They also all share a β-β' module domain 1 (BBM1) inserted into the universally conserved second sandwich barrel hybrid motif (SBHM) domain in the RNA polymerase β-subunit [82]. Since SBHM and BBM1 domains are both well conserved and present at different locations in various proteins, they were somewhat genetically mobile during early evolution. As the BBM1 domain interrupts the SBHM domain it was inserted after the β-subunit of RNA polymerase first evolved and is thus a derived state compared with genes lacking that insertion. Unless this insertion has been reversed since the gene originated in some or all other bacteria, this excludes the root of the tree from core gracilicutes. Two shared single-amino-acid insertions in the highly conserved bacterial division protein FtsZ and chaperonin Hsp60 suggested that core gracilicutes are sisters of spirochaetes [1,81]. This is now supported by a very large alpha-helical insertion in RNA polymerase D/σ^70 ^in Proteobacteria, Planctobacteria, and Spirochaetae [82]; Sphingobacteria, uniquely among eubacteria, have a large N-terminal deletion of this molecule, so the absence of the α-helical insertion in Sphingobacteria might also reflect a secondary deletion, possibly caused by the same shift in function associated with the first. I have here resurrected the name Gracilicutes, discontinued as a phylum name, slightly more broadly to include Spirochaetae also, which are evidently more closely related to core gracilicutes than to other eubacteria. Gracilicutes are weakly holophyletic on a Bayesian concatenated 16S/23S rRNA tree [80] (except for the probably incorrect exclusion of *Aquifex*) and strongly holophyletic on the concatenated ribosomal protein and two of the four RNA polymerase subunit trees of [82]. All four gracilicute phyla also share a β-β' module 1 domain inserted into the β' subunit of RNA polymerase.

Probably these insertions were complexities added in the gracilicute common ancestor to improve transcription or its control. Proteobacteria include many of the fastest growing, most phenotypically adaptable bacteria – for which rapid transcription and its efficient switching is important. Possibly these RNA polymerase insertions helped them enter this adaptive zone. These insertions polarize change from the simpler eobacterial RNA polymerase to the more complex gracilicute polymerase. If correct, this excludes the universal root from all Gracilicutes. In my view this argument, though reasonable, is not as compelling as the Omp85, cell wall, and flagellar arguments for polarising the tree, because the two gracilicute-wide RNA polymerase insertions and the FtsZ and Hsp90 insertions (Fig. 7) might have been argued to have been deletions instead if other evidence had suggested that the universal root was within gracilicutes. Even the evidence from the core gracilicute insertion is less strong than for Omp85, cell walls, and flagella, where there are strong reasons for thinking that evolution could not have gone in the reverse direction.

The main importance of these six indels, apart from their congruence with the rooting based on Omp85, is that they partition bacteria into two large groups, one derived and one ancestral: the gracilicutes and the eobacteria/posibacteria. Other evidence (Omp85) is stronger for the gracilicutes being derived and holophyletic and eobacteria/posibacteria paraphyletic. In eukaryotes analogous molecular cladistic characters have been of very great value in partitioning major taxa into supergroups, especially where gene sequence trees have lacked sufficient resolution to do so or have given positively misleading answers because of systematic bias; the most striking examples are the recent partitioning of eukaryotes into unikonts and bikonts by a gene fusion and myosin synapomorphies (Fig. 7) [3,4,28] and the very strong support from gene replacements for the chromalveolates [124,125]. Such characters are at least as important in the much older eubacteria where gene trees are usually still less well resolved at the base, probably through a combination of substitutional saturation and rapid early radiation of all eubacterial phyla.

Importantly, the insertions place all Posibacteria in the same category: Actinobacteria and Endobacteria both lack all five. If they were polyphyletic any insertion could have placed them in different clades. The hyperthermophilic eubacterium *Aquifex *has all of the gracilicute-specific insertions [82]. Several authors have reasonably argued that the degree of separation of the *Aquifex*/*Thermotoga *branch from other eubacteria on rRNA trees and some protein trees may be an artefact of their shared hyperthermophily, which so strongly biases base composition towards GC as to be likely to bias amino acid sequences also. *Aquifex *clearly groups with Proteobacteria with some of the most conserved proteins (e.g. cytochromes b and c [126], phosphoglycerate kinase [127]); I formally classified them thus [1]. *Aquifex *has both Omp85 and the Skp chaperone for insertion of OM proteins, like Proteobacteria and all Gracilicutes. Moreover there is an *Aquifex*/Proteobacteria-specific insert in RNA polymerase β, and on the trees for the two largest subunits (therefore most reliable) *Aquifex *was robustly within Proteobacteria as sister to ε-Proteobacteria [82], where it is classified [1], which is fully congruent with the flagellar evidence discussed above. Thus cladistic molecular characters agree with cladistic morphological characters and significant protein trees and disagree with the probably systematically biased rRNA tree [1]. Assuming instead that the rRNA tree is correct, and postulating lateral transfer of RNA polymerase genes from proteobacteria [82], was unwarranted.

*Thermotoga *is more problematic. It lacks both gracilicute RNA polymerase insertions, the gracilicute Hsp60 insertion, and the core gracilicute RNA polymerase insertion, while its Omp85 homologue is unusually small, and Skp (a chaperone otherwise found in all negibacteria except Chlorobacteria and Cyanobacteria: see next section) is not detectable. *Thermotoga *probably lacks the gracilicute FtsZ insertion, but its FtsZ alignment in the region is slightly ambiguous, so one cannot be sure that precisely the same amino acid is missing from *Thermotoga *as in Posibacteria, though it may be; moreover, a dozen residues downstream, sphingobacterial FtsZ has suffered a two-amino-acid deletion, showing that a nearby region of the molecule in gracilicutes is not totally immune to secondary deletion. As each of these five independent insertions could in principle have been secondarily deleted (or lost by gene conversion from one of its many laterally transferred foreign genes), they are not totally decisive evidence against placing Thermotogales with *Aquifex *in Thiobacteria. Yet they are strong reasons for not doing so lightly, i.e. just because of potentially biased sequence trees grouping *Thermotoga *and *Aquifex*. Although *Thermotoga *shares a sizeable deletion in Hsp70 with Posibacteria, it does not actually branch with them on the tree so this also might be convergent. A small RNA polymerase D domain deletion [82] also links *Thermotoga *and Posibacteria, but might be convergent. It is important to note that the evidence from indels is asymmetric. A highly conserved insertion, e.g. that in the alanyl-tRNA synthetase of core gracilicutes or the RNA polymerases domains is evidence of a common ancestry, but the absence of an insertion is more ambiguous as species without it may be a mixture of those that never had it and diverged earlier and those that underwent a secondary deletion at the same place. Even though comparative studies show that partial gene deletions have been rampant in *Thermotoga*, the fact that all seven indels mentioned are congruent in excluding it from the Gracilicutes and grouping it instead with Eurybacteria gives them collectively much more force than if each was considered on its own. As *Thermotoga *has lost Hsp90 we cannot use its deletion (Fig. 7) to place it, but the glycerol-1-P dehydrogenase tree and the very presence of that unusual enzyme [18] clearly support its being a sister to Posibacteria. Genome sequences for Selenobacteria, the phenotypically least derived Eurybacteria are badly needed to test its current placement in Eurybacteria (Table 1). Studies of toga biogenesis are also greatly needed, both to test that it really is a highly modified negibacterial OM and to understand how and why that modification occurred.

### The glycobacterial revolution

The monophyly of the exclusively non-photosynthetic Hadobacteria (often informally, but clumsily known as the *Deinococcus*-*Thermus *group) is generally accepted but there is no consensus on their precise evolutionary position. Chlorobacteria and Hadobacteria sometimes group weakly together on rRNA trees, but often do not. As they have ornithine not diaminopimelic acid in their murein cell wall polymer and are the only negibacteria lacking lipopolysaccharide or lipo-oligosaccharide in their outer membrane, I grouped them as phylum and infrakingdom Eobacteria [1]. Eo-('dawn') emphasised that lipopolysaccharide (LPS) absence may be primitive and they could be the earliest cells. Although I still think that both phyla primitively lack LPS and diaminopimelic acid, I now consider Chlorobacteria as markedly more primitive than Hadobacteria and that Eobacteria are paraphyletic, not holophyletic [1]. Given also the large number of differences between Chlorobacteria and Hadobacteria summarised in Fig. 7 it is now sensible to treat them as separate phyla, rather than subphyla, within a superphylum Eobacteria (Table 1), which I now group with Cyanobacteria as the new infrakingdom Glidobacteria: ancestrally gliding bacterial invariably without flagella, i.e. all the primitively non-flagellate negibacteria. LPS is typically composed of the very complex lipid A, which anchors it in the OM outer leaflet, a core oligosaccharide and an external O-antigen, which is typically a long polysaccharide but has been reduced to an oligosaccharide in spirochaetes, or sometimes lost as in *Neisseria*. Lipid A is variable in structure but typically has a glucosamine disaccharide backbone with about six acyl ester hydrocarbon tails. The very great complexity of LPS makes it implausible as a primitive state for negibacteria, as does the complex, only partially characterized, machinery for exporting its precursors made in the inner leaflet of the cytoplasmic membrane across that membrane and mature molecules across the periplasm to the OM [60]. I shall argue that it can only be lost by glycobacteria if its impermeabilizing function is replaced by another complex lipid.

Placing both Chlorobacteria and Hadobacteria at the base of the tree would have been incorrect if either had lost LPS. It has long been known that LPS was lost when a proteobacterium was converted to the first mitochondrion and when a cyanobacterium was converted to the first chloroplast. In both cases host phosphatidylcholine replaced it in the outer leaflet of the bilayer [105]. It is also obvious that LPS was lost together with the OM when the first posibacterium evolved from a eurybacterium, as explained above. The fact that loss of LPS is exceedingly rare among negibacteria that have retained an OM and never been enslaved and converted into eukaryotic organelles means that it possesses a really fundamental function that is almost universally required and not readily replaced. I know of only two groups of glycobacteria that have lost LPS: spirochaetes and the α-proteobacterium *Sphingomonas*. These natural experiments clearly reveal LPS function to be making a very rigid non-fluid outer surface that is impermeable to small foreign hydrophobic molecules that might disrupt cytoplasmic membrane functions [59].

Spirochaetes either modified LPS (*Leptospira *[58]) or lost it (others). I suggest that this was done to increase the flexibility of the OM as required for their corkscrewing motility by endoflagella; the obligate parasites that lost it may need less protection from foreign hydrophobic permeants within their animal hosts. There is no risk in confusing their condition with the primitive absence of LPS in Chlorobacteria and Hadobacteria, which presumably have phospholipids in the outer leaflet of their OM as in the cytoplasmic membrane. There is other compelling evidence that spirochaetes are morphologically the most derived, not the most primitive negibacteria. The spirochaete state is not a primitive or a simple state; it would be unreasonable to suppose that spirochaetes without LPS are the most primitive bacteria as all can only grow inside animals.

*Sphingomonas *did not abandon the protective role of LPS when it lost it. It was only able to lose LPS by replacing it with a complex glycosphingolipid (D-glucuronosylceramide), found also in about six related genera of α-proteobacteria, and with precisely the same protective properties [59]. Sphingolipids, however, are somewhat less rigid than LPS, which may be why it was replaced. Sphingobacteria were thus named because many of them supplemented rather than replaced their LPS by sphingolipids, best studied in Flavobacteria, e.g. *Bacteroides*, *Porphyromonas*, *Sphingobacterium*. As they use chemically different types of sphingolipids (the sugarless ceramides or sphingophospholipids) from *Sphingomonas *[59], this supplementation probably evolved independently of the replacement of LPS in *Sphingomonas*, though no doubt sphingosine itself arose in the common ancestor of Proteobacteria and Sphingobacteria. The *Cytophaga*-*Flexibacter *group of Sphingobacteria partially replaced their LPS instead by the chemically somewhat related sulphonolipids rather than sphingolipids. It was proposed that these Flavobacteria supplemented their LPS by sulfonolipids to make the OM less rigid and thus facilitate their novel gliding mechanism [59]. The extreme halophile *Salinibacter*, which is related to them but unusually has flagella [95], also has sulfonolipids [128].*Flavobacterium johnsoniae *appears to use both sulphonolipids and ornithine lipids for this purpose [59]. It is known that beads attached to flavobacterial surfaces are propelled along the OM; possibly the motors doing this require a fluid rather than rigid outer leaflet of the OM. I suggest that the origin of lipoprotein-based gliding motility took place in the sphingobacterial cenancestor prior to the widespread loss of flagella and that the addition of the sphingolipids, sulfonolipids and/or ornithine lipids to the OM were secondary adaptations for fast gliding motility that probably occurred somewhat later, after the divergence of Chlorobea and Flavobacteria and the ancestor of Flavobacteria lost chlorosomes with their unique lipid monolayer structure. Phylogenetic studies of sphingobacterial lipid-synthesising enzymes are needed to test this. Thus the unusual lipid composition of most, if not all, Sphingobacteria is probably coadaptive with their gliding motility and not arbitrarily coincidental, supporting the view that coadaptation among disparate cell properties is often a key to understanding cell megaevolution [29,129], which single character or single molecule studies (e.g. 16S rRNA) necessarily fail to illuminate.

I now suggest that the evolution of LPS was not a trivial matter, but of immense adaptive significance for negibacterial evolution, as it also necessarily entailed the origin of numerous OM porins, whose origin was itself coadaptive with the origin of LPS. The hollow cylindrical β-barrel porins are absolutely essential for glycobacteria (here simply treated as a monophyletic grade, not a formal taxon as previously [1]), but less so for Eobacteria. Porins have two major functions. First, to allow the passive entry or active import of nutrients that cannot diffuse through the impermeable LPS outer leaflet of the OM or which do so too slowly for the cell's growth requirements. Second, to actively expel harmful foreign molecules that still seep in slowly despite the high degree of impermeability of the OM. Thus the evolution of active expulsion mechanisms and of greater impermeability by evolving LPS are both part of the same suite of adaptations that occurred during the transition from Eobacteria to glycobacteria. I now call this the glycobacterial revolution, and argue that it probably involved numerous dramatic and essentially simultaneous innovations in OM cell biology.

Two key innovations seem to have been the origin of the TonB complex, important for subsequent evolution of flagella, as discussed above, and the origin of type I secretion by means of TolC. TolC is a remarkably long (14 nm) hollow cylinder that penetrates the OM but is attached at its base to an ABC transporter embedded in the cytoplasmic membrane, and which pumps secretory products into the TolC lumen from the cytosol [130]. The tube is formed by three TolC proteins; TolC differs fundamentally from other membrane proteins in being neither a β-barrel (typical OM proteins of glycobacteria) or simple α-helical proteins (cytoplasmic membrane proteins) but is a unique α-β-barrel. Its periplasmic part is an α-helical tunnel, which is covalently contiguous with its β-barrel channel embedded in the OM. TolC is open to the outside medium but closed by an iris-like mechanism at its periplasmic entrance. When it binds to the ABC transporter laden with substrate, the α-helical iris opens allowing the substrate to pass out through a tube spanning the entire cell envelope, from the cytosol to the exterior [130]. TolC acts to expel harmful organic molecules made by other organisms, and probably would not have been important until after chemical warfare by secondary metabolites among inhabitants of microbial mats became an unavoidable fact of life. TolC seems likely to have evolved by gene fusion between an α-barrel protein and a pre-existing β-barrel protein, and thus for that reason also can hardly have been present in the most primitive negibacteria.

As secretion of TolC into the periplasm/OM itself depends on Omp85 [131,132], it must have evolved after Omp85. In keeping with this, TolC is absent from Eobacteria but present in all glycobacteria (but unsurprisingly no unibacteria). Its assembly does not require the presence of LPS in the OM, so it might have evolved prior to LPS. In *Escherichia *other OM proteins (e.g. OmpF/C) appear to require LPS for their correct assembly and thus might be argued to have originated after LPS evolved, but dependence on LPS is not universal as *Neisseria *can dispense with it at least in the laboratory [60], weakening such an interpretation. Other OM assembly mechanisms, e.g. requirement for the periplasmic chaperone SurA, itself insertable into the OM (detectable homologues only in Proteobacteria with some very distant hits in Sphingobacteria), may have evolved even later. Omp85 is essential also for these, as expected if it evolved prior to all glycobacteria as shown on Figs 3, 7.

The complexity of the import and transducing mechanisms mediated by TonB, and the fact that like Type I secretion it depends on a complex cooperation of dissimilar cytoplasmic and OM proteins, is also such that they can hardly have been present in the first negibacterium just after its OM had differentiated from the cytoplasmic membrane during the formation of the ancestral cell [31]. Absence of both TolC and TonB as well as the very complex lipopolysaccharide are all to be expected from the most primitive negibacteria, which further supports the rooting within eobacteria. A major research programme is needed into OM biogenesis and physiology in Eobacteria to see whether it is really substantially more primitive than that of glycobacteria, as I have argued. Reviews on bacterial OMs often assert that many of the proteins that I have been unable to find in Eobacteria are present in 'all' Negibacteria; as many biologists whose main experience is on medically important bacteria or models like *E. coli *also falsely assume that all negibacteria have LPS, this mistake is not surprising. Even more proteins widely assumed to be general for negibacteria are probably missing from Chlorobacteria, but present in Hadobacteria. These probably evolved after Chlorobacteria, but prior to the divergence between Hadobacteria and Cyanobacteria, which I must now discuss.

### Photosystem duplication, catalase, and the great oxygenation event

The absence of Omp85 in Chlorobacteria strongly argues that they preceded Hadobacteria. Eleven other synapomorphies shared by Hadobacteria and most eubacteria (Fig. 7), but absent from Chlorobacteria, support the conclusion from Omp85 that Hadobacteria are less primitive than Chlorobacteria and arose later. These include the HslVU protease discussed above (only in *Thermus*), a small chaperone protein (Skp) important in preventing aggregation of OM proteins in the periplasmic space [60], and monofunctional catalases belonging to clades 1 and 2 [133]. These haem catalases are not detectable by BLAST in *Thermus*, which seems only to have manganese catalases, so it is likely that they were lost or altered beyond recognition by *Thermus *when it became a thermophile.

One might expect catalases and other hydroperoxidases to be absent or less diverse before the atmosphere became oxygenic. Hydroperoxidases, of five major types, destroy the poison hydrogen peroxide generated by active oxygen. As oxygen concentrations were low when life began, Klotz and Loewen [133] suggested that the first to evolve were rubredoxin peroxidases, which become saturated at low (micromolar) peroxide concentrations, a limitation not then a drawback. Rubredoxin peroxidases have a 1,000-fold higher specificity for H_2_O_2 _than the other four types (catalases) and occur in all major groups of organisms, including Chlorobacteria and cyanobacteria. I suggest that the unrelated catalases evolved only when oxygenic photosynthesis evolved and vastly increased oxygen levels. Their advantages, despite lower specificity, are much higher turnover rates and saturation levels that could cope with greatly increased levels of peroxide after the early Proterozoic 'great oxygenation event' caused by cyanobacteria and their immediate ancestors [134]. Monofunctional catalases probably evolved soon after oxygenic photosynthesis. They would have originated in the common ancestor of Hadobacteria and Cyanobacteria: all organisms except Chlorobacteria (Figs 3, 7).

Some readers may wonder how I can argue that catalases originated in the common ancestor of Hadobacteria and Cyanobacteria as a response to oxygenic photosynthesis, if Hadobacteria diverged before Cyanobacteria. It is often loosely stated that cyanobacteria invented oxygenic photosynthesis. However this need not be true, and is probably strictly incorrect. There is good evidence from many sources (e.g. [135]) that extant cyanobacteria are holophyletic. Therefore both phycobilisomes and oxygenic photosynthesis must have been already present in the cenancestor and must have evolved earlier still. If phycobilisomes evolved after oxygenic photosynthesis (Fig. 7), the first organism that generated oxygen was not actually a cyanobacterium but an evolutionary precursor. Since the haem oxygenases that split the porphyrin ring to make phycobilins require oxygen, they and phycobilisomes probably both evolved after oxygenic photosynthesis. Haem oxygenases are unknown in eobacteria and probably first evolved during the glycobacterial revolution.

However a few other oxygenases are found in *Chloroflexus*, which can live both anaerobically and aerobically. If Chlorobacteria are indeed the earliest diverging bacteria and diverged before oxygenic photosynthesis and two photosystems evolved, as argued here, they must have been ancestrally anaerobic. I therefore predict that phylogenetic analysis of all oxygen-requiring enzymes in the phylum will show that all arose secondarily, either by modification of ancestrally oxygen-independent enzymes or by lateral gene transfer from more advanced phyla. *Chloroflexus *makes structurally different enzymes for some steps in haem and bacteriochlorophyll synthesis [136]; as one enzyme functions under anoxic conditions and the other performs the same reaction in oxic conditions, this dichotomy might reflect a later recruitment of the oxic enzymes. Lateral gene transfer is the likely explanation of the presence of the intradiol ring cleavage dioxygenase of *Chloroflexus *and *Deinococcus*; they are not detectable in other eobacterial genomes and are too similar to those of actinobacteria for vertical inheritance to be believable, though phylogenetic analysis is needed to confirm this. The 4-hydroxyphenylpyruvate dioxygenase, involved in aerobic tyrosine degradation, of *Chloroflexus *is even more similar to those of actinobacteria, making lateral transfer very likely.

**Figure 9:**
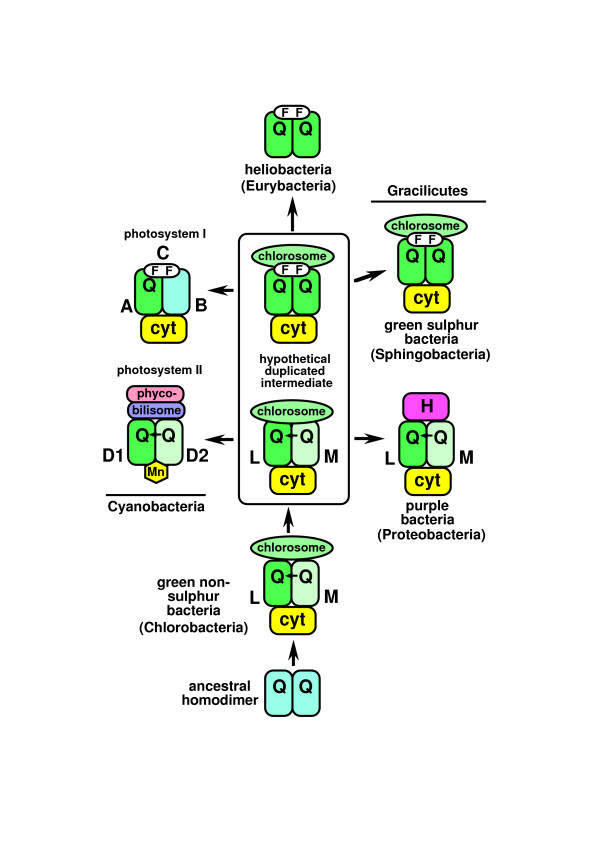
Hypothetical phylogeny for photosynthetic reaction centres. Prior to the last common ancestor of all extant life the primitive reaction centre, a homodimer with two bound quinones, each donating electrons to a primitive cytochrome cc complex, evolved into the heterodimeric type found in green non-sulphur bacteria (Chlorobacteria). This was duplicated prior to divergence of cyanobacteria and gracilicutes to generate a modified homodimeric type of cytochrome bc_1 _complex with iron-sulphur clusters (FF); for a mechanistic explanation of this duplication see [126]. Cyanobacteria converted the two versions into photosystems I and II. Proteobacteria replaced chlorosomes in the original heterodimeric type by an H subunit with purple carotenoid, but did not retain the new duplicate with FeS clusters. By contrast, this was the only version retained by green sulphur bacteria (Sphingobacteria) and Heliobacteria, both losing the earlier heterodimeric type. This scenario is simplified from ref. 1 and congruent with the cladistic tree in Fig. 7 and the concatenated rRNA tree [80] and is compatible with photosynthetic protein trees, if properly rooted (see text).

It is generally accepted that photosystem I and photosystem II, which carries the oxygenic reaction centre, arose by duplication and divergence from a common ancestor that had only a single photosystem. Just when in the tree did this duplication occur? Clues come from the fact that the two partly photosynthetic gracilicute phyla (Proteobacteria, Sphingobacteria) have very different photosystems. As Fig. 9 indicates, that of green sulphur bacteria such as *Chlorobium *resembles photosystem I of cyanobacteria, whereas that of purple bacteria is more like photosystem II of cyanobacteria. Given the root of the tree in or beside Chlorobacteria, it is entirely unnecessary to postulate lateral gene transfer of complete photosystems as is sometimes unparsimoniously done. Vertical inheritance alone can very simply explain the known diversity of photosystems and their distribution among phyla given the root and topology of the eubacterial tree as now deduced by cladistic and transition analysis (Figs 3 and 7).

As Gracilicutes are almost certainly holophyletic, their common ancestor probably still retained both photosystems, which must have duplicated prior to the somewhat older common ancestor that it shared with cyanobacteria. Photosystem II was lost by the ancestor of Sphingobacteria only after it diverged from the ancestor of Proteobacteria, which retained it but replaced the ancestral chlorosome pigments by new purple pigments to allow spectral niche differentiation; they lost photosystem I instead. The flagellate, and probably ancestrally facultatively aerobic, purple bacteria could colonise the open water above the cyanobacterial benthic mats, while the non-flagellate green sulphur bacteria could glide down below them to exploit light frequencies that filtered through. They lost photosystem II, as did the ancestors of Eurybacteria from which Heliobacteria evolved. The common ancestor of Gracilicutes and Eurybacteria must still have had chlorosomes, if we reject lateral gene transfer of entire photosystems and antenna complexes as unparsimonious and less likely than simple vertical inheritance. Thus its earlier ancestor that gave rise to cyanobacteria also had chlorosomes too; phycobilisomes would have evolved only after the ancestor of cyanobacteria diverged from it (Fig. 7), which is in keeping with the other evidence that cyanobacteria are holophyletic. On this simple interpretation involving only gene duplication and divergence, the hypothetical duplicated intermediate shown in the box in Fig. 9 would have had only a transient evolutionary existence if cyanobacteria, purple bacteria, Chlorobiales and heliobacteria all diverged in a single bout of photosynthetic niche differentiation immediately following the glycobacterial revolution. Thus on this interpretation it was a green eobacterium that invented oxygenic photosynthesis, not a cyanobacterium. Since Hadobacteria are non-photosynthetic, their ancestor must have lost photosynthesis after it diverged from the chlorosome-containing ancestor of cyanobacteria. Such a transitional intermediate between Chlorobacteria and Cyanobacteria was also postulated by the redox switch hypothesis [137], an ingenious and plausible mechanistic explanation for the photosystem duplication assumed by Fig. 9 that requires no lateral gene transfer; this intermediate would be the first organism to have generated oxygen internally and thus need catalase. Lateral transfer is also not needed to explain evolution of cyanobacterial core antenna domains [138].

Cladistic arguments do not tell us whether the photosystem duplicated before or after the divergence of hadobacteria. However the fact that catalases apparently first evolved in that common ancestor suggests that it itself had already evolved oxygenic photosynthesis and evolved catalases to protect itself from damage from its own oxidising excreta. Thus Hadobacteria evolved by losing both the freshly duplicated photosystems and specialising on heterotrophy. The exceptional radiation resistance of *Deinococcus *could date back to the time when its relatives that evolved oxygenic photosynthesis had not yet oxygenated the atmosphere sufficiently to provide an ozone layer protective against UV. If, as suggested above, the radiation of glycobacterial photosynthesizers was very rapid, the new duplicate photosystem that evolved prior to their last common ancestor could have retained its originally homodimeric character until the Heliobacteria and Sphingobacteria split off from the common stem, becoming fully differentiated as a heterodimeric reaction centre only in the single ancestral cyanobacterial lineage (Fig. 9). This appears to be a simpler interpretation of photosynthetic diversification than almost any others previously published, and one that is fully consistent with the evolution of the rest of the cell, and also reasonably so with most sequence trees, provided that they are correctly rooted.

The above interpretation places the origin and primary radiation of glycobacteria immediately after the origin of oxygenic photosynthesis; only Hadobacteria diverged earlier, though possibly as little as a few thousand years before the glycobacterial revolution. I suggest that the vast expansion of a photosynthetically active population that used water rather than hydrogen or hydrogen sulphide as the source of reductant for carbon dioxide fixation and the hugely greater supply of carbohydrate now available was what stimulated the evolution of the much more complex OM of glycobacteria. Higher population densities would have yielded far more harmful products of death and decay and far more biocidal small molecules made by the more elaborate secondary metabolism that would then have become possible and likely through more intensive interference competition, against which LPS would have been protective and also provide the lavish supply of carbohydrate needed to make it. Higher population densities would stimulate such chemical warfare and the struggle for resources. On this interpretation only Chlorobacteria are relics of the days before the origin of oxygenic photosynthesis and rich supplies of carbohydrate. Oxygen itself could have led to there being a greater variety of small harmful lipophilic molecules in the environment against which an LPS coat and active extrusion by TolC would be important complementary defences. This illustrates how the new chlorobacterial rooting gives new insights into the reasons for the structural diversity of negibacterial cell envelopes, and the distribution of protections against oxygen such as catalase. The intensive study of all aspects of chlorobacterial cell biology and ecology should have more than anything else to teach us about the nature of early life.

It was previously suggested [133] that the single case of a clade 3 catalase enzyme in cyanobacteria was the result of lateral transfer. However, phylogenetic evidence for that lateral transfer is unconvincing; many fewer lateral transfers and losses need be postulated with the present phylogeny than assumed earlier [133]. If clade 3 enzymes were ancestrally present in cyanobacteria, their origin on Fig. 7 must be moved below that of cyanobacteria and additional losses assumed; this small enzyme would then be the ancestral monofunctional catalase, which later underwent two gene duplications and divergence to generate another small (clade 1) and a larger (clade 2) paralogue, both present in *Deinococcus *and many Posibacteria and Proteobacteria (but not archaebacteria, apart from a probable lateral transfer of a clade 2 gene to *Methanosarcina mazei *[133]). If cyanobacteria ancestrally lacked clade 3 catalase [133], it probably originated later by duplication of a clade 1 gene after divergence of Hadobacteria, which lack it (Fig. 7); in either case a small version of the enzyme is likely the ancestral type, not, as previously suggested [133], the more complex clade 2 type, with extra end domains important only for quaternary structure. Clade 3 catalase was retained by neomura (including in opisthokont peroxisomes), except for green plants having the clade 1 type instead.

The catalase paralogue tree further exemplifies the poor resolution and conflicting results given by metabolic enzyme paralogue trees [1]: the clade A subtree places the root within eubacteria, specifically within Endobacteria [133]; if the A subtree is treated as unrooted it would have clear bipartitions between neomura and eubacteria, between eukaryotes/unibacteria (i.e. all organisms with a single surface membrane) and negibacteria (all with two), and between negibacteria and posibacteria – topologies entirely consistent with Fig. 7 (apart from the lateral transfer to *Methanosarcina mazei *[133]); but the long branch 'outgroup' (clade 1 and 2) incorrectly gives spurious midpoint rooting: moreover if the clade 3 enzyme is older, as suggested above, this assumed outgroup is actually an in-group and not expected to give the correct root, even if group 3 catalase existed in Chlorobacteria.

Mn catalase and catalase-peroxidase are more widespread in cyanobacteria [133] and probably evolved in the common ancestor of Cyanobacteria and all other organisms except Chlorobacteria.

### Implications for cytochrome evolution

It was recently discovered that chlorobacterial photosynthetic and respiratory electron transport chains contain remarkably different protein complexes from all other organisms and do not contain cytochrome b [139]. Given the new rooting of the tree of life by Omp85, this can be simply interpreted as the ancestral state for all cells, with cytochrome b evolving only later (Figs 3, 7) in the common ancestor of cyanobacteria and all other, more advanced, cells – another feature of the glycobacterial revolution. It was also found that a very well conserved operon, the MFIcc operon, is widely found in negibacteria from Chlorobacteria upwards on the tree of Figs 3, 7, but not in cyanobacteria or Unibacteria. This operon appears ancestrally to have comprised a multihaem cytochrome c (M), a MoCo subunit and an FeS subunit of a multidomain protein (F), an integral membrane protein (I), an uncharacterised protein, a 1-haem cytochrome c and another integral membrane protein. Cytochrome oxidase of type A2 shows a very similar distribution to this operon, being found in all negibacterial phyla (including Cyanobacteria) except Sphingobacteria and Eurybacteria, but in no known Unibacteria. A1 type cytochrome oxidases are present in Posibacteria and in the subphylum Rhodobacteria of Proteobacteria. The MFIcc operon was called 'recently "invented"' and it was asserted that organisms simultaneously possessing both the MFIcc operon and cytochrome oxidase A2 were unrelated, and widespread lateral transfer was assumed [139]. As no explicit reason was given for these very questionable suggestions, it is unclear how they might have been reached, but they are a likely example of incorrect conclusions derived from the widespread but incorrect assumption that the root of the tree lies outside negibacteria.

If those characters are mapped onto Figs 3 and 7 instead, a radically different and much simpler conclusion is apparent. The MFIcc operon is very ancient indeed, probably having been present in the cenancestor, but was lost independently by the ancestral cyanobacterium and unibacterium. In addition it was lost on several occasions within other phyla and secondarily truncated to an MFIc operon in Deltabacteria. A2 cytochrome oxidase is the ancestral state, and gene duplication in the common ancestor of Gracilicutes generated the A1 paralogue – the A2 version was then lost by Thiobacteria and by the ancestral posibacterium (or its common ancestor with eurybacteria; data are needed for eurybacteria to decide), and within Rhodobacteria. C and Q type oxidases may also have evolved at the same time and been differentially lost. Possibly no lateral transfers at all are needed.

At present the only chlorobacterium studied for electron transfer complexes is *Chloroflexus aurantiacum*, which has two c-type cytochromes (one single haem and one multihaem) and molybdopterin-related oxidoreductases in its respiratory and (different paralogues) in its photosynthetic electron transport chains. The photosynthetic versions are encoded within an MFIcc operon and most of its respiratory versions are in a second related but simplified operon with several genes missing [139]. Detailed studies of electron transport chains in numerous photosynthetic and non-photosynthetic chlorobacteria are needed to test whether cytochrome b is really entirely absent from all Chlorobacteria, as tentatively suggested here, and to understand these likely relics of the very early differentiation between respiratory and photosynthetic electron transport. Many surprising discoveries may be made.

### The chlorobacterial root of the universal tree

Figure 10 summarises the arguments that collectively point to the universal root being within eobacteria, specifically beside or within Chlorobacteria, not between archaebacteria and eubacteria as is widely supposed. Evidence for the evolution of proteasomes from HslV, not the reverse, excludes the roots from proteates (neomura plus actinomycetes – or a broader set of actinobacteria, depending precisely on when proteasomes originated). Since the archaebacterial/eubacterial boundary is within proteates, the universal root cannot be between archaebacteria and eubacteria (the 'standard model': [15,16,19]) or between eukaryotes and prokaryotes (as supposed by few [13]), or within eukaryotes or archaebacteria (both occasionally proposed [140]) but must be within eubacteria. The two negibacteria-posibacteria polarisations independently confirm this, one based on wall and one on flagellar evolution, whilst the Omp85 argument excludes the root from all negibacteria except Chlorobacteria. Complementarily but less decisively the gracilicute indels exclude the root from the four-phylum gracilicute clade and the flagellar origin argument excludes it from Posibacteria, Eurybacteria, Exoflagellata and Spirochaetes. Thus a multiplicity of evidence excludes the root from all parts of the tree except the putatively earliest diverging eobacterial phyla: the Hadobacteria, Cyanobacteria, and Chlorobacteria. The strong Omp85 argument excludes it from everything except Chlorobacteria and nine other important characters missing from Chlorobacteria corroborate their likely primitive status (Fig. 7). Several important characters indicate that Hadobacteria are more primitive than any other phyla except Chlorobacteria. Anyone wishing to retain the common assumption of a root between neomura and eubacteria as suggested by the first protein paralogue trees would have to disprove all four of the major polarisations deduced from proteasome, cell wall, and flagellar evolution, and suggest a superior interpretation for the evolution of all these features. They would also have to show that the fossil evidence that eubacteria are at least a billion years older than eukaryotes and/or all the evidence that archaebacteria are sisters not ancestors of eukaryotes [1,129] have been fundamentally misinterpreted.

**Figure 10:**
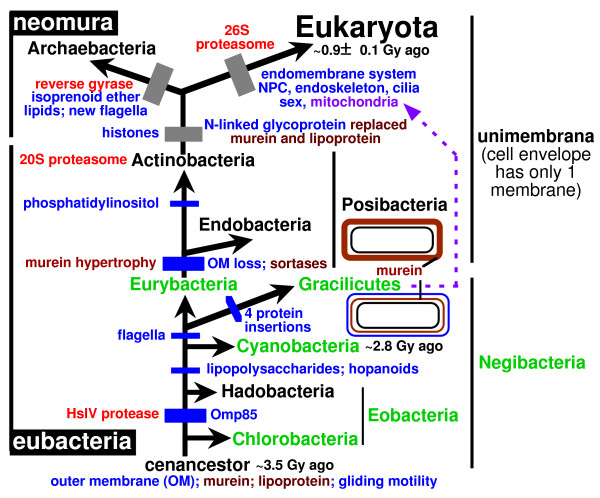
Simplified summary of the 10 major cellular transitions in the history of life. Those discussed and polarized previously [1, 27, 33] are shown by grey bars, while those discussed in detail here for the first time have blue bars; the five shown by thick bars plus the origin of 20S proteasomes (within actinobacteria, so no bar) are especially decisively polarized; evolution in the reverse direction would have been highly improbable, whereas for the four shown by narrow blue bars evolution in the reverse direction would have been mechanistically possible but unparsimonious and have required numerous losses. Evolutionary loss has, however, sometimes played a crucial role, as in the origins of posibacteria and of neomura by the loss of the OM and murein wall respectively. The bacterial groups shown in green are either all photosynthetic (Cyanobacteria) or have a mixture of phototrophs and heterotrophs (the others); the entirely non-photosynthetic bacterial groups are in black. The fundamental changes involving murein peptidoglycan are shown in brown. NPC = nuclear pore complexes, several proteins of which are structurally related to those of coated vesicles, all probably arising in a single burst of gene duplication during the origin of eukaryotes [199]. The dotted line from Gracilicutes to mitochondrion signifies the intracellular enslavement of a probably photosynthetic purple bacterium by a protoeukaryote to make the chimaeric eukaryote cell [33, 129], which must have taken place long after the origin of proteobacteria, even longer after the origin of Gracilicutes and eubacteria, but probably only shortly after the neomuran revolution and bifurcation into archaebacteria (the youngest of all bacterial phyla) and the preeukaryote.

A root within eubacteria has been least popular among molecular evolutionists, yet is the only one strongly supported by palaeontology, as I repeatedly argued [27,27,30,129,141]. I also stressed that the biosphere depends fundamentally on photosynthetic carbon fixation, making it unlikely that large-scale evolution would have progressed until it had evolved, so it probably did so very early [30,31]. I pointed out that five of seven eubacterial phyla previously recognised (five of nine on Fig. 7) contain members using bacteriochlorophyll or chlorophyll for photosynthesis, but each in a distinctively different way with contrasting antenna pigments and reaction centres [30,31]; all those playing a significant role in carbon fixation are negibacteria. I proposed that this diversity arose during the very first major radiation of cells by adaptation to spectrally changing light in successive layers of the first microbial mats and that heterotrophs evolved secondarily to exploit organics leaking from living or dying phototrophs [30,31]. On that interpretation eubacteria are the only primary domain of life, neomura having arisen very much later [30,31]. Proteasome, flagella, murein wall, and outer membrane evolution now strongly corroborate this. They show that posibacteria, actinomycetes, and neomura all arose after the primary radiation of life, probably all substantially later. In my view the widespread assumption that archaebacteria are among the most ancient life forms [19] is profoundly mistaken [1,129].

A root within eubacteria offers a simple perspective on the pattern of protein fold superfamilies across the three domains [119]. As eubacteria are the only primary domain, and thus basal or paraphyletic, it is not in the least surprising that they have no universally present unique superfamilies not found in the other two secondarily derived domains (the surprise expressed in [119] stems from the authors mistaken belief that eubacteria are holophyletic); any superfamilies too important to be lost within the eubacteria would have been retained by their eukaryote and archaebacterial descendants. By contrast, fundamentally important folds that originated during the neomuran revolution would be found in all neomura and no eubacteria. Likewise, those that originated during the somewhat later origin or either eukaryotes or archaebacteria would necessarily be restricted to these derived groups and could not have been passed on to another domain by vertical inheritance. Given the eubacterial root, we can readily see that there were probably only two major bursts of invention of protein folds in the history of life: the origin of many hundreds during the origin of the eubacterial cell and a major addition of novel folds during the origin of eukaryotes [119]. Only one universal archaebacteria-specific fold was invented; the protein that makes the novel archaebacterial base archaeosine, one of very few archaebacteria-specific characters [1]. Thus, in terms of novel protein invention, the origin of archaebacteria was a relatively trivial evolutionary event compared with the origin of eukaryotes, consistent with archaebacteria being the most recent bacterial phylum that evolved billions of years after the major burst of prokaryotic protein innovation occurred. It appears, however, that the ancestor of archaebacteria lost hundreds of fold superfamilies after it diverged from the stem lineage leading to eukaryotes. Thus loss played a much bigger role than innovation in the origin of archaebacteria. In terms of major innovations of protein structure, as well as in cell structure and cell biology, the dichotomy between prokaryotes and eukaryotes is immensely more important than that between eubacteria and archaebacteria. Ribosomal and DNA-handling proteins underwent episodic accelerated evolution during the neomuran revolution, as did rRNA [1], giving the impression on sequence trees that archaebacteria are much more distant from eubacteria than the vast majority of proteins indicates to be the case. These minority proteins and rRNAs thus exaggerate the evolutionary distinctiveness of archaebacteria, which are simply derived, partially degenerate bacteria with novel lipids, flagella and tRNA modifications, and ancestrally greatly reduced genomes and proteomes compared with most eubacteria. It is profoundly misleading to call them a third form of life distinct from prokaryotes and eukaryotes or to consider them as ancient.

Protein fold superfamily trees can clearly be biased by massive convergent protein losses; although some correction for this can be made [119], it is not to be expected that they will reconstruct the actual phylogeny perfectly using present methods. Nonetheless, it is noteworthy that with the notable exception of spirochaetes, for which there is ample other evidence for monophyly, most of the 10 bacterial phyla recognized here appear to be monophyletic or nearly so (including Posibacteria, which is more nearly monophyletic than is Proteobacteria) on the corrected fold superfamily trees [119]. It would be desirable to add data also for Chlorobacteria, the only prokaryote phylum not included, and to carry out a higher resolution cladistic analysis within eubacteria to map superfold innovations and losses in detail across the nine eubacterial phyla to test the tree topology proposed here. Although subject to biases, the fact that *Aquifex *is correctly grouped with the Proteobacteria and *Thermotoga *correctly lies within the unresolved Endobacteria/Eurybacteria branch and that these two hyperthermophiles do not group together, as they often do on sequence trees, is consistent their grouping on many sequence trees probably being a tree reconstruction artefact. It would be particularly interesting to know how many of the 68 non-universal 68 fold superfamilies are absent from Chlorobacteria and at what point on the tree each of them arose. This would enable a complete enumeration of the fold superfamilies present in the cenancestor, likely to be over a thousand.

My present interpretation of eubacterial evolution and rooting of the whole tree is broadly similar to the earlier one [1] but is far stronger in being based on the new eubacterial polarisations shown in Figs 3 and 7, in addition to the detailed neomuran, eukaryote, and archaebacterial origin analyses discussed previously. It also differs in three key respects, each important for the relative timing of early cell evolutionary events. Because of the 12 synapomorphies shared by Hadobacteria and more derived cells (Fig. 3, 8), I now place the root between Chlorobacteria and all other life, or possibly within Chlorobacteria, not between eobacteria and all other life as before [1]. Secondly, I now place cyanobacteria below the gracilicute/eurybacterial divergence, and argue that they are primitively without flagella, not secondarily so [1]. Thirdly, I accept Eurybacteria as definite negibacteria, which places the origin of posibacteria substantially later in evolution than the major glycobacterial radiation of photosynthetic phyla.

### Testing the chlorobacterial rooting

It is a mistake to think that sequence trees are the only way of testing phylogenetic history. Nonetheless, they are important. Several features of the universal tree presented here, especially the relationships among eurybacteria, should be tested by taxonomically much more extensive gene sequencing and phylogenetic analysis of scores of the most conservative genes (with proper controls against lateral gene transfer) using methods best adapted to avoiding those systematic biases that can make it increasingly likely that you will get the wrong tree with high confidence as more genes are added.

However, the best form of testing is to search for other equally strong cladistic characters amenable to decisive transition analysis to see whether they are congruent with or contradict the present rooting. Finding additional characters of this sort is also likely to be the most secure way of deciding whether the root lies within chlorobacteria or beside them. I cannot predict what such unrecognised characters might be, for if I already knew I should have used them in this paper to finally pinpoint the root. But we should expect there to be a number of important characters that in principle might corroborate or refute some of the polarisations that I have made. Obviously, in my previous paper, primarily devoted to the neomuran revolution [1], I missed all those shown in red on Fig. 3 and that were discussed above for the first time. This should stimulate researchers to seek other characters that can be of value, and not falsely to claim that the present conclusions are untestable, should they disagree with them.

The most important kind of characters involves at least several interacting proteins that have undergone very few major transitions in the entire tree. With such characters it is sometimes possible unambiguously to polarise transitions, as I have shown here, so I emphasized them most in the first part of this paper. Indels, gene fusions and presence of absence of particular genes can also be valuable in helping to group taxa together but they risk confusion by multiple losses and lateral transfer. Although the latter can often be detected by sequence phylogeny, it can be notoriously difficult to rule out simple tree reconstruction artefacts, especially in sparsely sampled trees, and some probably overpower even the best methods currently available. Individual gene characters can also seldom be used on their own to polarise transitions. One that did, in conjunction with paralogue trees, is the insertion in the catalytic subunit of neomuran vacuolar ATPase, which demonstrate that the ancestral state for that molecule is the eubacterial version, whilst the neomuran one must be derived [1,116]. A similar argument can be used to support the derived nature of the gracilicute RNA polymerase insertions discussed above.

A second good way of testing the conclusions is by more intensive fundamental and comparative study of the very molecules of the individual macromolecular complexes on which the transition analyses depend. Especially important is analysis of protein diversity in the OM of chlorobacteria and the methods they use for targeting and assembling protein complexes, and the phylogenetic history of the molecules involved. Such studies will provide the hard information necessary to assess critically the validity of the Omp85-based polarisation, and are thus of crucial significance. They could either strengthen or weaken my arguments, or lead to worthwhile improvements in them.

Given the evolutionary importance of Eurybacteria as probable ancestors of Posibacteria, and of Chlorobacteria as being probably the most primitive of all cells, both phyla ought to receive the extensive and intensive study by molecular, cell, and evolutionary biologists that archaebacteria did in response to the stimulating, but partially incorrect, three domains of life theory. Sequencing complete genomes of numerous phyletically diverse Chlorobacteria would provide essential information for testing and refining the present analysis and pinpointing the root of the universal tree precisely, so that we can then work more confidently backwards to deduce how life itself began.

### Ecological considerations

If Endobacteria rather than Chlorobacteria were the most ancient group [79], they could not have sustained an ecosystem by themselves, as all are heterotrophs. No Posibacteria could do that, as none can photosynthesize or carry out net carbon fixation. No unibacteria can carry out photosynthetic carbon fixation. Putting the root between neomura and eubacteria as suggested by a biased subset of paired paralogue trees [15,16] implies the ecological impossibility of a heterotrophic ancestor generating an ecosystem in which posibacterial heterotrophs could diversify sufficiently eventually to evolve into the first negibacterium (despite the mechanistic near impossibility of that discussed above), and only then evolve chlorophyll and photosynthesis. That ecological absurdity is implicit in the widespread, but deeply mistaken, acceptance of that position for the root. If early ecosystems were based on photosynthetic fixation of carbon dioxide they were almost certainly primarily, and probably exclusively, negibacterial. Thus, as previously stressed [142], ecology provides an important argument for polarizing the tree from negibacteria to unibacteria, not the reverse.

Chemolithotrophy in its various forms (including methanogenesis) is likely to be multiply derived rather than the ancestral class of nutrition, irrespective of whether the root is placed beside or within chlorobacteria or between neomura and eubacteria. Being limited by restricted availability of reduced inorganic electron donors none could support an ecosystem on the scale possible with phototrophy. On my present interpretation most forms of chemolithotrophy evolved only after the origin of Proteobacteria, with archaebacterial methanogens being immensely later [129].

Only Chlorobacteria, Eurybacteria, Sphingobacteria, and Proteobacteria have a mix of photoautotrophs and heterotrophs, making them ecologically plausible candidates for the first phylum (though modern Eurybacteria use sunlight only for energy, they fix nitrogen but CO_2_). When I first agued that cells must originally have been photosynthetic negibacteria [58], I initially favoured non-flagellate anaerobic green bacteria as the ancestral cells on account of their relatively simple photosynthesis [1], and later, after learning that of these Chlorobacteria alone also lacked lipopolysaccharide, I argued that they were the best candidates [1,142]. Now the root is excluded from all of them except Chlorobacteria by the Omp85 and flagellar arguments, which is also strongly favoured by the much lower OM complexity generally than in any glycobacteria. Surface waters, where proteobacterial exoflagella and phototaxis are especially useful, were probably largely closed to life by UV radiation before cyanobacteria created the protective ozone layer, as was shallow soil, where Posibacteria dominate by having thick walls and drought-resistant spores. Consequences of these innovations for biogeochemical cycles, isotopic fractionation, and climate are discussed elsewhere [129].

Very early life was probably deep benthic with non-flagellate Chlorobacteria and Cyanobacteria dominant. It is thus ecologically as well as mechanistically simpler for the first cells to have been non-motile; soon they evolved gliding to escape burial by sediment and position themselves optimally within the mat. Flagella might have evolved only after atmospheric oxygenation created an ozone layer, permitting individual cells – especially purple bacteria – to swim and photosynthesize in the upper pelagic zone, and then spirochaete endoflagella evolved to allow these secondary heterotrophs to corkscrew through soft mats. Chlorobacterial gliding needs investigation for possible homology to cyanobacterial slime secretion by junctional pore complexes, conceivably an ancestral gliding mechanism, or to type IV pilus-based gliding – or both.

If the root of the universal tree does indeed lie either beside or within Chlorobacteria, it is likely but not certain that the cenancestor was photosynthetic. If, as seems most likely, the root is between Chlorobacteria and all other organisms rather than within them, the cenancestor was definitely photosynthetic (discounting later lateral transfer of photosynthetic genes, not rigorously demonstrated [143], and an unnecessary complication as the next section explains for the pigment biosynthesis genes: Fig. 9 interpreted their probable vertical evolution cladistically). It is very important to culture strains and sequence genomes from the most deeply diverging chlorobacterial lineages, as this might establish their ancestral phenotype. This plus cladistic/transition analysis should help decide whether Chlorobacteria are holophyletic sisters of all other organisms (Figs 3, 7) or their ancestors, and enable a still more rigorous reconstruction of the last common ancestor of all life. It has been suggested that *Dehalococcoides *had an autotrophic ancestor [121]. Ancestral Chlorobacteria and the last common ancestor of all life were most likely photoheterotrophs [31].

Although I have sometimes thought that the non-CO_2 _fixing photoheterotrophy of Heliobacteria and their apparently simple photosynthetic machinery of Heliobacteria might have been primitive, the robust polarization of numerous other characters (Fig. 3, 7) is incongruent with such a view, so we must now conclude that their apparently simple photosystem is not primitive but the result of radical evolutionary simplification (Fig. 9), as must also be the loss of photosynthetic carbon fixation – presumably a secondary adaptation to a niche with abundant organic material provided ultimately by other photosynthetic phyla, and thus a need to rely on light only for energy. This clarification of the specialised derived status of heliobacteria emphasises the importance of analysing numerous independent cell characters in order to root the tree and not to attempt to distinguish between apparently simple characters that are genuinely primitive and those that are derived merely by studying one system in isolation, whether that be ribosomes, photosynthesis, flagella, wall and OM biosynthesis, OM protein-targeting, secretion systems, or proteolysis. All were needed to reach the present decisive conclusion. It is decisive because only one root position is congruent with them all.

### Evolution of nitrogen fixation

As an example of the profoundly different and much simpler conclusions that the present rooting of the tree can lead to, I shall conclude by considering the evolution of nitrogen fixation, which had major ecological and biogeochemical importance. It is accepted that both multiprotein reductase components of nitrogenase are evolutionarily related to the two major components of the enzymes that carry out key reductions of the photosynthetic pigment precursors protochlorophyllide and chlorophyllide [114]. Recently two alternative elaborate interpretations involving numerous hypothetical lateral gene transfers were put forward based on the erroneous assumption that the root lies between neomura and eubacteria [114]. Both assumed that the Nif genes responsible for nitrogen fixation evolved before protochlorophyllide and chlorophyllide reductases and that pigment biosynthesis evolved from them. It was not explained how nitrogen fixation and extensive cellular evolution was possible in the absence of photosynthesis, but implied that ecosystems may originally have been based on methanogenesis. One scenario assumed that nitrogen fixation evolved in the last common ancestor of all life, and the other assumed that it arose in methanogens and was transferred to eubacteria prior to the origin of chlorophylls.

A phylogenetically much simpler and evolutionarily and ecologically far more plausible interpretation is that group II Nif genes evolved from the pigment biosynthesis genes in the bacterial cenancestor but that group I Nif genes arose only during the glycobacterial revolution after the origin of oxygenic photosynthesis, and that inheritance has been almost exclusively vertical (Fig. 7). Nif genes are absent from Hadobacteria. Although they were not previously found in Chlorobacteria [104], I found two NifD homologues by BLAST in the thermophilic non-photosynthetic chlorobacterium *Dehalococcoides *(one hits Chlorobiales much more strongly than any other groups, E value e^-86 ^and another does so with e^-58^) and one in the mesophilic photosynthetic chlorobacterium *Oscillochloris*, which has recently been shown also to have NifH [144]. As these chlorobacterial Nif genes seem all to be of the anaerobically adapted group II type found also in green sulphur bacteria, I suggest that this putatively ancestral type evolved from either protochlorophyllide or chlorophyllide reductase by duplicating a pigment synthesis operon, marked divergence to allow attachment of an FeMo cofactor, and subsequent additional duplications. Later during the glycobacterial revolution the rise in oxygen levels made an ability to reduce nitrogen in its presence important, which required a new type of nitrogenase, type I, characteristic of cyanobacteria and proteobacteria, which I suggest arose in a rapid burst of evolution (quantum evolution: [1,129]) to become the ancestor of group I nitrogenases. I suggest that the group I Nif operon, which contains several paralogous genes, arose in the aerobic common ancestor of cyanobacteria and gracilicutes with at least some means of separating it from oxygen. When the ancestor of green sulphur bacteria (Chlorobiales) became anaerobic after core gracilicutes diverged to yield proteobacteria and Sphingobacteria, it retained the group II enzyme but lost the group I enzyme, whereas the ancestrally facultatively aerobic Proteobacteria lost the group II enzyme and retained the new aerobic group I enzyme instead. Much later still a group II enzyme was laterally transferred from a green sulphur bacterium (precisely as suggested previously: [104]) to the common ancestor of the archaebacterial Methanosarcinales (a group known to have acquired many eubacterial genes [41]). My interpretation requires no other lateral transfer events, but assumes that nitrogen fixation was lost by the neomuran common ancestor and frequently within phyla that have it. Raymond et al. [114] pointed out evidence for such loss in *Fusobacterium*, which has four Nif homologues but does not fix nitrogen, and also remarked that 'the individual genes composing the core HDKEN operon have remarkably similar phylogenetic histories despite instances of gene duplication, rearrangement, and loss apparent in the records of multiple genomes.' This indicates that lateral gene transfer has been much rarer for *nif *genes than traditionally assumed. With the correct rooting in eobacteria even fewer need be postulated than they supposed.

I further suggest that type III Mo-independent nitrogenases, present only in Proteobacteria and the archaebacterial Methanosarcinales evolved only later – well after flagella in Proteobacteria, when their more mobile cells were more likely to colonise Mo-poor habitats. By contrast Raymond et al. suggested that although some Mo-independent nitrogenase evolved recently thus, the main group III clade might be more ancient, but recognised that if that were so 'what remains enigmatic is why all alternative nitrogenases studied so far are found only in organisms that also have Mo-dependent enzymes'. Accepting the chlorobacterial root, and the highly derived nature of type III nitrogenase also simply solves the enigma and also does not require that nitrogenase implausibly evolved at the beginning of life as in their first scenario, and reduces the number of lateral gene transfer events; in contrast to [114] none need be implausibly postulated into groups that already have Nif genes.

I also differ in my interpretation of the so-called 'group IV' Nif-related proteins, which appeared as non-holophyletic long branches on their trees [114], and which typically are not part of a Nif operon, and are functionally uncharacterised; there is no evidence that they are involved in nitrogen fixation. I suggest that the simplest interpretation of these is that they are all secondarily derived from Nif I-III proteins by loss of nitrogenase function and with the acquisition of a novel (still unknown) function that allows them to evolve much faster than their Nif ancestors or those involved in pigment synthesis. Their very long branches should not be interpreted as evidence for great antiquity, as suggested previously, but as rapid evolution following a loss of their original function. Raymond et al. [114] suggested that very explanation for the *Fusobacterium *protein. I suggest that it is probably true of all of them. The shift of function need not have occurred once only; it could have occurred independently in Proteobacteria, Eurybacteria/Endobacteria and Methanosarcinales, their deep positions on the tree simply being an artefact of exclusion of these sequences from their far shorter-branch ancestral clades. Thus I suspect that they are not a natural group, but polyphyletic derivatives of the three main Nif clades. This is very strongly supported by the fact that the proteobacterial representatives in 'group IV' have many-fold longer branches than do proteobacterial group I enzymes; they must have evolved many times faster within the very same cell lineages.

Similar long-branch artefacts, I suggest, account for the relative positions of groups I-III on the tree. Quantum evolution postulated above at the base of the group II clade followed by a systematically higher rate of change in the enzymes of these anaerobes would also lead to long-branch exclusion from the group I branch, which I argue is probably not a clade but really paraphyletic. I suggest that substitution of iron or vanadium for molybdenum to form the group III enzymes (probably independently [114]) can also accelerate evolution and cause similar long-branch exclusion. I feel unsure whether the grouping of the archaebacterial sequences with the proteobacterial group III enzymes is genuine or another long-branch artefact. If that relationship is genuine, it implies a lateral gene transfer from proteobacteria to Methanosarcinales, independently of that from Chlorobiales. Although not impossible, given the evidence that this archaebacterial group acquired hundreds of genes from eubacteria [41], it is not obviously correct. They may instead be much faster evolving paralogues that evolved within Methanosarcinales from the group II genes originally acquired from Chlorobiales and therefore be wrongly placed on the tree.

The Nif tree further illustrates the severe hazards of interpreting paralogue trees when major shifts in function dramatically change evolutionary rates. Consider now the pigment synthesis paralogues. It would be absurd to suppose that their evolution has been even remotely clock-like since the protochlorophyllide or chlorophyllide reductase diverged from a common ancestor. The depth of the whole clade is about six times that of its cyanobacterial part. Given that cyanobacteria must have evolved at least 2.2 Gy ago, as shown both by the morphological fossil record and the timing of the great oxygenation event [129,134], assuming a clock would place the time of divergence of those genes as ~13.2 Gy, nearly three times the age of the earth, and somewhat older than the universe. Clearly the rate of evolution of these two paralogues must have been immensely greater soon after they diverged and slowed down dramatically by the time cyanobacteria evolved. The fundamental problem that makes such protein paralogues worse than useless for rooting trees [1] is superbly illustrated by this pigment reductase tree. Each will be such a long branch outgroup that it will give an entirely artifactual mid-point rooting to each tree. We cannot therefore conclude that the root is where they show it to be.

I hope that I have convinced the reader that the root should be on the branch from *Chloroflexus *to the rest of eubacteria on both the protochlorophyllide and chlorophyllide reductase subtrees. Yet both put it between the two green bacterial phyla with chlorosomes and proteobacteria. In the past this has been misinterpreted as evidence for lateral gene transfer between the green bacteria. It is not. It is just a bad tree, dimensionally hugely distorted by quantum evolution and misrooted by paralogue rooting that is necessarily misled by gross long-branch artefacts. If, as argued here and previously [1], the bacteriochlorophylls used by chlorosomes are the ancestral state, the closer grouping of the *Chlorobium *and *Chloroflexus *enzymes merely reflects a slower rate of evolution and the lack of any special acceleration because the pigment type was essentially unchanged. I argue that when cyanobacteria evolved chlorophylls instead of bacteriochlorophyll, and lost chlorosomes, there was another transient large change to the reductase enzyme, a temporary massive rate acceleration that artifactually stretched the stems of the tree that separate cyanobacteria from both groups of green bacteria. Likewise, when later proteobacteria lost chlorosomes and evolved a new set of bacteriochlorophylls, quantum evolution of this enzyme occurred yet again. Thus the degree of separation of the major branches on the two reductase subtrees mainly reflects the change in function for making different pigments billions of years ago rather than the relatively slight divergences that have occurred since. This paralogue tree is profoundly non-clock-like, like all those that appear to support a rooting between neomura and thus profoundly misleading about the relative timing of evolutionary events unless it is very critically interpreted in the light of independent evidence, which must now become the norm for all paralogue trees. The long internal stems that separate them from the Nif proteins likewise reflect rapid quantum evolution during the origin of nitrogen fixation, and do not accurately indicate elapsed time; by the same argument as above, applying a simple clock to Nif half of the tree would give an absurd separation between Nifs and pigment reductases before the universe began. Molecules can only be semi-clock like in their evolution when there is little or no significant change of function – and often not even then, especially if disparate lineages are compared.

### Broader implications of the chlorobacterial root of the tree

All polarizations of evolutionary change discussed above are congruent with the root being between Chlorobacteria and all other organisms (Figs 3, 7) or within Chlorobacteria. Here and previously [1,28] I have given reasons why any other position is contradicted by 1–10 of the more decisive polarizations shown in Fig. 7; the popular idea of a root between neomura and eubacteria is strongly contradicted by three of them. I conclude that the last ancestor of all life was a non-flagellate, non-spore-forming, eubacterium with acyl-ester membrane lipids, a murein peptidoglycan wall, and fully developed eubacterial molecular biology and cell division mechanisms. It was a negibacterium with two membranes, the OM being attached to murein by lipoproteins, not a unibacterium with one, nor a primitive incompetent progenote; however, its cell envelope was probably distinctly simpler in many respects than those of glycobacteria, the best known, but more advanced negibacteria. We cannot confidently say if it was a heterotroph or a phototroph, but photoheterotrophy with separately differentiated respiratory and photosynthetic chains as in *Chloroflexus *is most likely – unless the root is within Chlorobacteria on a purely non-photosynthetic branch.

Previous literature statements about 'early branching eubacteria' are virtually all suspect, especially for *Aquifex*, clearly a thiobacterial proteobacterium seriously misplaced on rRNA and some other trees, and probably also wrong for the eurybacterium *Thermotoga*. Probably only Chlorobacteria are early diverging, though Hadobacteria might be nearly as old. The conventional view that the first cell had one membrane only is merely an assumption that has never been justified by careful phylogenetic arguments; the assumption that the universal root lies within Posibacteria [73] derives solely from an argument based on the supposedly shared absence in Posibacteria of a segment of Hsp70 and its ancient paralogue MREB; however, more accurate alignment of this region shows that these missing regions only partially overlap, making it likely that a secondary deletion occurred in Hsp70 of Posibacteria [145]; if so, this deletion is a synapomorphy for Posibacteria and further evidence for their being derived, not the ancestral state; moreover, as Hsp70 and MREB are sisters, the assumption that the segment missing in MREB was absent in their common ancestor is arbitrary and no help in rooting the tree. The most widespread assumption that the root is between archaebacteria and eubacteria [19] is based purely on a handful of information-poor single-gene paralogue trees [15,16], all technically flawed [1,2,116], and is contradicted by a combination of the fossil record and molecular cladistic evidence for the holophyly of archaebacteria and a direct relationship between neomura and actinobacteria [1], and by several of the 13 novel transition analyses discussed here.

By contrast, the chlorobacterial root of the tree is consistent with the fossil record and strongly supported by transition analysis of many key characters and by congruence testing – including, of course, compatibility with sequence trees, provided that their potentially misleading quirks are critically taken into account, as in the examples discussed here. It gives a radically new and more confident perspective to molecular and cell evolution. Interpretations based on the so-called 'standard model' of a primary split for life between archaebacteria and eubacteria [19] were based on an incredibly narrow line of evidence, now shown to be fundamentally flawed, and need radical revision. Assumed lateral gene transfers and the fossil record both need critical reinterpretation in light of the new and more secure rooting, as illustrated above for the Nif proteins, and previously for isoprenoid synthesis enzymes [1].

Life probably began between about 3 and 3.5 Gy ago, with the earlier date more likely if the stromatolites, filamentous putative fossils and light carbon isotope levels in that period are mostly biogenic. Biomarkers dating from 2.8 Gy and carbon and sulphur isotopic changes between 3 and 2.4 billion years ago and the evidence for consistently increased oxygen levels since 2.45 Gy ago [134,146-150], are consistent with the view that cyanobacteria and all the other negibacterial phyla and proteobacterial biological sulphate reduction had already come into existence by 2.4 Gy ago. I suggest that the sudden negibacterial radiation seen on all molecular trees represents a genuinely rapid radiation of photosynthetic bacteria and secondary heterotrophs around 2.8–3.0 Gy ago: i.e. the origin of all negibacterial phyla except chlorobacteria, which are likely to be somewhat older – a glycobacterial big bang triggered by the origin of oxygenic photosynthesis associated with photosystem duplication in the common ancestor of glycobacteria and hadobacteria, as argued above. All transitions between negibacterial phyla involved phototrophs as the ancestral state. It is unlikely that oxygenic photosynthesis originated before 2.9 Gy ago given the evidence from mass independent sulphur isotope fraction in the much of the Archaean [149,151]. Therefore the filamentous putative fossils of the period 3–3.5 Gy ago [152-155] would be more likely to be chlorobacteria than cyanobacteria [37] (if they are indeed biogenic, which is non-proven but reasonably plausible for some [37,155], despite serious criticisms of the earliest examples [156,157]).

As Endobacteria are nested within Eurybacteria, the origin of the purely heterotrophic Posibacteria probably significantly post-dated the major negibacterial big bang radiation; they are unlikely to be much older than two billion years; if Endobacteria are paraphyletic actinobacteria could be still younger. I suspect that some of the largest most complex microfossils that first appear nearly 1.5 Gy ago may be actinomycetes rather than stem eukaryotes, for, in my view, none of their morphological features require either a cytoskeleton or an endomembrane system for their formation, contrary to assertions that they do [158] – there is no reason to place them within any known eukaryote group. If the major 'big bang radiation' of eukaryotes [27] was later than sometimes supposed (perhaps only 800–1100 My ago [1,34,129]), and archaebacteria are really their sisters [1], archaebacteria cannot be much older, making it invalid to invoke their biogenic methane as greenhouse gas to solve the faint early sun problem and prevent Archaean global freezing [147,148]. Alternative explanations [159] are possible for the light carbon isotopes around 2.8 Gy often attributed to methanogenesis plus methylotrophy [160,161]. In a parallel more detailed discussion of the fossil record in the light of the new rooting of the tree argued here I explain how this light-isotopic signal could have been produced simply by eubacteria in stratified microbial mats without any participation from methanogens [129]; these isotopic data are not specific evidence for methanogens and thus do not contradict the late origin of archaebacteria, contrary to widespread assumptions.

Perhaps the origin of oxygenic photosynthesis about 2.9 Gy ago, just before the origin of cyanobacteria (>2.8 Gy based on hopanoid biomarkers [162]), oxidized such abiotically generated methane as may have been present in the early Archaean atmosphere (no basis yet exists for assessing its likely level) and caused the world's first glaciation (2.9 Gy ago [163]) and the burgeoning of cyanobacteria 2.5–2.7 Gy ago depleted an ancient protective CO_2 _blanket [164] by carbon fixation enough to trigger the 10 My global freeze-up ~2.4 Gy ago [165]. Recovery from such global glaciation was also probably slow because it required accumulation of very high CO_2 _levels [166]. Earth probably approached its modern quasi-steady state only after the burgeoning of cyanobacteria when the burial of fixed carbon and oxidation of sulphur and iron minerals by biogenic oxygen reached a quasi steady state during the great oxygenation event of the atmosphere and surface rocks 2.0–2.45 Gy ago [134,147]. If archaebacterial methanogenesis originated soon after 800 My ago [1], the first major pulse of biogenic methane could have been climatically very destabilizing before methanotrophy originated; a sudden major new source of this greenhouse gas, 21 times more powerful in trapping heat than CO_2_, might have triggered the Neoproterozoic snowball earth episodes ~710 and ~635 My ago [167] by causing sudden global warming, thus inducing climatic oscillation into near global ice-ages, that would eventually have settled down after methanogenesis and methanotrophy became quantitatively balanced; see [129] for details and references. On this view both major global snowball earth episodes in the history of our planet could have been caused indirectly by the origin of new types of bacterial physiology. Life before and after this second huge climatic destabilization remained rather simple and little changed [168], with eukaryotes unambiguously identifiable to modern phyla emerging only in the very late Neoproterozoic shortly before the Cambrian explosion of both animals and protists [1,169]. It is likely that this late eukaryotic explosion of life was primarily prevented earlier by the difficulty and thus lateness of the complex transition from bacteria to the eukaryotic cell [27,29,56] and especially of its prerequisite, the probably immediately preceding neomuran revolution [129], but sustained evolution of greater complexity may also have required the greater climatic and ecological stability that set in after about 600 My ago because all major forms of bacterial metabolism had already evolved and their bearers multiplied into a trophic quasi-steady state. More accurately dating the onset of each type of metabolism by better integration of palaeontological and molecular evidence [146], will enable such far-reaching interpretations to be tested more thoroughly.

### Lipid substitution in bacterial membrane evolution

At the request of a referee I introduced this and the following two sections to comment of some of the misconceptions in a recent paper by Koonin and Martin [170], some of which are more widely held and thus merit refutation in detail. That paper slightly extends Martin and Russell's earlier one [108] that combined Russell's interesting ideas on precellular chemistry before the origin of life with the fallacious widespread assumption that the root of the tree is between neomura and eubacteria, which the present paper refutes in detail, plus the also ill-founded suggestion that lipid membranes evolved independently in neomura and eubacteria [109]. Koonin and Martin [170] criticize the view that eubacteria are ancestral to archaebacteria [1,5,29,79,81,129] because it entails the substitution of acyl ester lipids by isoprenoid ether lipids in the ancestor of archaebacteria. They object to this because they claim 'no known prokaryotes have undergone any vaguely similar cataclysmic lipid transition'. But 'cataclysmic' is tendentious and question begging. Their own scenario necessarily entails precisely the opposite transition: from isoprenoid ethers in the supposed archaebacterial ancestor of eukaryotes by acyl esters supplied by the proteobacterium enslaved as a mitochondrion. They do not attempt to explain why the transition would be less cataclysmic in that direction, or provide any reason why a cell could not survive a replacement of acyl esters by isoprenoid ethers.

More fundamentally, setting aside the emotive 'cataclysmic', is it actually true that no prokaryote has ever 'undergone any vaguely similar lipid transition'? Of course not. As discussed above, several evolutionary replacements of the lipids of outer leaflet of the negibacterial OM are well documented. First was the replacement of ordinary phospholipids by LPS in the ancestral glycobacteria; later this was replaced in *Sphingomonas *and six related genera of α-proteobacteria (and independently by most spirochaetes) by glycosphingolipid (D-glucuronosylceramide), and still later by phosphatidyl choline independently in chloroplasts and mitochondria. Clearly they are at least 'vaguely similar' cases of lipid substitution. Even had their assertion been true, it would have been an evolutionarily naïve and illogical objection. One cannot reasonably argue against the occurrence of a unique evolutionary transition, such as the origin of feathers or the replacement of acyl esters by isoprenoid ethers on the grounds that it never occurred elsewhere! There is no fundamental mechanistic difficulty in lipid replacement if the transitional stages had both lipid types and gradual adaptation occurred. There are many cases where eubacteria have supplemented their phospholipids by other kinds and have mixed membrane composition, e.g. by sphingolipids in many sphingobacteria and ether-linked glycerolipids in many eubacterial hyperthermophiles, e.g. *Aquifex*, *Ammonifex *and *Thermodesulfobacterium *[171]. The repeated independent evolution of ether lipids in hyperthermophiles strongly supports the thesis that such bonds are a specific adaptation to hyperthermophily and hot acid [1,30] in the ancestral archaebacterium. I argued that those lipids were ancestrally tetraethers spanning the whole bilayer, and stressed that as posibacteria can already make isoprenoids [1] and have the enzyme glycerol-1-phosphate dehydrogenase that makes the sn-1-glycerol phosphate backbone to which they are added in archaebacteria [18], that the origin of such lipids by an actinobacterial derivative was mechanistically and phylogenetically highly plausible. The only additional step was the loss of the acyl esters, also perfectly plausible. Thus the precursors were all present in the actinobacterial ancestor and the selective advantage of the replacement is very clear in that direction not the reverse. I have never seen a refutation of these arguments from Martin or anyone.

The gulf between archaebacterial and eubacterial lipids is also made less by the discovery that membrane spanning tetraether dialkyl glycerol lipids are abundant in peat [171]; if they are made by eubacteria, as suggested by their sn-3-glycerophosphate stereochemistry [171], this would mean that some eubacteria evolved tetraether lipids independently of archaebacteria. If instead they are made by archaebacteria, this would show that some archaebacteria can make syn-1,2 alkyl glycerolipids like eubacteria. Change in membrane lipid composition is not as difficult as Koonin and Martin claim.

They also fail to address the similarities in eubacterial murein peptidoglycan biosynthesis and neomuran N-linked glycoprotein biosynthesis, with respect to the key involvement of N-acetylglucosamine and the transport of hydrophilic precursors across the cytoplasmic membrane by a long chain isoprenol (dolichol or undecaprenol respectively) to which I drew attention in 1957 when proposing the neomuran theory [29]. The latter in particular as emphasized above implies that the eubacterium/neomuran transition took place in a cell with complex cytoplasmic membrane and ability to make both long chain isoprenols and N-acetyl glucosamine. Treating that transition as being precellular [108,109,170,172,173] is cell biologically absurd. It is also unsound from the point of view of protein evolution; 683 protein fold superfamilies are shared by eubacteria and archaebacteria [119]; probably most of these were present in the transitional organism, making it proteomically far too complex a cell not to have had a DNA genome, contrary to their unsound assumption [170].

They attempted to meet the criticism that the cenancestor must have had a lipid membrane to explain the universal presence of homologous SRP mechanisms and proton pumping ATPases in cells [18] by suggesting for the first time that their hypothetical inorganic walled intermediate between neomura and eubacteria also had lipid membranes of fatty acids made prebiotically, not biosynthetically [170]. While it is nice that they are beginning to concede the importance of lipids, this has every appearance of a desperate attempt to save a totally untenable hypothesis from fatal criticism, rather than a well-thought out theory. They do not hazard a guess as to the function or selective advantage of such early membranes. Nor do they cite or refute my arguments that membranes must have played a key role in the origin of organismal complexity, in association with genes and catalysts, by providing a supragenic unit capable of inheritance and variation on which selection could act [31]. They seem not to understand in the slightest the required nature of such an organismal entity. It must be able to vary in its rate of growth and multiplication and the associated genes must influence these properties heritably.

### Cells, not genes, are the key units of selection and heredity

Although Koonin and Martin [170] call their compartments discrete units, they are not shown thus on their diagrams, but are joined in a network – the opposite of discreteness. They suggest that new ones are formed by 'precipitation at the ocean interface'. Thus it seems that old compartments do not grow or divide or generate new ones directly. Thus they are NOT units of multiplication on which selection might act and are not units of heredity, unlike cells or the membranous obcells that I argue preceded and created negibacterial cells [31]. What phenotypes do compartments have and how could they be improved? Calling them 'discrete units' is meaningless verbalism irrelevant to the need to have discrete supramolecular units that can grow and divide and transmit collective phenotypic characters to their offspring, and whose numbers can be changed by competition between alternative phenotypically different variants of those supramolecular units [31]. The claim that compartments are 'units on which selection can act' [170] seems deeply mistaken. These compartments do not remotely fulfil the requirements of a proto-organism on which selection can act and lead to increased complexity [31]. As old ones are not directly involved in the formation of new ones, they do not reproduce and are entirely unbiological and irrelevant to the origin of cells. Nothing is said about their organismal phenotypic properties, nor how genes could change them so as to increase their rate of reproduction (how can they when they don't even reproduce?). Compartments are also supposedly porous, with genes and products diffusing among them [170]; thus they are not discrete in the genetic sense that matters for evolution. The compartments are merely a heterogeneous environment within which genes might theoretically evolve; it is not obvious from their vague statements that these compartments would be any better able than molecular evolution in a homogeneous medium (that they rightly criticize as inadequate) to provide real units capable of selection for cooperative proto-organismal behaviour.

The basic problem of these Martin/Russell/Koonin papers is that they conflate three independent problems: precellular evolution, the nature of the last common ancestor (clearly cellular, clearly negibacterial), and the nature of the eubacterium/archaebacterium transition, none of which has anything to do with any of the others, according to my phylogenetic analysis [1,31]. Russell's ideas are irrelevant to the latter two, but are of potential real interest to the first. These papers are phylogenetically profoundly wrong and conceptually defective in failing to be cell biologically or evolutionarily realistic.

The suggestion that the first membranes could come from prebiotic fatty acids [170] is neither unreasonable nor novel, but probably irrelevant to the early function of SRP and proton pumping. As I previously pointed out [31], prebiotic fatty acids were probably so short that membranes made solely of them could have been too permeable to support chemiosmotic mechanisms. I also pointed out that the first membranes must have been made from prebiotic lipids made before lipid biosynthesis evolved; at least that is something we agree on, but it is entirely phylogenetically wrong to link this sensible requirement to the eubacterial/neomuran transition. However precellular membrane lipids are more likely to have been a chemically heterogeneous mixture of amphipathic molecules [31] and it is a pure guess to suggest they were fatty acids alone. If they were, that would be a reason for the first biosynthesized lipids more likely having been acyl esters than isoprenoids, i.e. eubacterial in nature. Thus their suggestion [170] leads to a (weak) argument for eubacteria first and archaebacteria later! As isoprenoid biosynthesis is so complex and requires many lipid-embedded enzymes (i.e. relatively complex pre-existing membranes) it presents a greater chicken-and-egg problem than does the origin of acyl ester lipids. Fatty acid biosynthesis does not depend on membrane-embedded proteins, but on water soluble ones, so may have been easier to evolve by simply extending pre-existing prebiotic fatty acids.

### Limitations and value of genomics

Koonin and Martin also object to the actinobacterial ancestry of neomura on the spurious grounds that 'no genome wide data implicate either actinobacteria or low GC Gram-positive bacteria as ancestors of archaebacteria'. Even were it true, that would be no reason for preferring their theory, for no genome-wide data, or any other data, implicate mineral compartments as the immediate ancestors of archaebacteria! In fact, to his frequent embarrassment, Martin himself showed by analyzing 24,990 genes of the flowering plant *Arabidopsis *that more of them are related both by BLAST and tree criteria to posibacteria than to any other phylum of eubacteria except cyanobacteria (which we know were enslaved as chloroplasts); roughly 800 had highest BLAST hits to the endobacterium *Bacillus *and ~400 to the actinobacterium *Mycobacterium*, compared with only 100–200 for archaebacteria [174]. Moreover, *Mycobacterium *(Actinobacteria) genes share a branch with *Arabidopsis *on trees much more often (148 genes) than do genes of any non-cyanobacterial eubacteria (2–71 genes depending on species; 2–31 only in Proteobacteria despite their contribution via mitochondria), with Endobacteria ranking second (93 genes) [174]; only 3–18 archaebacterial genes share a branch with *Arabidopsis *despite the established monophyly of neomura. The reason for this poor showing of actinobacteria compared with archaebacteria is probably that many genes present in the neomuran ancestor were dramatically changed by quantum evolution in the ancestral eukaryote alone (so that yeast genes are much more similar to *Arabidopsis *genes than are most archaebacterial genes), and that many ancestral neomuran genes were lost by archaebacteria [1], whereas related homologues remained in Posibacteria; some genes also may have undergone such quantum evolution in the archaebacterial ancestor alone, with similar consequences for higher perceived similarity between posibacterial and *Arabidopsis *genes. The genes with strongest similarity between *Arabidopsis *and posibacteria are likely to be those that did not undergo marked changes during eukaryogenesis but evolved faster in or were lost by yeast and archaebacteria. Whether archaebacteria are ancestors of eubacteria as Martin thinks or their sisters, as I argue, his genomic finding [174] can reasonably be construed as genome-wide evidence supporting actinobacteria as the closest eubacterial relatives of neomura and thus of archaebacteria. This is genome-wide data and unambiguous; 148 genes support my thesis on trees, precisely contrary to Koonin and Martin's assertion [170] – none, of course, support inorganic compartments as ancestors. But this evidence is relatively weak compared with the arguments presented here from proteasomes and phosphatidylinositol for an actinobacterial/archaebacterial connection.

Based on 191 complete genomes from all three domains, a recent study selected the 31 most reliable universal proteins for a multigene tree that placed low-GC Gram-positives (Endobacteria) as sisters to neomura with strong bootstrap support, and also showed archaebacteria as strongly holophyletic [175], i.e. strongly refuting Martin and Müller's 'hydrogen hypothesis' that archaebacteria are ancestral to eukaryotes [176] and supporting my argument that archaebacteria are sisters of eukaryotes [1,29,129], and related to Posibacteria more closely than to any Negibacteria. Refutation of a direct archaebacterial ancestry of eukaryotes (i.e. archaebacterial paraphyly) is important, because only if eukaryotes were derived from rather than sisters of archaebacteria is there any reason for postulating that the ancestors of eukaryotes ever had archaebacterial lipids [176]; if they are sisters it is more parsimonious to suppose that acyl ester lipids were inherited vertically by eukaryotes from their eubacterial ancestors [29]. Thus there is no reason whatever for the unparsimonious hypothesis that eukaryotes replaced archaebacterial lipids by eubacterial lipids from the mitochondrion (anyway inadequate as proteobacteria lack phosphatidyl inositol and could not have provided this fundamental eukaryotic constituent, unlike actinobacteria), contrary to [108].

In his own genome-wide analysis [174], many times more genes support an actinobacterial ancestry for eukaryotes (which Martin wrongly rejects) than they do for an archaebacterial ancestry for eukaryotes (which he wrongly accepts, despite all the other evidence against it and for a sister relationship instead [1,129,175]). The fact that his genomic evidence does not even suggest a eukaryote relationship with archaebacteria, which all biologists accept, proves the severe limitations of such crude 'genome-wide' criteria, unless critically interpreted by explicit evolutionary reasoning such as transition analysis of key characters. Without this genomics is often unilluminating and sometimes positively misleading; it risks becoming a substitute for critical thought. It is essential to recognize that molecular evolution is grossly non-uniform across genomes, lineages, and time. Acceptance, often implicit rather than explicit, of the false assumption of uniformism has caused much misinterpretation [129].

Properly complemented by well-grounded biological theory, experiments and organismal data, the copious information from genomics is most valuable. But Koonin and Martin [170] overvalue genomic data compared with cell biological and organismal considerations. Many bioinformatic gene counting exercises are very naïve in ignoring the likelihood of quantum evolution or massive gene loss during the neomuran revolution, origin of eukaryotes, and origin of archaebacteria; I present a sounder interpretation of one such study bearing on the nature of the neomuran ancestor in some detail elsewhere [106]. In short, gene losses and quantum evolution of sequences beyond recognition grossly distort such statistics. We must consider bacterial evolution as a problem of organismal evolution and not base conclusions only on the sequences of the non-representative minority of genes that evolved sufficiently trivially to still allow sequence alignment and tree making.

### Concluding remarks: put the organism back into bacteriology

Contrary to molecular pessimism that neglects organismal properties of bacteria [177], one can plant the tree of life; in my view the arguments in this and earlier papers [1,5,178] together unambiguously establish that the universal root is within negibacteria, and that it is most probably among the non-flagellate, photosynthetic, gliding negibacteria, and most likely of all among or beside the chlorobacteria. For understanding their evolution, bacteria (=prokaryotes) must be thought of as organisms [1], not disembodied genes [177]. The thoroughly sound concept of a bacterium [179] – and (equivalently) prokaryotes – as a distinctive kind of organism is periodically subjected to muddled but vigorous attacks by a few who seem to think that a limited rRNA perspective is adequate for all biology, e.g. [180].

The most recent such assault unwisely seeks to abolish the very name prokaryote, wrongly asserting that 'no one can define what is a prokaryote, only what it is not' [181]. That is untrue: all prokaryotes have cells where the ribosomes, SRPs, and DNA attach directly to the main surface membrane of the cell, the cytoplasmic membrane, and where the main chromosome is a single replicon with a single origin controlled by DnaA-like proteins. Those are universal positive characters of prokaryotes never found in eukaryotes, as I personally told that author over a decade ago. Why deny the facts and falsely claim that the concept of a prokaryote is unscientific? The prokaryote-eukaryote concept is living and well as the most important cellular dichotomy in all nature [29,131]; it is emphatically not 'an incorrect model for evolution' [181].

Pace [181] accepts eubacteria as a taxon and name because he does not realize the strength of my arguments that they are paraphyletic [1,29], or that the paralogue rooting evidence for their holophyly is so weak, profoundly misleading [1], and contradicted by other paralogue trees [1,116], not because eubacteria have any more organismally important, universally shared positive characters than do prokaryotes. One can easily say that all eubacteria lack SRP helix 19 and N-linked glycoproteins ('what they are not'), but apart from DNA gyrase (also laterally transferred into some archaebacteria and symbiogenetically into eukaryotes) the universal positive characters of eubacteria ('what they are') are primarily just a few rRNA signatures [182] and RNA polymerase and DNA replication enzymatic details [1] that are probably not of great organismal significance. But all are useful as extra examples of universal characters that refute the false claim by many cladists that paraphyletic groups never have any universal positive characters; though in all these cases simplistic cladistic dogmas are complicated by their symbiogenetic infusion into eukaryotes also, especially as chloroplasts. Murein peptidoglycan, lipoprotein and lipoprotein signal peptidase are ancestral eubacterial synapomorphies that have been lost at least once (e.g. all by Mollicutes) within eubacteria in addition to their loss by the ancestral neomuran; these non-universal synapomorphies are organismally more important than the universal rRNA, DNA handling enzyme ones (lost by the neomuran ancestor only), despite being more often lost. But both bacteria (=prokaryotes) and eubacteria are important concepts and biological groups despite their paraphyly and are in principle acceptable as taxa. I do not use eubacteria as a taxon because within prokaryotes the negibacteria/unibacteria dichotomy is more ancient and fundamental than the eubacteria/archaebacteria one and because having subkingdoms Negibacteria and Unibacteria is a more balanced classification of bacterial diversity [69]. The terms group, taxon, and clade are not synonyms, as is explained in detail in [69]. Rejecting any of the names eubacteria, prokaryotes or bacteria just because these groups are paraphyletic would be extremely stupid and very harmful to our ability to communicate sensibly about evolution and cell diversity. The so-called 'three domains' [182] are simply 'big groups' of unequal significance; two are clades (eukaryotes and archaebacteria) best treated as taxa, but not deserving equal rank [see [69], for discussion of ranking], one is a paraphyletic grade (eubacteria) that is too heterogeneous to be useful as a taxon in a formal subdivision of the kingdom Bacteria (Tables 1 and 2).

The claim that there may be no real tree of life because of lateral gene transfer [177] is also profoundly mistaken and stems from muddling gene trees and organismal trees; only lateral transfer of cells (not genes), as in sexual gamete fusion and in symbiogenesis, both restricted to eukaryotes, makes organismal phylogeny non-tree like. The tree of life is an organismal phylogeny. All cellular inheritance in prokaryotes is vertical, not horizontal, as cell fusion never occurs except occasionally in *Streptomyces*. It **is **meaningful to ask whether the last common ancestor of life had eubacterial or archaebacterial membrane lipids, whether it had one or two bounding membranes, whether it had flagella or not, whether it was photosynthetic or not.

Yet such fundamental questions are typically ignored through preoccupation with sequence trees and genes alone [177]. Genes can survive only by coding for real physical structures of interacting macromolecules, whose evolution can and must be studied by transition analysis of their phenotypes, not just of their gene sequences. Character losses and lateral transfers of a minority of genes from time to time complicate reconstruction of the phenotypes of ancient cellular lineages but do not invalidate it [183,184]; many lateral transfers seem more important for short-term adaptation than for long-term phylogeny, and vertical inheritance subsequently dominates even those genes that were acquired laterally [183,184]. Chemistry of individual proteins is important to life, but the organism must be put back into bacteriology and evolutionary biology, which have recently been dominated by fragmentary gene-, sequence-, and molecule-oriented oversimplifications [1]. Cyanobacteria is an excellent example of a bacterial phylum enduringly defined on organismal grounds a century before sequencing was invented and which retained its organismal integrity, essential physiological properties, and typical cell structure for at least 2.4, and probable over 2.7 billion years despite lateral gene transfer; this conservatism depended on membrane heredity [31,56] – universally vertical in bacteria, DNA heredity (predominantly vertical), and purifying selection. It was not destroyed by either mutation or lateral gene transfer as strong purifying selection can prevent either destroying well-adapted phenotypes; extreme organismal stasis over billenia is a fundamental aspect of life [129]. Even cyanobacterial gene composition and gene order have not been totally mixed, despite diversification into thousands of adaptively specialised but basically similar oxygenic phototrophic strains, and no other physiological types: all cyanobacteria have 181 genes never found in other prokaryotes and some gene order is totally conserved [185]. It can reasonably be argued that lateral gene transfer has been irrelevant to the large-scale evolution and phylogenetic tree of cyanobacteria; this is not to deny the occurrence of lateral gene transfer, especially by cyanophages [186][187,188], but much of this may just be trivial gene replacement of no deeper significance for megaevolution or long-term adaptation than short-term allele jumbling and phylogenetic reticulation by sex in eukaryotes or the replacement of a worm-eaten beam in a historic building by fresh wood from an unrelated tree that does not alter its architecture or function.

It has not been demonstrated that lateral gene transfer is a greater problem (or even as severe a problem) for phylogenetic reconstruction than gene duplications, multiple gene and character losses, convergent evolution, quantum and mosaic evolution [129], and other forms of unequal rates and modes known to confuse the historically dominant vertical phylogenetic signal. All these problems scupper the early naïve expectations by a few biochemists that one could just let the computer make a few sequence trees, using necessarily oversimplified assumptions, and reconstruct the history of life accurately without having to evaluate conflicting evidence, make critical judgements, and weigh disparate data according their mutual congruence (i.e. *a posteriori *rather than *a priori*), as has always been necessary in systematic and evolutionary biology [189,190]. But they do not prevent these tried and tested critically analytic and synthetic methods from unambiguously reconstructing the major features of organismal history.

A recent unduly pessimistic discussion of the logical problems involved in rooting the tree of life [191] suggested that "'transition analysis' of the structural evolution of the cytoplasmic membrane might be helpful"; I have shown here that evolution of the OM is more helpful. However, transition analysis of a single character is insufficient, for transition analysis does not place the root in one fell swoop as protein paralogue trees were mistakenly thought to. It provides a systematic step-by-step approach. Further work along these lines will rigorously test my conclusions. Alternative scenarios can also be tested by evaluating the polarizations of the transitions they imply in a similar way, provided this is done in comparable detail.

A striking conclusion of the present analyses and synthesis is that dramatic evolutionary changes in the cell envelope have dominated bacterial megaevolution. Three major revolutions in bacterial cell surface structure and molecular biology stand out as being especially far-reaching in their impact on the progressive evolution and diversification of life. These are in temporal order: the glycobacterial revolution; the negibacteria-posibacteria transition; and the neomuran revolution. Each dramatically changed the cell surface in ways that profoundly altered future evolutionary potential and the adaptive zones open to the four major groups of bacteria, whose less dramatic evolutionary diversification they punctuate: Eobacteria; glycobacteria; Posibacteria; Archaebacteria. More accurately dating these three revolutions, as well as the origin of life itself, is crucial for interpreting earth history [129]. The last of these revolutions in bacterial surface structure – the neomuran revolution – was what made it possible for phagotrophy to evolve for the first time and thereby create an endoskeleton and endomembrane system that allowed morphological complexity to pervade the whole cell, not just its surface and thus create the eukaryote cell with immensely greater morphogenetic potential [129] – both to make the often immensely complex protists, sometimes with more genes than we have, but also really complex multicells like trees, whales, giant kelp, and toadstools. It is the morphogenetic potential of radically different kinds of membrane and cytoskeletal structures, not DNA and gene regulation, that is the key to understanding biological complexity [129].

In view of their probably most primitive nature among the eobacteria, Chlorobacteria deserve intensive molecular and cell biological study like that devoted to archaebacteria over the past few decades. Study of the biogenesis of their outer membrane is particularly important for understanding early cell evolution and for testing whether it might be derived by simplification from that of other negibacteria, rather than the primitive state as argued here. Characterizing the many lineages known only from environmental rRNA sequences, establishing the full metabolic diversity of the group, and extensive genomic and evolutionary studies to determine whether it is holophyletic or paraphyletic are all essential for finally pinpointing the root of the tree of life.

### Reviewers' comments

#### Reviewer's report 1

John Logsdon, Jr, Department of Biological Sciences, Roy J. Carver Center for Comparative Genomics, University of Iowa, Iowa City, IA 52242 USA

This manuscript represents a complex synthesis of diverse data to address the placement of the root of the tree of all life. The author starts with the premise that molecular trees are insufficient for this task; this is especially true for the paralog-rooted trees on which the currently widely-accepted – indeed, assumed – root placement (between eubacteria and archaebacteria) are based. Instead, the author advocates for using "transition analyses" of complex cellular and molecular characters to provide polarizations that can then be used to infer ancestor-descendant relationships and thus, the root.

In sum, the author provides data and develops arguments to support the position of the root within the eubacteria: specifically it is placed with the Chlorobacteria (green non-sulfur) representing the earliest diverging eubacterial lineage, actinobacteria the latest-diverging eubacteria and archaebacteria derived from actinobacterial ancestors. The central issue here is that eubacteria with two membranes (negibacteria) arose first, with those having only one membrane (gram positives and archaebacteria; unibacteria) evolving from them. This latter argument has been made repeatedly by this author over the years, but this manuscript provides the strongest case to date for this view. The length and detail of the manuscript precludes a complete point-by-point review; my comments instead will focus on a few key points raised by the author. Overall, this manuscript provides a plethora of thought-provoking hypotheses that will undoubtedly be subject to rigorous discussion and evaluation in the light of these and additional emerging data; this is an important paper that should be published in *Biology Direct*.

The abstract indicates that "13 major transitions within eubacteria" are analyzed and extensively discussed here, but only a few of them form the crux of the author's case:

1) Outer membrane biogenesis is so complex as to exclude its origin from unibacterial ancestors; that is, unibacteria must derive from negibacteria.

2) OMP85 homologs cannot be lost; thus, they must have been gained after divergence of Chlorobacteria.

3) The 20S proteasome evolved from HslVU and not the converse; this excludes the root from the "proteates" (Actinobacteria+Archaebacteria+Eukaryota)

4) Flagella arose late during eubacterial evolution from components of two complexes present only in negibacteria (in particular, cyanobacteria); this excludes the origin of flagella from posibacteria.

Of these central points, I find only #3 to be compelling enough to strongly exclude alternatives. *If this inference alone is correct, then the "paralog root" must be incorrect; instead the root necessarily falls within eubacteria*. The author provides a strong detailed argument for #1 here (and previously elsewhere), and he rightfully points out that the alternative has been largely assumed without much careful consideration of any details; however, this does not mean the scenario proposed here is correct. Indeed, it seems that this main idea (negibacterial ancestors of unibacteria) drives the entire hypothesis presented here, when in fact many of the characters considered here can be polarized in opposite directions (including inferred losses vs. gains). In particular, I argue that most of the data presented here can be readily reconciled under a model where the root is alternatively placed within the unibacteria (either within endobacteria or between endobacteria and actinobacteria). In the following figure, I point out the major transitions under these alternative roots. I do not think that either of these posibacterial root positions can be strongly excluded given current data (presented here or elsewhere). Although the author notes these root positions as possibilities in a number of places in the manuscript, I would like to see a more balanced discussion of such scenarios. Even though the author is strongly advocating a negibacterial root here, I wonder if a similarly strong argument could be devised for a posibacterial root placement.

Adaptation of tree shown, noting root positions discussed here (reviewer's figure has been included as Figure 11)

**Figure 10:**
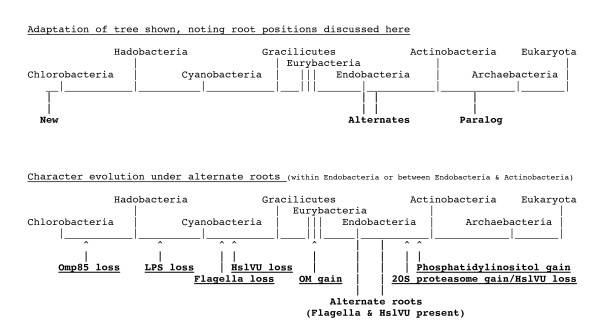


##### Author's response

You correctly emphasize the four most crucial arguments; those are the very four polarizations originally highlighted in the stem of the tree of Fig. 3 by thick rather than thin green bars to emphasize their crucial nature (that figure similarly highlighted the polarizations from prokaryotes to eukaryotes and from eubacteria to neomura that are equally important but were dealt with in detail in my earlier publications). You are right that if 3) is correct, it alone is enough to refute the rooting between neomura and eubacteria drawn from some paralogue trees. Historically I discovered that argument first, which induced me to write the paper. Most of the other arguments were discovered during the course of writing and revision. Number 1 is indeed a development, with additional subarguments, of those I made previously and thus not radically new. However, I consider number 4, which is entirely new, to be the strongest argument to date against the root being within Posibacteria. Before discovering it I seriously considered that the root might be within Posibacteria as you (like Gupta earlier) suggest. However, I find it hard to believe that the similarities of the distal domain of TonB and OmpA can be convergent or that this apparently chimaeric molecule TonB could have first evolved in a posibacterium with no OM and happened by chance to have a distal region perfectly preadapted to be the ancestor of all OM β-barrel proteins that now function only in a membrane that had not yet evolved. Thus I cannot see how evolution could have gone in the opposite direction.

It should be pointed out that many of the author's inferences are based on presence-absence assessments using BLAST analyses of particular proteins and protein families. While such methods are often very useful, they are sometimes not sensitive enough to detect distant homologs and they are wholly insufficient to demonstrate orthology. These caveats, combined with the fact that there exist considerable biases in the phylogenetic representation of bacterial genomes available demand caution be exercised in the interpretation of results. Indeed, given the central importance of Chlorobacteria to this particular hypothesis, a major issue is the dearth of data currently available from them (only 2 complete genomes). The apparent absence of a number of key features (e.g., Omp85, HslVU, flagella) have weighed strongly in the scenario proposed, in which Chlorobacteria are the earliest-diverging eubacteria. Fortunately, ~12 additional genomes are currently underway (according to NCBI). Thus, a more careful evaluation of predictions will soon follow from analyses of additional chlorobacterial genomes.

##### Author's response

Presence or absence arguments are well known to be potentially misleading or ambiguous because absence can be either primitive or the result of secondary loss, which is frequent in evolution for many characters (but never occurs for some). For this reason I have NOT in the present paper actually weighed absence of HslV or flagella strongly in placing Chlorobacteria as the earliest divergers (though I did use absence of both flagella and LPS previously among other reasons to put Eobacteria as a whole near the base previously [1], before I discovered the much stronger Omp85 argument). HslV and flagella have clearly been lost several times during bacterial evolution. But there is no evidence that Omp85 has ever been lost and I have explained why its loss would normally be lethal. It is true that BLAST cannot detect very distant relatives. Thus distant relatives of Omp85 might one day be discovered in Chlorobacteria. If they are, the key question would be: is the great evolutionary distance that has so far prevented their discovery consistent with their being very early diverging from typical Omp85 or the result of secondary evolutionary degeneration? Detailed study of their functions would probably be essential to clarify that. As I have stressed, we do need much more knowledge about their molecular cell biology to evaluate the evolutionary significance of the Chlorobacteria and to test whether their apparently primitive characters really indicate the ancestral state for life or not. But if the Omp85 argument is well founded it does root the tree and enable us to conclude that the absence of flagella in Eobacteria and Cyanobacteria is also probably primitive. I have explicitly recognized that most presence-absence characters (or indels) are not individually useful for polarizing the tree as they can in principle be interpreted as either losses or gains. But given the critical polarizations that do root the tree and give it direction it is useful to plot such other individually less decisive characters on the tree, as I have, and interesting that when one does so the more basal branches turn out to lack a rather large number of such characters found in the more derived groups. This means that the cenancestral cell, even though very complex – much more so than many biologists recognize, had not evolved all the major eubacterial characters; some were later developments, but we could not have predicted a priori which these were; the absence of most of them was NOT used in rooting the tree in the first place.

Suggestions for improvement:

1. The author mainly uses his own nomenclature (some published, some new here) for describing organismal groupings which makes the paper difficult to follow at times. Table 1 defines most of these names and also includes important diagnostic information. However, I suggest that a simpler table without the extra taxonomic information be provided in addition as a "key" to the reader.

##### Author's response

I added a new Table 1 as suggested, retaining the original as Table 2.

2. I suggest the addition of a simple table in which the author lists the "13 major transitions" that are mentioned in the abstract. Indeed, after reading the manuscript it is unclear to me what all of transitions are.

##### Author's response

Instead of adding a new table I improved cross-references in text to Figs 3 and 7, which show the transitions.

### Reviewer's report 2

Purificación López-García, Unité d'Ecologie, Systématique & Evolution, CNRS UMR 8079 Université Paris-Sud, bât. 360, 91405 Orsay Cedex, France

This is a very unusual article due to its length and to the density of its content. Therefore, I will not make an in-depth revision of all the characters and transitions discussed in it, or decorticate all the hypotheses produced, for such an analysis would likely exceed the length of the present article (and perhaps the patience of the reader). I will rather concentrate in a few points that are claimed to be particularly essential for the main objective of this paper as announced in its title, rooting the tree of life, which do not appear to me sufficiently unambiguous as to safely conclude where the root lies.

### Strengths (and a word of caution)

The theoretical base enounced at the beginning of the manuscript to tackle the directionality of evolutionary change and suggest a position for the root of the tree of life appears most reasonable. It proposes a combination, or rather an interpretation, of gene phylogenies with cladistics, transition analysis, congruence testing and critical palaeontology. If rigorously applied, this strategy should provide the best approximation that scientists can dream of to the history of life. However, and unfortunately, there are various problems associated with all of these approaches, including the lack of robust data in various cases, as commented below. Therefore, much caution is needed to avoid biased conclusions and forcing the interpretation of the data (e.g. polarising transitions) to accommodate a pre-formed/assumed existing model. To avoid this, all possibilities should be tested using the same characters and looking for reasonable interpretations to see which one is most parsimonious. Even so, there is no proven rule that evolution follows necessarily the most parsimonious way man can think of, so that congruence among different markers/approaches under careful watchfulness is needed. Knowing that, one essential way to increase the number of meaningful data to place characters on phylogenetic trees is coming back to organismal biology, as is stated in the concluding remarks of the article. I fully agree that this is imperative.

In addition of the above theoretical principle, the present manuscript is a valuable source of evolutionary hypotheses. Most of them require rigorous testing before reaching any positive conclusion, which the author acknowledges in many cases. Some hypotheses may reveal right, others wrong. This does not diminish the merit of their formulation. They are open to scientific validation in the future. However, some of the evolutionary hypotheses that are formulated, which are crucial to the point made is this article, are taken for granted on grounds that are not robust enough, as I discuss below.

### Weaknesses

My major concerns relate to the aspects that I comment as follows.

#### 1) Methods

The author criticises (rightly) many problems associated with gene phylogenies and different artefacts affecting phylogenetic reconstruction that can lead to wrong evolutionary conclusions regarding organismal diversification. However, BLAST (BLASTp) is used here to determine not only the presence or absence of particular protein homologues in different bacteria but also to ascertain their phylogenetic affinity (e.g. high BLAST scores of *Aquifex *P-ring and L-ring proteins with epsilon- and delta-proteobacteria are used to "confirm" the putative position of *Aquifex *within the Proteobacteria; the eukaryotic enzyme UDP-N-acetylglucosamine is suggested to have been inherited from Gram positive bacteria because the domain of a glycosyl transferase that makes the capsular polysaccharide in endobacteria appears among the first BLAST hits, together with one archaeon, but not with alpha-proteobacteria). Nevertheless, we know that BLAST search is affected by even more important problems than tree phylogenies, especially if there are compositional biases due to different types of adaptations. Simple BLASTp does not absolutely ensure the detection of a distant homologue in a given organism. More importantly, BLAST cannot be properly be used to say if a given homologue has been vertically inherited or horizontally acquired. In order to do this, a careful phylogenetic analysis including a good taxonomic representation and appropriate reconstruction methods is required *[see Koski and Golding, 2001, The closest BLAST hit is often not the nearest neighbor, J Mol Evol, 52, 540–542]. *Even so, problems linked to the lack of phylogenetic signal may mask the results. In this sense, BLAST can be indicative but is not demonstrative. Therefore, unless proper phylogenetic analyses are carried out and the results are robust enough, the hypotheses based exclusively in BLAST analyses cannot be confirmed. Whereas these problems are recognised by the author in various places of the manuscript, there is a tendency to use BLAST results as proof in others (or as confirming proof, but if all congruent proofs are as solid as BLAST hits there may be a problem in the conclusion), so I would insist in a more careful formulation of BLAST-based hypotheses.

##### Author's response

I agree that for intermediate degrees of sequence similarity phylogenetic analysis is more reliable than BLAST analysis for establishing detailed relationships. But sequence trees cannot help at all with the problem (that I explicitly acknowledged) that BLAST may fail to detect a genuinely related sequence merely because of extreme sequence divergence; this is because one cannot make trees with such divergent sequences even if one could detect their relationship. For eukaryotic UDP-N-acetylglucosamine transferase there were no BLAST hits whatever with α-proteobacteria, so trees could not possibly help distinguish between my suggestion that the enzyme came from a posibacteria-derived host and the alternative that it came from the α-proteobacterial symbiont. Here BLAST alone is decisive. The point about *Aquifex *is primarily that they have both P-ring and L-ring proteins. High BLAST hits cannot be produced by convergence so they can confirm the presence of a homologue. I agree that trees can be better than BLAST for understanding whether a protein gene was vertically or horizontally acquired. But single-gene trees alone cannot thus discriminate. It is their congruence or otherwise with a much larger body of external evidence that does so. It is not obvious that making trees for P-ring and L-ring proteins would decisively test my assertion that their presence in *Aquifex *in conjunction with a peptidoglycan character supports their classification in Proteobacteria. It is important for critics to note carefully the logic behind my use of BLAST in each specific instance and not just discount them all because for some other evolutionary problems making trees is greatly to be preferred. Nonetheless, I strongly encourage anyone concerned by such questions to make their own trees for any characters where they consider that so doing would improve interpretation.

#### 2) Paleontological evidence

In various parts of the manuscript the author makes affirmations such as "Palaeontology shows that eubacteria are much more ancient than eukaryotes" (abstract), "... palaeontological evidence that eubacteria are much older than eukaryotes..." (introduction: multiple transition analysis...), "morphological fossil evidence that eubacteria are roughly three times older than eukaryotes..." (introduction: the neomuran revolution), etc. According to him, (eu)bacteria would have existed already 2.8 Ga or perhaps 3–3.5 Ga ago, whereas eukaryotes and archaea (archaebacteria) would have evolved only ~800 Ma ago. However, the oldest microbial fossil record is full of uncertainties at present, and does not sustain the above. There are three types of traces to be considered: morphological, organic biomarkers (fossil lipids) and isotopic. Morphologically, except for perhaps cyanobacteria, (eu)bacteria and archaea (archaebacteria) would be indistinguishable in the fossil record. Furthermore, the morphological identification of the most ancient putative fossils at 3.5 Ga as cyanobacteria or as fossils at all is highly discussed [*Brasier et al. 2002, ref. *[158]]. In addition, inorganic structures resembling filamentous organisms can be made purely abiotically *[Garcia-Ruiz et al. 2003, Science 302: 1194]*. This leads to the conclusion that morphology alone cannot be seriously trusted. Cavalier-Smith alludes to the presence of hopanoids, diagnostic lipids for bacteria, in 2.8 Ga-old rocks. If fossil biomarkers are to be trusted, then it should be so also for fossil steranes diagnostic for eukaryotes and fossil polyisoprenoids diagnostic for archaea. Steranes showing alkylation patterns so far characteristic of eukaryotes have been detected in Archaean rocks (Fortescue group, 2.7 Ga) *[Brocks et al. 1999, Science 285: 1033]*. Although they are claimed to be *probably syngenetic*with the rock by Brocks and Summons, later contamination cannot be excluded. Nevertheless, steranes *certainly syngenetic*with the rock have been extracted from ~1.6 Ga-old material at the Barney Creek formation *[Brocks and Summons 2005, in Biogeochemistry, Schlesinger WH ed., Elsevier, pp. 63–115]*. This would be in agreement with morphological evidence suggesting ~1.5 Ga-old acritarchs as eukaryotes [*Javaux et al. 2001, ref. *[146]]. Although most biomarker data likely lie in the hands of oil companies, diagnostic markers for archaea, such as crocetane, have been detected in Proterozoic rocks *[Brocks and Summons 2005, in Biogeochemistry, Schlesinger WH ed., Elsevier, pp. 63–115]*. Unfortunately, some fossil markers, such as phytane, can be derived from bacteria (cyanobacteria), eukaryotes (plants) and archaea (archaeol) and are therefore not diagnostic. Finally, C isotopic evidence suggests that methanotrophy and methanogenesis were present 2.8 Ga ago [*Hayes, 1983, ref. *[149]]. The author mentions it, but he suggests that we need to look for alternative explanations. Why? Although the fossil record can be rigorously criticised, the combination of lipid, isotopic and morphological evidence tends to support, contrary to the author's claim, that archaea and eukaryotes are much older than 800 Ma. Palaeontology does not demonstrate that bacteria are older than any of the other groups. Clearly, more solid data are needed to resolve this issue, but hopefully, improvement in methods and critical analysis of the Archaean fossil record will provide a more clear answer in the near future. Cavalier-Smith's hypothesis that archaea and eukaryotes appeared more or less simultaneously and only ~800 Ma ago will then be confirmed or refuted on a palaeontological basis. In the meanwhile, I suggest that a much more moderate tone be used regarding the fossil record "proofs".

##### Author's response

I agree that interpretation of the fossil record is often problematic. So much so that to discuss the problems in detail would have taken more space than reasonable in a paper devoted to the non-fossil evidence. However, I think the referee is much too dismissive of the morphological evidence and too trusting of the conventional biomarker and isotopic interpretations. It is wrong to argue that morphological evidence cannot be trusted because some is misinterpreted or that all chemical evidence must be trusted if some is. One must also differentiate between the primary evidence and interpretations of it based on other assumptions. One should not uncritically trust or uncritically reject a whole class of evidence but evaluate each specific case and decide the strength of the evidence in question. That I have done. I do not think my interpretations need changing and have added citations to a parallel paper [129] where I discuss these palaeontological matters much more thoroughly and, in particular, present a detailed interpretation of the ultralight 2.78 Gy carbon isotope spike, showing that it could have been produced by an ecosystem with only eubacteria not archaebacteria. Thus it is very unsound evidence for archaebacteria being that ancient. I think you are wrong in saying that the fossil record does not demonstrate that eubacteria are older than eukaryotes. The only evidence ever provided for that assertion, early steranes, is invalid, as several eubacteria can make sterols; regarding the 2.7 Gy cases as eukaryotic is also strongly contradicted by the morphological fossil evidence that does not reveal any definite eukaryotes for the following two billion years [1,129]. If we can't be sure they came from eukaryotes it is irrelevant to discuss whether they are syngenetic or not.

#### 3) Cell membrane

The author emphasises in various places that "the most fundamental question concerning the root of the tree of life is whether the ancestral cell had two bounding membranes (i.e. was a negibacterium) or just one membrane as in archaebacteria and posibacteria" (introduction: multiple transition analyses of complex multimolecular characters can root the tree). According to the author, it is mechanistically difficult to conceive a transition from a single membrane (Gram positives) to two membranes (Gram negative bacteria) (results: membranome evolution). He thus concludes that the transition must have occurred from a two-membrane bounded cenancestor to a single-membrane bounded cell in Gram positive bacteria, and hence, in archaea and eukaryotes which, having a single membrane, would derive from a one-membrane Gram positive stem. We see here that the major argument (together with the proteasome that is discussed below) given to polarise the tree and put the root within the Gram negative bacteria instead of placing it between (eu)bacteria and archaea, as is more generally accepted, depends exclusively on the weight that is given to the two-versus-one membrane boundary compared to the different stereochemistry of the archaeal and (eu)bacterial membranes. Which character is more important, the number of membranes or a different membrane stereochemistry? As Cavalier-Smith acknowledges, although still concluding that this allows polarising the tree, the two-versus-one membrane criterion is not as solid as it appears to be since, for instance, the archaeon *Ignicoccus *has two membranes. Therefore, either *Ignicoccus *derives directly from a two-membrane bounded cenancestor and the rest of archaea lost the outer membrane, which would be compatible with the root of the tree being between (eu)bacteria and archaea, or an outer membrane evolved in a one-membrane archaeal ancestor independently. This invalidates the assumed necessity that evolution proceeded from a two-membrane to a one-membrane state, and therefore, the most fundamental criterion upon which the polarisation of the tree and the proposed root itself are based in this article. By contrast, no single exception is known to the different stereochemistry of archaeal (glycerol-1-phosphate-based ether isoprenoid lipids) and (eu)bacterial (glycerol-3-phosphate-based ester acyl-lipids) membranes. Therefore, the hypothesis that the root of the tree is placed between both, and that they evolved from a cenancestor with heterochiral membranes, is perfectly tenable [*Peretó et al., ref. *[18]]. In this situation and if the transition from (eu)bacterial to archaeal membrane chemistry were evolutionarily impossible, hypotheses sustaining that eukaryotes resulted from a symbiosis between archaea and (eu)bacteria in which (eu)bacteria contributed the membranes would be supported. In the absence of conclusive data, both rooting alternatives (within Gram negative bacteria or between archaea and bacteria) remain possible and hypothetical.

##### Author's response

It is not a question of one character being more important than another. What matters is whether a character difference can be used to polarize a transition or not, the direction of the polarization, and the strength of the argument supporting it. I agree that the lipid difference is important and previously presented a selective argument to polarize the tree from the eubacterial type to the archaebacterial type [1]. The unique stereochemistry of archaebacterial phospholipids does NOT require that the root of the tree is between neomura and eubacteria (as you assume) or that the transition between the two stereochemistries took place at the root itself. If the root is between neomura and eubacteria, as is logically possible and widely assumed (but I argue incorrect), then the ancestral neomuran must either have had both types of lipids or just one. If it had the eubacterial type only, this must have been replaced by the archaebacterial type in the archaebacterial ancestor. If it had the archaebacterial type alone, this must have been replaced by the eubacterial type in the ancestral eukaryote, as Martin and Müller first suggested [176]. If both were present, one type was lost in each ancestor differentially. (I discount the possibility that neither was present and that the ancestral neomuran had a third entirely unknown kind of lipid as science fiction; that it had no lipid at all [107,108] is refuted above and by your own arguments elsewhere [18].) Thus whether the root is in eubacteria or between eubacteria and neomura there had to be at least one replacement of one lipid type by another or losses of two lipid types. No reason has ever been given why replacement of eubacterial/eukaryotic acyl ester lipids would have been mechanistically impossible in the common ancestor of archaebacteria. I have now inserted a refutation of a recent flawed criticism of such replacement [170]. As there is a clear selective advantage for such replacement as an adaptation to hyperthermophily and acidophily, the likely ancestral state for archaebacteria, but NOT for a transition in the reverse direction, this has long a good selective argument for polarizing evolution from acyl ester to isoprenoid ether lipids, not the reverse [1,30]. No argument has yet been given that would clearly polarize the transition in the opposite direction. The referee does not mention my use of flagellar origins to polarize the transition from negibacteria to posibacteria, which contradicts the assumption that the negibacterial envelope was added to a posibacterium rather than lost to generate posibacteria. The *Ignicoccus *case is irrelevant to that polarizing argument, which is logically independent of my assertion of the difficulty of evolving the negibacterial envelope. My paper also makes a careful distinction between merely adding an extra membrane and originating the specific biogenetic mechanisms of the negibacterial envelope, which the above comment overlooks.

#### 4) Proteasome

The second key criterion that is used to polarize the evolutionary transition and place the root of the tree within negibacteria is the distribution of the proteasome core particle. It is present in archaea (20S proteasome), in eukaryotes (26S proteasome) and in some, but not all, actinobacteria (high GC Gram positive). The two, α and β subunits, are distantly related to HslV, a protein widely distributed in bacteria, although absent from cyanobacteria and chlorobacteria (chloroflexi). It is hypothesised that the proteasome derives from HslV, and that its presence in eukaryotes, archaea and some actinobacteria (to the exclusion of early diverging actinobacterial lineages) demonstrates that the root is placed outside a clade eukaryotes + archaea + Gram positive bacteria (those that do not have it would have likely lost it secondarily). However, it has also been claimed that the proteasome in actinobacteria has been acquired by horizontal gene transfer (HGT) [*Gille et al., ref. *[47]]. Cavalier-Smith mentions this in his manuscript although, according to him, the genes are too divergent to safely conclude about their possible acquisition by transfer. Nevertheless, his strongest argument against a possible horizontal acquisition is that "Gille et al. did so [proposed the proteasome acquisition in actinobacteria by HGT] through being unaware of the evidence of a vertical relationship between actinobacteria and neomura and the likelihood that actinobacteria are much older than archaebacteria, making the assumed lateral transfer temporally impossible if it is assumed into their cenancestor (though possibly more likely if it were into the ancestor of Actinomycetales alone)". However, as we discussed before, it is impossible to say from the fossil record whether archaea and the ancestor of the actinobacteria co-existed or not. Consequently, the possibility that they did coexist and exchange genes cannot be excluded. If the proteasome was indeed acquired (together with other archaeal-like genes, see below) by actinobacteria via HGT, the root of the tree would be logically placed outside of a clade formed by archaea + eukaryotes. Therefore, in the absence of a clear answer concerning the origin (vertically inherited or horizontally acquired) of the proteasome in some actinobacteria, this character cannot be used as conclusive evidence to polarize the tree.

##### Author's response

Because of the conceptual possibility of HGT (albeit not demonstrated; a claim not supported by a tree [52] or any explicit reasoning or consideration of realistic alternatives is not evidence) one can reasonably say that the argument is not by itself totally conclusive. That is why rooting the tree requires as any separate polarizations as possible, plus deliberate searches for congruences and incongruences with other evidence – hence the length of this paper. The proteate clade is congruent with other evidence, notably the presence of phosphatidyl inositol in all actinobacteria and all eukaryotes and other less universal characters shared by some actinobacteria and eukaryotes discussed before [1]. I am not aware of any evidence that convincingly contradicts it.

#### 5) HGT vs. vertical inheritance

Both, vertical inheritance and horizontal gene transfer do occur in evolution and must be considered without *a priori *in trying to explain the distribution of genes in genomes. HGT may affect an important number of genes, not just single isolated cases. However, although the author accepts this in some cases (e.g. HGT of genes from hyperthermophilic archaea to *Aquifex *and *Thermotoga *or an important gain of bacterial genes in Methanosarcinales), he dismisses the possibility that a similar situation could have affected Gram positive bacteria, i.e. that archaea transferred an important number of genes to Gram positive bacteria (e.g. glycerol-1-phosphate dehydrogenase or proteasome genes) upon the single criterion that HGT "does not need to be invoked" or is "unparsimonious" as duplications and differential losses could also explain those situations. On the contrary, HGT must be invoked whenever is suspected, but then tested. The same mind openness should be applied to all cases, whether they fit or not our favourite model. Hypotheses on HGT can be tested by proper phylogenetic analyses provided that the involved genes contain enough phylogenetic signal. Hopefully, in-depth phylogenetic analyses and congruence testing will prove or disprove some or many of the hypotheses presented in this article.

##### Author's response

I broadly agree, except that I do not dismiss any possibilities. People tend to consider HGT a possibility when they see a tree that goes against their preconceptions. In such cases one possibility, often insufficiently considered, is that one or more of their preconceptions (e.g. belief in position of the root, or single-gene trees always correctly reconstructing topology) being wrong, rather than HGT, might better explain the apparent incongruence.

#### 6) Calmodulin, phosphatidylinositol, cholesterol

The author sustains that phosphatidylinositol is present in all eukaryotes and actinobacteria but not in other bacteria (results: Unibacteria: links between Posibacteria and Actinobacteria). Similarly, he states that calmodulin-like proteins are only present in Gram positives and eukaryotes but absent from other bacteria (Results: Monophyly of Posibacteria). However, this is not correct. Phosphatidylinositol exists also in myxobacteria *[Benaissa et al 1994, J Bacteriol 176:1390]*. Calmodulin-like proteins exist in a wide variety of bacteria, including, for instance, the alphaproteobacterium *Rhizobium [Xi et al, PNAS 97:11114]*. Furthermore, BLAST searches allow the identification of calmodulin-like proteins in many different bacteria including other proteobacteria or cyanobacteria (not shown). He also says that cholesterol biosynthesis is present in actinobacteria to the exclusion of all other bacteria. However, except for the very last step leading to cholesterol from 7,24-cholestadien-3^β^-ol the whole biosynthetic pathway, as well as all the intermediates, have been identified in myxobacteria *[Bode et al. 2003, Mol. Microbiol. 47:471] *and many other bacteria do synthesise sterols (e.g. methanotrophic proteobacteria, cyanobacteria, planctomycetes). This together with the fact that we ignore the metabolic properties of many bacterial groups (see below) makes imprudent, and to some extent inaccurate, to conclude that the cholesterol pathway is only present in actinobacteria.

##### Author's response

I revised mentions of calmodulin, as related proteins are indeed more widespread in eubacteria than I once thought; BLAST hits are strongest to actinobacteria and cyanobacteria, weak for α-proteobacteria, and not convincing for archaebacteria, making it likely that calmodulin was acquired vertically from a posibacterial ancestor rather than from the mitochondrial slave. However the molecule is so short that trees would be unlikely to clearly discriminate between such vertical inheritance and lateral transfer, e.g. from cyanobacterial food. But my other two points are valid. It is misleading to say that phosphatidylinositol is present in myxobacteria, as the inositol phospholipid of the myxobacterium in question, *Stigmatella*, is not an acyl ester phospholipid as in actinobacteria and eukaryotes but a unique alkyl ether lipid [112], which is not homologous and thus irrelevant to the argument. To prevent other readers being similarly misled by this interesting red herring, I inserted an explanation of it. On present knowledge my statement about cholesterol is also correct, but I agree that the widespread presence of sterols in eubacteria makes it possible that cholesterol may occur more widely than in actinobacteria. However, Summons et al. [192] present convincing evidence that reports of sterols in cyanobacteria may all be mistaken and result from low-level eukaryotic contamination of cultures.

#### 7) Unknown diversity

Cavalier-Smith places a series of characters in different organismal branches and then uses this information to infer the position of the root (close to the Chloroflexi or Chlorobacteria) and also to describe a succession of evolutionary transitions in bacteria. It is a valuable attempt, but it should be also highlighted that it is an approach so far limited to the restricted fraction of prokaryotic lineages for which we have some information. Sequencing 16S rRNA genes from environmental samples has revealed many novel, divergent, clades. The number of bacterial groups equivalent to *phyla *in the Bergey's taxonomy without cultivated and described members exceeds that of those for which metabolic, genomic and structural information is available *[Schloss & Handelsman, 2004, Microbiol Mol Biol Rev 68:686]*. Recent studies on the ultrastructure and metabolism of previously uncultivated prokaryotic groups are revealing novel structural features (e.g. an outer membrane in the archaeon *Ignicoccus*, a nuclear-like membrane in the planctomycete *Gemmata*) or unforeseen metabolic capabilities (e.g. anaerobic methane oxidation by some euryarchaeota). Therefore, even if the hypotheses postulated in this manuscript proved correct, they would not be conclusive until the characteristics of the remaining prokaryotic clades were shown to fit in this scenario.

##### Author's response

I agree that detailed phenotypic and genetic studies of all these apparently deeply diverging clades are important to test my classification and rooting and also because of the possibility that some of them may radically change and improve our picture of bacterial evolution. However deep divergence on rRNA trees can occur for many reasons and does not necessarily indicate radical phenotypic divergence or antiquity. Past experience does not favour the view that most of these, when better known, will really deserve to be treated as separate phyla (many may simply be misplaced divergent representatives of known groups, as suggested before [1,46]), but this does not minimize the importance of characterizing them all properly.

#### 7) Ecological considerations

The author makes a number of ecological considerations that are rather weak. He appears to assimilate primary producers (organisms able to fix inorganic carbon) to photosynthesisers, while ignoring chemolithoautotrophs, which are widespread in several (eu)bacterial and archaeal phyla. I agree that if all Gram positive bacteria have always been heterotrophic, they likely co-existed with primary producers. However, this does not imply that the last cenancestor was photosynthetic. Photosynthesis is likely responsible for most of the primary production on Earth today (although accurate estimates about the deep biosphere are needed), but the first ecosystems might have been based primarily on chemolithoautotrophic organisms, such as methanogens (see comment about palaeontological evidence) or others (sulphur- or hydrogen-based autotrophic metabolisms).

##### Author's response

I think it likely that the first organisms were lithotrophs for energy and heterotrophs for carbon [31]. But I was NOT discussing the first organisms, whose nature cannot be rigorously deduced by cladistic comparisons of extant organisms, but the last common ancestor of all extant ones (cenancestor), which was much more complex and at least somewhat more recent, and whose general nature can in principle be determined by a combination of cladistic and transition analyses of extant lineages. Although I think it most likely that the cenancestor was photosynthetic, I have an open mind. To find out must study Chlorobacteria intensively and establish whether or not the root was within them and if so precisely where. It is very unlikely that methanogens are ancient [1,129], but several kinds of chemolithotroph could be, but I doubt whether most, if any, any preceded photosynthesis. I inserted a brief bit on lithotrophy.

#### 8) Taxonomic rank and nomenclature

Under the evolutionary scheme proposed by Cavalier-Smith, bacteria are a kingdom and eukaryotes are nested within bacteria. Yet, instead of being considered a single phylum, as "archaebacteria" are, eukaryotes are divided in six different kingdoms. How can a taxonomic category contain sub-elements that are ranked at the same initial level? I understand that eukaryotes have a complex morphology and features. However, archaea do possess very different features too. These concern not only the replication, transcription and translation machineries (that appear not very important to the author, although being central to the cell), but also the very nature of the cell membrane (the membrane being a characteristic that appears particularly important for Cavalier-Smith). Yet, archaea are considered a single phylum, sister to the eukaryotic kingdom. Is this not excessively influenced by apparent morphological properties to the exclusion of essential, though less apparent, properties of cells?

##### Author's response

Actually, I divide eukaryotes into only five kingdoms [69], not six. The six 'supergroups' in recent discussions [193,194] that popularize some of my increasing widely accepted phylogenetic interpretations of eukaryotes are clades not kingdoms (one only, Plantae, is a kingdom also; the others are parts of kingdoms or composites of one or more kingdoms and parts of another, e.g. opisthokonts). Many interchange these terms too loosely. On first reading I could not understand the question 'How can a taxonomic category contain sub-elements that are ranked at the same initial level?' A taxonomic category such as phylum normally includes subelements ranked at the same level as each other (e.g. classes), but at a lower level than the parent group (higher category). My classification is consistent with and follows those classical Linnean hierarchical principles. If the referee is worried that sister clades (eukaryotes, archaebacteria) are not equally ranked, or equally subdivided, there is no problem whatever with either; it simply reflects the asymmetry of significant progressive evolution. Eventually after several rereadings, I realised that the referees' question probably relates not specifically to her preceding sentence but to the fact that bacteria are paraphyletic yet treated by me as one kingdom, with five eukaryotic kingdoms derived from it. In essence she is confusing phylogeny and classification, as is so often done nowadays (e.g. in a recent profoundly muddled and conceptually and taxonomically harmful call for the abolition of the name prokaryotes [181]). Cladistically eukaryotes are indeed nested within bacteria (=prokaryotes) on the tree. But taxonomically eukaryotes are not included within bacteria and should never be. They are sharply separated by the hundred or more major differences between bacteria and eukaryotes that originated during the origin of the eukaryotic cell [27,29,129]. Elsewhere [69] I explained these key conceptual differences and why such progressive evolution requires the use of some paraphyletic groups in a comprehensively hierarchical classification of the existing Linnean type, but inserted a few salient points near the end of this paper. Categories like kingdom and phylum refer to taxa, which should always be monophyletic (i.e. holophyletic or paraphyletic), and never polyphyletic, not to clades or grades; thus many taxa are clades but some are necessarily paraphyletic grades because that is how evolution works, e.g. both parent species of descendant allopolyploid species, or the kingdom Bacteria. Provided one understands the important conceptual difference between taxa on the one hand and grades and clades on the other [69], and the radical nature of quantum evolution that is the typical cause of the huge gulfs separating such higher taxa as kingdoms and phyla [129], one can easily see why some kingdoms (Bacteria, Protozoa) must be paraphyletic and others (Animalia, Fungi, Plantae, Chromista) are holophyletic. In cladistic phylogeny one can reasonably speak of eukaryotes being nested within bacteria (lower case, referring to a grade); in evolutionary taxonomy, which takes into account both branching order and the magnitude of phenotypic differences, saying this is wrong; one has to say eukaryotes are derived from the kingdom Bacteria, not nested within it. Ranking is also a complex matter that I discuss in more detail there [69]. But in my view the key factors to be considered are the phenotypic homogeneity/disparity within a group and the magnitude of the phenotypic gulf separating it from relatives. What characters are considered more or less important are properly matters for judgement by the taxonomists establishing taxa and their ranks. Historically ranks change by the usual processes of new discoveries, different workers putting forward alternatives, scientific debate, and eventually usually consensus. The simplifying purposes of taxonomy are best served by keeping the numbers of taxa at higher ranks as small as we reasonably can. The phenotypic disparity within archaebacteria, both structurally and in physiologically important characters, is very much less than for eubacteria, which I divide into nine phyla. As archaebacteria are not obviously more heterogeneous than Posibacteria or Proteobacteria I see no value (and some degree of overcomplicating harm) in dividing them into more than one phylum.

#### Conclusion

In summary, the present manuscript is a substantial source of hypotheses about bacterial evolution for future testing. However, the transitions used to place the root of the tree, namely, the supposed earlier emergence of (eu)bacteria compared to the archaea based on palaeontological evidence, the obligatory and unique passage from a two-membrane ancestor to a one-membrane cell, and the putative vertical inheritance of the proteasome in archaea and eukaryotes from a Gram positive ancestor, are not unambiguously supported by the data presented, as discussed above. Furthermore, since the existing data relative to those transitions are not settled, they could equally support the generally accepted position of the root between archaea and (eu)bacteria. Clearly, determining the directionality of those transitions with certainty requires more confident data and deeper analyses. If the root of the tree cannot be unequivocally placed at present using those transitions, then the rest of the characters and transitions described in the manuscript would point exclusively to the root of the (eu)bacterial tree. In this sense, the possibility that the root of the (eu)bacterial tree lies close to, or within the Chlorobacteria is appealing. I agree with Cavalier-Smith that much attention must be paid to study this group, without forgetting to study the characters of the large bacterial diversity observed by 16S rRNA genes for which no information is available. "Put the organism back into bacteriology" is indeed crucial.

##### Author's response

I note that you offer no objections to either of the two most original new polarizing arguments presented here; the use of flagellum origins to polarize the transition from negibacteria to posibacteria, and of Omp85 to exclude the root from all negibacteria except Chlorobacteria. If they are correct, which the last part of your conclusion tends to concede, the root could be placed between neomura and eubacteria **only **if the unimembranous character of cell surfaces in neomura and Posibacteria are convergent (very unparsimonious) **and **if all the numerous similarities between actinobacteria and neomura are dismissed as convergent or by HGT (also very unparsimonious). The morphological fossil evidence that there are no unambiguously eukaryotic fossils that can reliably be assigned to extant phyla before about 800 My ago [129] should not be so lightly dismissed. Even Knoll [195], who interprets as stem eukaryotes (i.e. those diverging prior to the cenancestor, but now extinct) some earlier fossils that I consider much more likely to be eubacteria, does not claim morphological evidence for stem eukaryotes before 1800–1500 Gy; the earliest date given is only for *Grypania*, which some other palaeontologists, like me, do not accept as eukaryotic since evidence for that assignation is exceedingly weak and trivial; he does not provide any convincing morphological fossil evidence that the eukaryote cenancestor is any older than I stated; the only morphological claim he makes for a specific eukaryote phylum before about 800 My is for *Bangiomorpha*, which he originally identified as a red alga, but I consider to be a cyanobacterium [1,129]; his identification is inconsistent with the Bayesian integration of 18S rRNA sequence data best and multiply highly reliable eukaryotic microfossil dates [36,196]. Morphology provides strong evidence that eukaryotes are much younger than eubacteria, which on any interpretation must be at least as old as the great oxygenation event of 2.45 Gy ago [134], and are probably much older. But these fossil arguments, discussed in detail elsewhere [1,129], are entirely independent of the transition analyses presented here. It is very significant that both these independent lines of evidence are mutually congruent and that both are incongruent with the root being between neomura and eubacteria.

#### Reviewer's report 3

Eric Bapteste, Canadian Institute for Advanced Research and Genome Atlantic, Department of Biochemistry and Molecular Biology, Dalhousie University, Halifax, Nova Scotia, Canada B3H 4H7 (nominated by Simonetta Gribaldo, Unité Biologie Moléculaire du Gène chez les Extremophiles, Institut Pasteur, 25 rue du Dr. Roux, 75724 Paris Cedex 15, France)

##### Preamble to the reader

Before starting my detailed review of this paper, I want to express my personal esteem for T. Cavalier-Smith repeated contributions to the field of evolutionary biology. All along the years, he has often put forward important ideas and original hypotheses that have prompted many debates about the evolution of life, all domains included. Multiple tests of his works have taught us that even if T. Cavalier-Smith's claims were not necessarily right, his thought provocative publications always deserved the consideration of the scientific community. Without a doubt, T. Cavalier-Smith has an impressive biological culture and there is a lot to learn from him. There is also another more special motive to read T. Cavalier-Smith: his very interesting scientific style. Certainly, his global, synthetic, systematic approach often leads him to produce very long papers. Yet, as far as I am concerned, I find such papers fascinating to read, as they exposed not only elaborated and integrated narratives about evolution, but also as they introduce a very original evolutionary epistemology. It is not uninteresting to analyze T. Cavalier-Smith's treatments of his explicit and implicit methodological principles all along a paper, and how, sometimes while he pretends to practice the most solid mainstream hard core science, he is in fact proposing audacious heterodox interpretations. In one words, some parts of the works of this major author are pretty subjective, and to me, this observation is not a criticism. Imaginative efforts of interpretation and in depth analyses are obviously deeply needed in the complex area of evolutionary biology. There are thus many reasons why someone might want to read even the longest T. Cavalier-Smith papers, as they can be fun, enriching and compelling. Yet, because of the level of complexity of those papers where T. Cavalier-Smith pilots us at will, we should always remain cautious before turning into enthusiastic unconditional supporters of his views. The present contribution does not make exception. It is certainly an attractive and stimulating paper, and it will take multiple additional analyses by several specialists to evaluate some of its taxonomical and mechanical hypotheses of evolution. Personally, I remain agnostic on most of the conclusions presented here but I want to encourage numerous readers to test as much as they can this highly interesting version of Cavalier-Smith's Tree of Life, with alternative tools, logic and interpretations.

##### To the author

I totally agree with your opinion that "*an integrative approach, though recently unfashionable, is sorely needed in the face of the mass of new genomic data to suggest biologically well-grounded hypotheses to guide detailed experimental studies in the laboratory*." (P.6, paragraph 4, the primacy of transition analysis), and I support your impressive efforts in this sense. I obviously encourage the publication of your manuscript, but I insist that some revisions could improve its quality and makes it more readable. This is why the rest of my comments are either suggestions, critics or questions that I would be honored you address.

As it presently stand, I feel that the paper contains 3 different papers in one: (i) the rooting of the tree of life by transition analyses, (ii) the proposition/definition of several new clades (such as the gracilicutes, etc.), (iii) the re-affirmation that Archaea are really recent. I think that these three papers, related but different in scope, would benefit from individual submissions and very likely from the expertise of different specialists to be as seriously evaluated as they deserve. I feel that otherwise there is a risk for some of your numerous conclusions to be overlooked by referees, who would like to encourage the publication of the main conclusion of this paper, supposedly about the root of the tree of life, but finally about all the tree of life.

##### Author's response

Although some lesser conclusions, e.g. the clade Gracilicutes, may have less impact than if published separately, I do not favour publishing numerous small fragmentary papers. I prefer to combine them so the reader can appreciate how related topics fit together. This is especially important for the present subject on which so many different considerations necessarily impinge. A fundamental scientific reason why it was important to present the evidence for gracilicute monophyly here, is that it simplifies arguments about placing the root, because they can be considered as a unit rather than as four separate phyla whose relationship to the root must be individually argued (as Bapteste points out below). The same principle applies to every phylum and higher-level group, especially those whose monophyly I discuss in fair detail, e.g. Posibacteria and Eurybacteria. One cannot root the tree by taking every species as a separate unit and has to concentrate on transitions between well-defined large groups. As some groups were not previously sufficiently well defined, and would have been questioned by many microbiologists much more strongly than is now likely with the additional evidence adduced here for their monophyly, providing such evidence was essential. This paper had to do three logically distinct things: (1) better define major monophyletic groups, (2) better establish their correct branching order by cladistic reasoning, and (3) use transition analysis to polarize as many of the transitions between them as possible and thereby establish the position of the root. Thus it is indeed three papers in one, but the first two tasks are essential prerequisites for the third, primary goal of this paper and thus could not have been done later in separate papers. The title refers only to my final goal, rooting the tree, not the two necessary preliminaries; but they are critically important foundations, not peripheral extras. A fourth topic, dating the various prokaryote phyla including archaebacteria, for which palaeontology also is vital, is discussed in much more detail in a parallel paper [129] but needed some consideration here so that the reader can see that transition analysis inferences and palaeontological evidence are congruent, and thus mutually reinforcing.

##### Comments about the existence of a tree of life and its root

I think that your discussion about the Tree of Life, which comes closer to the end of the manuscript than to the beginning, p.105–106, could be more balanced, as you take the existence of a tree as an a priori hypothesis, and you never test it. "*The claim that there may be no real tree of life because of lateral gene transfer *[158]*is profoundly mistaken; all cellular inheritance in prokaryotes is vertical, not horizontal, as cell fusion never occurs except occasionally in Streptomyces. It **is **meaningful to ask whether the last common ancestor of life had eubacterial or archaebacterial membrane lipids, whether it had one or two bounding membranes, whether it had flagella or not, whether it was photosynthetic or not*" is a little too short to summarize a complex debate. This might deserve more explanations. In fact, one can wonder if your conclusion that 13 characters are congruent means that the majority of the metabolic/ultrastructural characters also followed this common vertical backbone? And if not, it questions us on the robustness of the tree presented here and about what will happen when more characters will be added to polarize different part of it. At this topic, you acknowledge that the resolution and clade definition is sensitive to the sampling of characters, and you expose that it is possible that in the future other characters might contradict your present conclusions. Should it be the case, you would change the groups definitions – that renders the paper so long – or you would change the synapomorphies, but you would never really challenge the very idea of a vertical universal structure on which one could map all the characters evolution. Why is it a safe practice? Could you comment a little bit more on your tree-like perspective in the MS?

From my narrow phylogenic perspective, it is presently unclear to me that character evolution has to follow a tree-like pattern for all the organisms. There are certainly some cases of lateral gene transfers, and differential losses that can be misleading. You as well mentioned some complex situations (p.6)" *with congruence testing a serious mistake in one part of the tree may be revealed by incongruence with other parts. If two polarizations in different parts of the tree are incongruent (contradictory), then either the topology of the tree is incorrect or one of the polarizations is incorrect, and the source of the conflict can be sought for and at least one of the interpretations corrected in the light of the overall evidence from as many sources as possible*". You claim that your characters are immune to this problem, but is there any formal way/test to make us confident about which characters did not undergo an incongruent evolution and which ones did?

##### Author's response

The issue here relates to lateral (or horizontal) gene transfer, which has been important in bacterial evolution. However, cell inheritance is always strictly vertical in bacteria, much more so than in eukaryotes where sexual cell fusion makes even cell inheritance reticulate on the microevolutionary scale, and thus not tree-like. On the largest scale the rare instances of intracellular cell enslavement (symbiogenesis) mean that large branches have occasionally fused long after they diverged (as in the origin of mitochondria), but such cell enslavement apparently never occurred in bacteria. Thus all prokaryote evolution has been in the context of repeated growth and division and exclusively vertical inheritance of whole cells composed of membranes, genomes, cell skeleton, and a network of catalysts including ribosomes and enzymes. Against this historical dominance of universal verticality of cell lineages repeated instances of lateral transfer of genes have substituted foreign but broadly functionally equivalent versions of some enzymes and also added others that are novel to a lineage, having first evolved elsewhere. In real trees branches can fuse, so it is a philosophical issue, rather than a practical one, whether it is appropriate to call this composite pattern tree-like or not. Nobody denies that both vertical and horizontal inheritance occur, so it is not helpful to discuss this issue at more length here. There certainly is a 'vertical backbone' (to use your phrase) to bacterial evolution set by successive cell cycles and the vertical transmission of all cellular characters. Asking whether most characters followed this backbone or not is somewhat oversimplified. This is because a character like rRNA can follow that pattern almost exclusively and yet very occasionally be transmitted laterally by gene transfer. Even if most genes have at least sometimes been subject to lateral transfer it would be wrong to imply that this is the dominant mode of inheritance. Probably for most genes most of the time inheritance is vertical. This paper is concerned with the evolution of major characters that can be used to define bacterial phyla and subkingdoms and in determining their evolutionary relationships, i.e. with organismal evolution, not the evolution of individual genes. These major characters are essentially all multigenic, and often complex ultrastructural ones, for which there is no evidence that lateral transfer has been significantly involved in their evolution. I cannot stress too strongly the vertical inheritance of membranes, cell skeletons and whole metabolic pathways. It is a basic fallacy to regard bacteria or eukaryotes as being simply the product of their genes and genomes. Genes can do nothing outside this structural framework. It is not clear even in theory how lateral gene transfer could cause a structural innovation like the origin of thylakoids or the addition or loss of an outer membrane. To use the word phylogeny to apply just to gene sequence trees is, as you candidly admit, a too narrow interpretation. One must consider structures and morphogenesis as well as genes in phylogeny. These considerations are a key to establishing the vertical cellular framework as securely as possible, which will allow us to assess more properly the modifying impact of lateral gene transfer, which appears to be of very variable quantitative significance in different parts of the tree and among different types of characters, and is not a universal obstacle to reconstructing large scale organismal history as is sometimes exaggeratedly claimed. With respect to congruence or incongruence of polarizations, I claim only that the polarizations I deduced are mutually congruent, and if correct require the root to be where stated. For any other position one or several of my polarizations must be shown to be wrong. Their number is so small we do not need formal methods to test their mutual congruence. The issue is simple; each of my polarizations is either right or wrong. If in future any other characters are discovered to give polarizations incompatible with my conclusions, we must examine precisely which specific polarizations appear mutually incompatible and then work out which alternative is more solidly based, using every piece of evidence and line of reasoning available to us. Because the way to resolve such purely hypothetical conflict must be case-specific, I cannot now suggest precisely how to do it. The situation might never arise, as strongly contradictory character polarizations might not exist, but additional characters that can be strongly polarized should be sought, for the more we have that are mutually compatible the more confident we can be of the conclusion they yield.

You wrote "*Figure *2* emphasizes that the most fundamental question concerning the root of the tree of life is whether the ancestral cell had two bounding membranes (i.e. was a negibacterium, as argued here) or just one membrane as in archaebacteria and posibacteria." *This might indeed be an important question. However to me the most fundamental question concerning the root of the TOL is: "is there a single last common ancestor bearing all the characters investigated here or could some of the defining features have been carried by different individuals of contemporary populations?" Do you have any way in your methods to test that there is indeed a last common ancestor and not multiple contemporary ancient populations, possibly exchanging DNA at that time or responsible for the evolution of some feature used here? I am not referring to poorly shaped progenotes there, as your last common ancestor seemed itself fairly evolved. In fact, could an organism such as the last ancestor of all life, "*a non-flagellate, non-spore-forming, eubacterium with acyl-ester membrane lipids, a murein peptidoglycan wall, and fully developed eubacterial molecular biology and cell division mechanisms*"p.101, have really been alone on Earth, immune to any character exchanges with contemporaries? If not, why would we like to trace back all the living beings characters to such a single root?

##### Author's response

There probably were 'multiple contemporary populations' at the time of the cenancestor. But that does not mean that some had two membranes and some one! I should be surprised if they were not exchanging DNA at all, if only by viruses. Nonetheless all modern life could have descended (and probably did if cell fusion did not then occur) from just one of those cells and it is the properties of that one that is inferred by phylogenetic reconstruction of the cenancestor. We cannot hope to reconstruct every detail, nor say anything about contemporary forms that became extinct. To assert that the cenancestor had the few characters specified is NOT to assert that no gene transfer was then occurring for 'any characters'.

##### Comments about the transition analysis methodology in general

It might be interesting/helpful to clarify for the readers the conditions under which your approach will succeed, but also the conditions under which your methods would fail. I imagine lateral gene transfers, a common ancestor lacking any of the characters used in the transition analyses, a too large number of ancient phyla to be resolved by a few synapormorphies, might be problematic. Considering these conditions, do we know if the use of the methodology is safe as such a deep level as the root of the TOL?

##### Author's response

Obviously, as I stressed, some simple transitions seem in principle to be possible in either direction and are not helpful for polarization. Also obviously, we cannot expect to find polarizable transitions in every part of the tree. But fortunately we do not need to polarize every branch of the tree and fully resolve it to every cell generation that has occurred in four billion years. What we care about is accurately resolving the big picture, not reconstructing every trivial detail. This answers the too many phyla question. Taxonomists actually define phyla by choosing big structural differences about which we care. This limits their number. Just looking at sequence trees has led some to use 'phylum' too loosely in an unhelpful way and refer to far too many bacterial 'phyla' to be useful. I think the 10 in Table 2 are enough given present knowledge, but future discoveries might require more. 'A common ancestor lacking any of the characters used in the transition analyses' is simply refuted. Logically the cenancestor must have had either one or two membranes, or either peptidoglycan or not or flagella or not, or lipopolysaccharide or not. Lateral transfer of a character could confuse cladistic grouping of taxa by shared derived characters (for example of Actinomycetales with neomura, if proteasomes were transferred by transfer of genes from archaebacteria to the ancestral actinomycete), but it would not confuse the polarization of the direction of evolution (e.g. from HslV to proteasome rather than the reverse) as it is essentially irrelevant to the reasoning involved.

##### Comments about the numbers and the definitions of the clades

A limited number of characters can only resolve a tree if the numbers of clades is itself limited. We seemed to be in such a favorable situation here, but how can we be sure that the 10 clades you retained are correct/acceptable, independently of the character you used to polarize the tree? Are not we lacking some independent evidence at this topic? I see how some clades take form as the outcome of your transition analyses (i.e the monophyly of Posibacteria, the location of Fusobacterium in Eurybacteria, the Glidobacteria), but don't you think that some clades could/should be more thoroughly established independently of these characters or a priori? If most of your clades are not defined independently of the characters used to postulate the root of the tree, then maybe more characters could just dramatically challenge your conclusions. Could you please comment on that, so that it does not seem that you are forging the groups that are matching your conclusions, instead of, for instance, being unable to resolve the problem. You could maybe summarize in a table further but independent evidence in favor of each of your clades and recall the results of phylogenetic trees of core markers about these groups? Are they for instance recovered in the paper of Cicarelli et al. published in Science, vol. 311, on March the 3^rd^, 2006? (I do not like this paper but maybe it could help supporting some of your claims). Should these trees be non existent, they could then be reconstructed by anyone who would like to test your propositions. I feel that this proposition might allow you to shorten your text a lot (see below), and thus encourage more people to discover its message.

##### Author's response

Actually the core of Posibacteria was defined as a taxon (as Firmicutes or earlier informally Gram-positive bacteria) decades before anyone asked whether the first cells had one or two membranes, so your assumption is historically incorrect. Furthermore none of the three groups you mention are clades. Posibacteria, Eurybacteria and Glidobacteria are all paraphyletic grades, but nonetheless good taxa. You cannot and should not attempt to establish clades a priori. But seeking independent evidence for relationships is desirable. **But the key polarizations are independent of the uncertainties you mention. **Thus, for example, the logic of polarization from negibacteria to posibacteria is independent of whether Posibacteria are paraphyletic or polyphyletic: my arguments show that neither endobacteria nor actinobacteria can be ancestral to negibacteria as both have the same type of flagellum and neither has either of the two probable precursors of flagella. Thus both must be derived from negibacteria, irrespective of whether the OM was lost by their common ancestor, as I argue, or twice independently. Whether eurybacteria are paraphyletic or polyphyletic also does not affect the Omp85 argument that excludes all of their sublineages from the base of the tree. Likewise for glidobacteria. **Thus none of the key polarizations that led to my rooting is affected by the particular uncertainties you mention. **The Ciccarelli 31 protein 191 species tree [175] shows archaebacteria as holophyletic sisters to eukaryotes (exactly my tree, and contradictory to common assumptions that archaebacteria are ancestral to and much older than eukaryotes, e.g. [176]) and Endobacteria as sisters to neomura with high bootstrap support (80–100%) but places Actinobacteria among negibacteria as sisters of Planctomycetes and Spirochaetes (with very low support and contrary to my tree). All other relations of major groups on their tree have only moderate support. Of my 10 bacterial phyla one (Chlorobacteria) is represented by only one sequence and five are clearly monophyletic (Archaebacteria, Spirochaetes, Hadobacteria, Cyanobacteria with strong support, and Sphingobacteria with moderate (40–80%) support). Planctobacteria appear polyphyletic with Chlamydiae and Planctomycetes not grouping together; but the rearrangement needed to group them would cross only very weakly supported branches and both are grouped with Sphingobacteria and Spirochaetes, as in my tree, but also with Actinobacteria, (contrary to my system) with moderate support. Interestingly, this tree also places Acidobacteria (widely treated as a separate group because of poor 16S rRNA resolution) within Proteobacteria (with very strong support (98%), as sisters to Deltabacteria) as I first proposed [1]. Thus Proteobacteria in my broader sense than usual [1] is monophyletic except for the exclusion of *Aquifex*, which groups instead with *Thermotoga*, with strong support (though the authors recognize this could be hyperthermophilic bias as argued here). Interestingly this hyperthermophilic branch has only weak support for its unresolved position within negibacteria; but it is within Negibacteria and there is strong bootstrap support for excluding it from the neomuran/Endobacteria clade, contrary to 16S rRNA that tends to group them with neomura. Thus only three of my phyla are not shown as monophyletic: Proteobacteria, solely because of the exclusion of *Aquifex*, Posibacteria because Actinobacteria do not group with Endobacteria (but bootstrap support is only moderate (40%–80%, and Actinobacteria would not need to cross a highly supported branch to join them), and Eurybacteria. This 31-protein tree is more concordant with my analysis than any other multigene tree I have seen, and is very much better than single-gene (e.g. 16S rRNA) trees in its concordance with organismal characters; the only taxon that would need to be moved across a strongly supported branch to make it concordant with my tree is *Aquifex*. Moving *Aquifex *to Proteobacteria would also make Eurybacteria (represented only by *Thermotoga *and *Fusobacterium*) monophyletic. Thus only three branches need be moved to make all 10 of my phyla monophyletic (the thermophilic *Aquifex *across one with strong support, the GC-rich and thus possibly biased Actinobacteria across a moderately supported one and Chlamydiae across weakly supported branches only). Actinobacteria and Proteobacteria would need to be moved across only moderately supported branches to make both Gracilicutes and Posibacteria monophyletic. Even Glidobacteria and Eobacteria are monophyletic on this tree, but with weak support. The remarkably high concordance of this 31-protein tree and my groupings by ultrastructure and molecular cladistic characters, entirely independent evidence, strongly suggests that both are close to the truth; the multigene tree would probably be further improved by having more representatives of weakly sampled taxa, but 31 proteins are probably not enough for complete resolution. Ideally the number of proteins and number of taxa need to be at least doubled. With respect to eukaryotes that tree is good in having monophyletic fungi, animals, opisthokonts, unikonts, Plantae, and chromalveolates, but wrong (because slightly misrooted) in showing paraphyletic bikonts and excavates, but the latter are very poorly sampled. Finally, the conclusion by Ciccarelli et al. [175] that their tree supports a Gram-positive ancestry of eubacteria is wrong, because it is based on the mistaken assumption (nothing to do with their tree, which is necessarily unrooted) that the root is between eubacteria and neomura. If the root is, as shown here, beside or within Chlorobacteria, their tree supports instead the Gram-positive origin of neomura, as I first proposed [29], and contradicts the widespread assumption that *Thermotoga *and *Aquifex *are deeply diverging eubacteria or have any particular relationship to archaebacteria.

##### Comments about the transition analysis of a single character versus the transition analyses of multiple characters simultaneously

The congruence between transition analyses is a both a key and, with all the respect I have for you, a blind spot of your methodology, at least the way it is currently written... I do not want to seem negative: what I mean is that I wonder if there are not multiple equiparcimonious scenarios of transition analysis when we look at data character by character. I am sure you considered that, and identify these possibilities. For instance, you might have deduced that character 1 was compatible with two stories A and B, but as the TS analysis of character 2 was in favor of the story A, you might have retained the story A as the correct/congruent scenario for both characters. For instance, the evolution of the flagella requires a certain condition of the membrane, you said, before proposing a congruent scenario that would explain how these two features evolved. Intermediate results of your analyses, not only the conclusions, could be important as well for the readers. Maybe, you could resume the different equiparcimonious scenarios in a table character by character, so that anyone could check which are the almost congruent alternative scenarios for the root, if there are some. Furthermore, other researchers could then use the full extent of your work to add their own new characters. Maybe, when they will add new data, the congruence between transition analyses of multiple characters may then favor a different but equiparcimonious scenario for the character 1 (i.e. maybe now, story B will be the one to use to build the most congruent scenario). Proposing these intermediate considerations would finally convince anyone that you envisaged and tested (exhaustively) or many possibilities and would help not to overlook alternative rooting propositions.

##### Author's response

There are some indels that partition the tree into two parts, but which do not polarize the direction (i.e. they could have been insertions or deletions); some character gains could also be interpreted as losses. I have already been careful to distinguish between such characters that can in principle be interpreted in either direction and those where I think direction can be inferred (and gave my judgement about the relative strengths of these inferences). I have NOT presented as strongly polarizing any characters that I think can be equally parsimoniously interpreted in the opposite direction. I had already discussed the implications of placing the root in other places, e.g. between neomura and eubacteria, between proteates and the rest, between Posibacteria and the rest, within Gracilicutes. As the number of currently polarizable transitions is not large, a critical reader can readily take the tree of Fig. 7 and reroot it as they wish in yet other places and easily see which polarizations discussed here have to be reversed in each case. They can similarly switch any branches where they consider I have the topology wrong and explore its implications. To answer your question about how my ideas changed as my research progressed, when I had only the neomuran and proteasome transition arguments, before discovering the flagellum origin and Omp85 polarizations, I took the possibility of a root within Posibacteria very much more seriously than I now do. Before I came across the sortase data I was less confident in the monophyly of Posibacteria. I have considered all possible positions of the root between the taxa on Fig. 7 and the possibility that it might be within any of them, and reduced the possibilities that seemed defensible to just two: beside or within Chlorobacteria. I also considered several variants for the eubacterial topology, but not exhaustively, as I think the evidence for a bipartition between Gracilicutes and the rest to be very strong. You will notice that Fig. 7 differs from my previous tree [1] in placing cyanobacteria below the eurybacteria/gracilicute dichotomy, rather than as sisters to eurybacteria; this requires testing more rigorously by stronger characters if they can be found. So does the relative position of cyanobacteria and Hadobacteria, which still lacks a really strongly polarizing character; their interchange would require different assumptions about which characters were differentially lost or gained between them, but would not change the root position. If correctly rooted, a 31-protein tree published while my paper was being reviewed [175] shows the same topology as Fig. 7 for Glidobacteria, but with weak support.

##### Comment about the bounding membranes

In "Multiple transition analyses of complex multimolecular characters can root the tree, paragraph 1", you wrote "*Figure *2* emphasizes that the most fundamental question concerning the root of the tree of life is whether the ancestral cell had two bounding membranes (i.e. was a negibacterium, as argued here) or just one membrane as in archaebacteria and posibacteria (collectively therefore called unibacteria *[1]), *as has traditionally been widely assumed*." Could you comment on Koonin EV, Martin W. (On the origin of genomes and cells within inorganic compartments. Trends Genet. 2005 Dec;21(12):647–54. Epub 2005 Oct 11) who offered an alternative to this view?

##### Author's response

That paper has all the same fundamental flaws as Martin and Russell's [98] on which it is based and which this paper already criticized. First they assume that the root is between neomura and eubacteria, ignoring all earlier arguments to the contrary [1], and which the transition analyses presented here and the palaeontological evidence detailed in [129] very strongly refute. Second they assume that the cenancestor had no developed lipid membrane and that the cenancestor consisted essentially of genes and proteins enclosed in a honeycomb of inorganic compartments, but free to move among them! That is seriously incompatible with all we know of cell biology and the numerous characters shared by neomura and eubacteria that convincingly show that the cenancestor was a complex cell with at least one bounding lipid membrane (I gave four strong arguments for this in the present paper) and at least a thousand genes. Thirdly they assume that DNA replication evolved independently in eubacteria, also very non-parsimonious compared with my interpretation of a eubacterial-neomuran transition with rapid quantum evolution in DNA handling enzymes caused by histones replacing DNA gyrase [1]. Finally the postulated inorganic compartments could not have been capable of multiplication by growth and division as discrete units on which natural selection could act individually, and whose multiplication could be modulated by gene-coded proteins in the way that lipid membranes can. Such organismal properties conferred by a mutualistic symbiosis between lipid membranes, genes, ribosomes and proteins was essential for evolution to progress beyond the level of competing selfish genes to the level of complexity that can reasonably be inferred by cladistic arguments for the cenancestor [31]. The untenable thesis of their paper is an outstanding example of the consequences of taking insufficient note of cell biology and palaeontology, and the actual mechanisms of progressive evolution, and focusing almost solely on genes and enzymes and a narrowly uniformist interpretation (basically mistaken, see [129]) of sequence data. Their criticisms of the actinobacterial ancestry of archaebacteria are fallacious, as explained in new sections of this paper.

##### Some arguable claims

In "Multiple transition analyses of complex multimolecular characters can root the tree, paragraph 2, p.13", you mentioned that "*bayesian relaxed molecular clock analyses calibrated by multiple palaeontological dates for 143 proteins *[31]*and for 18S rRNA *[32]*suggest that the eukaryote cenancestor was only about 1.1 Gy old, whereas the fossil record indicates that eubacteria are at least 2.8 and probably about 3.5 Gy old *[1,33]." To my knowledge, Hug and Roger are about to publish a paper suggesting that this molecular dating of eukaryotes, among other, is not convincing. Since it is fairly accepted that molecular dating is indeed not satisfactory enough, you might be willing to tone down the claim based on such references. I think the age of the origin of eukaryotes is not settled so far, at least by phylogeny.

##### Author's response

Roger and Hug [35] indeed reanalyzed those very data by a technically superior method estimating parameters for each gene separately, not concatenating them all; this yields an even younger date of ~900 My (not 1100 My as in [34]) for the eukaryote cenancestor – strictly speaking the divergence between Amoebozoa (the phylum with the oldest fossils I accept as indubitably eukaryotic) and all other eukaryotes, which is probably essentially the same given the lack of resolution at the base of the tree. They also analyse other smaller data sets by a variety of methods that give somewhat earlier dates, but none as old as eubacteria. Despite the problems they carefully discuss [35], the best analyses (see also [196]) clearly support the cenancestral eukaryote as **much **younger than eubacteria. Furthermore, the compelling evidence that the enslavement of an α-proteobacterium preceded the cenancestral eukaryote proves that α-proteobacteria (eubacteria) are older than eukaryotes. Unless you were to root the tree of life between α-proteobacteria and all other organisms (which would entail neomura being derived from eubacteria, the central point of my argument anyway), the prokaryote tree proves equally strongly that the cenancestral eubacterium is much older than α-proteobacteria. These two compelling deductions together mean that trees alone make it impossible to maintain both that the root is between neomura and eubacteria and that eukaryotes are anywhere near as old as eubacteria. Thus you must accept either or both of the statements that 'eukaryotes are much younger than eubacteria' or that 'the universal root is in eubacteria'. Both cannot be false. I think both are true.

##### Comment on scenarios based on complexification

For instance: In "HslV to proteasome differentiation polarizes the evolutionary transition, p.19, 1 paragraph"

I use this ("*I argue here that the proteasome 20S core particle evolved from the simpler HslV, not the reverse*.") as a starting point for my general questions and as a potential position in the text to "anchor" the following comments.

Your arguments are often pretty compelling, but could you ensure the reader that you dealt with characters for which the complexification was indeed constant along evolution, and not where complexification and simplification alternated due to changes in organisms way of life. Could you ensure that two different lines of descent were not actually existing, which evolved differentially from an intermediate common ancestor, one line symplifying and the other one complexifying? In this last case, the root should be in between the two lines and not within the simplest one.

##### Author's response

I do not rule this out. One obvious example is the divergence of archaebacteria and eubacteria from their neomuran cenancestor. Clearly eukaryotes became far more complex. By contrast archaebacteria can reasonably be inferred to have lost at least scores of genes shared by eubacteria and eukaryotes [1], and I think more likely around a thousand genes [108], during adaptation of their cenancestor to hyperthermophily.

I wonder this, since you notably wrote: "*It is well known that evolution can involve simplification as well as stepwise increases in complexity*.*Therefore, the fact that one can see functional advantages in the proposed increase of complexity from smaller and simpler HslV to larger and more complex 20S proteasomes, though adaptively much more plausible than evolution in the reverse direction, for which no selective advantage is apparent, is not in itself proof that evolution occurred in that direction.*", p.21. This is fair enough, but it also underlines how the transition analyses scenarios can be somehow fragile and dependent from our imagination to conceive selective advantages. Practically, in most of the case, but for the double-membranes of negibacteria, considered primitive with respect to the simple membrane of posibacteria, you seemed to decide that the less complex stage is the one involving a more limited structural diversity in terms of components. How did you rule out – if you did – that having a simpler molecular organization could not actually correspond to a refinement, an optimization going away from a more complex/heavy structure? If there is no reason that there should be a unique tendency in evolution (i.e. toward complexification all along the TOL from the root), should we expect that living beings would experiment different adaptations?

In the case of the HslV, which selective advantages were brought in by the addition of alpha and beta subunits, each with partitioned functions only? Would not it be conceivably potentially advantageous to loose such partitioned subunits, little operational on their own, for the economy of the cell? Is it that counter-selected to use less proteins with fragmentary functions to make its proteasome than to use more of these proteins? Among other different motives, you expose as a rebuttal to this kind of reverse scenario that: *"arguing that HslVU evolved from proteasomes would leave totally unanswered how 20S proteasomes evolved. If HslV were not the ancestor of the a and beta-subunits, what is? *" p.23. You are right, but why should this ancestral candidate still be around in today's organisms, especially if the tendency was to simplify the old structures and arguably the proteins performing the ancestral task were less efficient? Could you tell us more about that: is there a risk to polarize the data with a bias toward complexification or is there indeed such a genuine evolutionary tendency in prokaryotes?

##### Author's response

Yes there is such a risk; such mistakes have often been made. The risk is greatest when there is a total loss of a complex character and no trace of it having ever been present. Cilia, flagella, photosynthesis, chloroplasts, peptidoglycan, and numerous individual enzymes have been lost many times each; this alerts us to the necessity to decide in each case whether loss or gain occurred. But some characters seem on present evidence never to have been lost, e.g. ribosomes, mitochondrial membranes, proteasomes within neomura. Thus simplification by loss, though common, is not universal for all characters and should not be assumed without phylogenetic evidence. Thus for characters like TolC and TolB their presence in all phyla of negibacteria except Hadobacteria and Chlorobacteria is most parsimoniously interpreted as reflecting their origin after these two phyla diverged from the rest. But in itself this is not strong evidence for their being close to the root, because of the possibility of loss. But since the possibility of loss is immensely lower for Omp85, where loss is lethal, its absence from Chlorobacteria is much more likely to be ancestral than by loss. Indirectly, this gives us increased confidence that the absence of TolC and TolB from Chlorobacteria is the primitive state. However, simplification by total loss differs profoundly from simplification of a multigene macromolecular complex by a reduction in the number of different subunits and merging their separate functions into a single one no larger than either, which would have to be supposed if HslV actually evolved from 20S core proteasomes rather than the reverse. I am not aware of **any **examples where comparable simplifications of a **differentiated multiprotein macromolecular complex **have actually been shown (with solid phylogenetic evidence) to have occurred. I am not referring to the substitution of a simpler assembly for a more complex one, e.g. of RuBisCO II in dinoflagellates for the more complex RuBisCO I [197], but the actual transformation of one macromolecular assembly into a simpler homologous one. If you or any reader can give me examples, please do. Possibly such simplification never occurs. It should not be assumed without evidence or precedent. I had already mentioned that expansion of the digestion chamber volume and distancing the active centre from the exterior to reduce random digestion of the wrong substrate is a plausible selective advantage for the transition from HslV to proteasome rather than the reverse for which no advantage is obvious, and which is likely to be harmful. As there is no evidence that 20S proteasomes have ever been lost by free-living organisms, this implies that they are a very substantial advance on HslV, the patchy distribution of which among negibacteria suggests several losses – perhaps you might attribute it solely to lateral gene transfer! This is a case where sequence phylogeny might help, but it sometimes yields trees where it is very hard to distinguish between multiple losses and multiple transfers. Because HslV loss seems relatively easy, I give its absence from Chlorobacteria almost no weight in rooting the tree, but as it had to evolve somewhere, and the other evidence puts the root there, it is simpler to assume that they never evolved it rather than lost it. The question why should ancestral types still be around if derived types are more efficient applies right across the tree of life, and is not specific to proteasomes. In general the answer must be that the derived type is better at doing slightly different things, but the old type is better in other niches; i.e. new adaptive zones and niches are being created/colonized. There were still niches for mesophiles after archaebacteria became more efficient as hyperthermophiles. There were still niches for bacteria after eukaryotes evolved, for fish and algae after tetrapods and land plants evolved, for reptiles after birds and mammals evolved, for pteridophytes after seed plants evolved and so on. The derived state is better in some ways and less good in others. Remember also that an organism is a mosaic of characters and that some have advantages in some niches and disadvantages in others. Evolutionary progress is not a matter of improving every thing equally. If it were there would be only one species, not 10 million. Probably all major transitions discussed here took place after there were at least thousands of 'species' (I think bacteria have no real species). In each case the transition took place in only one of them. So the new type was in most direct competition only with its immediately ancestral, most similar type; this may well have gone extinct through competition, but there is no reason why this should happen to all the other thousands; it might to some. Generally in a major transition it is the intermediate stages along the direct ancestral line that will be thus extinguished, because they are less efficient at the **same **thing than their direct, more improved, descendants. This explains why intermediates in quantum evolutionary changes never survive, yet more distant primitive types often do so. The forgoing is the fundamental explanation of the major gaps among higher taxa that worry creationists, but allow clear-cut higher taxonomy as in Table 2, and why evolution results in both net progress and increased diversity. If something starts simply, as life did, there is an inevitable tendency towards complexification and differentiation (true in astronomy and geology too; even the earth's inorganic crust has become more differentiated and structurally complex over time); but complexification will not be evenly spread across the tree, but can go into reverse locally. Even random diffusion from a centre has inevitable net direction until all becomes homogeneous. But evolution never produces homogeneity and is thus more strongly directional. Evolution does not work by broad general steady progressive change that displaces all phylogenetically earlier types, as in Lamarck's transformism. It is more individualistic and differentiated. Our explanations of it must therefore be more sophisticated than naïve uniformist transformism [131], a hint of which is detectable in your question.

##### Comments about the recency of Archaea

I have some concern with this part, as I think you should try to publish it as a separate paper, facing the possible objections of specialists of Archaea to see if they agree or not with this interesting idea. Personally, I have no preconceptions to locate Archaea close/far away from the base of the tree, but I am not convinced the arguments is truly needed here. In fact, you do not need to claim such a recency of archaea to root your tree in the Glidobacteria, you simply need to tell that Archaea are derived. How derived is not so much the problem for the root, as in doing so you complexify the paper by climbing up the tree again, instead of going down.

This being said, on some of your figures Archaea derived from paraphyletic actinobacteria (fig. 5, 7), while in another one they are sister group of those actinobacteria (fig 3). I feel that, in terms of recency, it might make a big difference if they are sister-group to Actinobacteria, or even at the base of the Unibacteria. Based on figure 7, I do not really see which synapomorphies constrained the Archaea to be higher in your tree than at the bottom of the Unibacteria, if we adopt your current polarisation and synapomorphies for Unibacteria, and why your neomuran revolution has too be that recent. I have thus a few naïve questions. Would not it be appealing to put the three revolutions you evoked closer by simply rearranging the order of emergence of your Unibacteria? Having slightly more early emerging Archaea than what you claim, would lead to the suggestion that the neomuran revolution was directly triggered by the loss of the OM, a consequence of the membranome revolution. And indeed, you argued that there must have been some important consequences of the unexpected increase in thickness of the medium murein sacculus of the negibacterial membrane. What prevent us to consider that following this unique membrane transformation due to some local environmental changes or mutation, a great period of instability in the morphology of prokaryotes was initiated? This would be the case if Archaea were not that recent. After all, some indirect evidence might suggest that the archaeal fossil record could be as old as 2.8 billion years, and I am aware of presently unpublished phylogenetic evidence in favor of a proximity between the certainly old cyanobacteria and Archaea. Also, if as you claimed the Archaea are recent, I am curious to know what would be according to you the selective advantage for modern organisms to evolve toward a relatively little efficient metabolism such as methanogenesis? By contrast, if Archaea are older than what you said, say at least at the base of Unibacteria, they might have been happy ancient methanogens, as they evolved this metabolism at a time where the atmosphere was still not that oxygenic.

##### Author's response

The arguments for the recency of archaebacteria are published in more detail in a parallel paper [129], but still need including briefly here. I estimated the age of methanogenesis there as ~720 My. The most recent evolutionary review by an archaebacterial specialist [198] is agnostic about their age, but argues that methanogenesis is younger than archaebacteria. In principle the apparently late origin of a process can be attributed to lack of selective advantage, lack of suitable phylogenetic precursors, or its inherent mutational/mechanistic difficulty. I do not know which was most important for methanogenesis or why eubacteria never evolved it, even though some had several precursor enzymes and coenzymes. Methanogenesis is most likely to have evolved in an anaerobic place where there were high concentrations of both hydrogen and carbon dioxide. Such a niche could have been created by early protozoa with hydrogenosomes (necessarily late), by a variety of eubacteria (various ages) or inorganically (potentially very early), so evolutionary difficulties seem likely. If the ancestors of methanogens were hyperthermophilic and sulphur-compound-dependent anaerobic respirers [1], it could be that, as many hot acid environments are very poor in organic matter and not all would have appropriate sulphur compounds, there was a strong selective premium for evolving novel forms of chemoautotrophy even if relatively inefficient; perhaps archaebacteria were best placed by their hyperthermophily to invade that niche and mesophilic methanogens evolved, relatively more easily, later [1]. I think it likely that until archaebacteria evolved there were no hyperthermophiles on earth, and these niches were unexploited. This might help to explain why eubacteria never evolved methanogenesis; probably *Aquifex *and *Thermotoga *only became hyperthermophiles after they got facilitating genes from archaebacteria. For the reasons discussed in [129] and also in this paper, I think it most likely that Actinobacteria are paraphyletic (Figs 5, 7) and that neomura are sisters of or nested within Actinomycetales alone, rather than sisters of Actinobacteria as a whole, the deepest position that seems reasonably compatible with the data. But as the evidence against their holophyly is not decisive, because of the (less parsimonious) possibility of eukaryote-like character losses by deeper branching actinobacterial lineages, I originally showed them as holophyletic in Fig. 4 to emphasize that we should not yet totally reject that possibility, but have now changed this for consistency with the other figures. As I explained in [129], the question of the paraphyly or holophyly of Actinobacteria (and similarly of Endobacteria and Eurybacteria, also both arguable either way) is indeed important for the timing of several evolutionary events, notably the origins of Posibacteria, Endobacteria, and Actinobacteria. But for the reasons given there and in [1] I think it very improbable that archaebacteria can be substantially older than eukaryotes, and unlikely that they can be as old as posibacteria. But to my mind the larger uncertainty is not the age of archaebacteria, but that of Actinobacteria and Endobacteria; compared with my earlier paper [1], which regarded them both as approximately as old as most negibacterial phyla, my recent analysis tentatively suggests that both are much younger, with actinobacteria probably being still younger than endobacteria [129]. This revised interpretation greatly reduces the huge time-lag originally envisaged between the origin of Posibacteria and neomura [1], though not nearly as much as you suggest. We need group-specific palaeobiomarkers to test these inferences, and your, I think less likely, proposition.

##### Additional punctual questions about your scenarios

**- For the proteasome evolution: h**ow do we know it is easier to go from 6 to 7 fold symmetry?

##### Author's response

We don't. My argument is that the key selective advantage was expansion of the digestion chamber volume and associated reduction in accessibility of the proteolytic site to false substrates. Though 7-fold symmetry would slightly widen the chamber compared with 6-fold symmetry, probably more important was its doubling in length by differentiation into two subunits, the inner proteolytic and the outer not, and doubling the number of rings, thus greatly distancing the active sites from the entry pores. Reversing that would be harmful and mechanistically much harder. Possibly the 7-fold symmetry was an indirect consequence of either the novel interactions between the two paralogous subunits or of the novel interaction with a different ATPase cap.

**- For the loss of the OM: **you wrote p.29 :*"the negibacterial double envelope is so complex that it must have arisen only once*". Why not, but did the eye, that is so complex, arise once only? Is the "complexity" argument that compelling? With your strong knowledge of morphology could you tell us how complex are the most impressive cases of convergence within prokaryotes you could think of?

##### Author's response

Talking about '**the **eye' is wrong. Even though the molecular receptors are homologous as are some genes involved in eye development, eyes in different phyla, e.g. vertebrate eyes, arthropod compound eyes and cephalopod eyes, are structurally non-homologous with each other but homologous in detailed structure within phyla. Thus the particular structure of the vertebrate eye did evolve only once, as did that of the arthropod compound eye, but eyes in general are polyphyletic. Likewise the negibacterial double envelope is not homologous in composition or detailed structure with the so-called double membrane of the archaebacterium *Ignicoccus*. Thus a uniform pattern of complexity indicates a common origin and a different pattern of complexity a separate origin: in both eyes and bacterial envelopes. Though too little is yet known of the detailed biogenesis and modes of lipid transfer to the outer membrane/sheath of either, I predict that they will be different; there is no evidence for the negibacterial type of close membrane contact/adhesion as at Bayer's patches or porin/β-barrel OM proteins or lipoproteins in the *Ignicoccus *envelope, quite apart from the lipids being entirely different and no evidence for lipid differentiation between the CM and outer sheath. The tentative indications that vesicle budding is involved and the several fold greater 'periplasmic' volume compared with the cytoplasm is radically different from in negibacteria; if vesicle budding is involved, the *Ignicoccus *outer sheath may be more analogous to the secreted vitelline membrane of animal eggs or the secreted lipid liner of nematode eggshells than to the negibacterial OM. The fact that a known second origin of an outer 'membrane' is so utterly different in detail from the structurally very uniform negibacterial envelope strongly reinforces the likelihood of a single origin only for the latter; thus it strengthens my case, rather than weakens it as is sometimes wrongly claimed. On bacterial convergence, perhaps the eubacterial and archaebacterial flagella are the most impressive case, but when sequences became available they were readily recognized as such.

- You exposed both how mechanically easy and how historically difficult it is to loose the OM and concluded that : "*As discussed below, all posibacteria have related machinery for achieving this, which establishes their monophyly*. ", p.31. Yet, if loosing the OM is not that difficult mechanically, this argument is in fact arguable and the monophyly could be a bias against convergence. In term of parcimony, is the call for two independent losses of OM in all the history of all life much different anyway from the claim of one single loss? Both solutions seem quite parcimonious to me. For this reason, would you have any independent evidence/synapomorphy for the posibacteria/Unibacteria to support the conviction that the loss of OM happened only once? (This send us back to the general question of the independent definition of the clades, see above)

##### Author's response

A good comment on OM loss: I have inserted an explanation that, though losing the OM by murein hypertrophy would be mechanically easy, the survival of such a cell would be impaired as hypertrophy would probably drastically interfere with division, and the likely necessity of coevolving modifications to cell division could have made the evolutionary transition very difficult indeed and thus unlikely to be repeated. A synapomorphy for unibacteria plus *Thermotoga *is sn-glycerol-1-phosphate dehydrogenase (G1PDH) [18], close to your request, given that *Thermotoga *is not excluded from being sister to Posibacteria (this example was already given in the paper). But one does not need a single universal synapomorphy to exclude the convergence you suggest. Two overlapping partial synapomorphies can do that. In this case sortases and proteasomes. One sortase gene family is a synapomorphy for Posibacteria, indicating that Actinobacteria and Endobacteria did not lose the OM independently (this synapomorphy was necessarily lost during the neomuran revolution with the loss of peptidoglycan and thus is not universal in unimembranous organisms: unimembrana [129]). Proteasomes are synapomorphies for neomura and Actinomycetales, indicating that they did not lose the OM independently. Taken together they refute independent OM loss by archaebacteria and Posibacteria. A third partial synapomorphy (the acyl ester phosphatidylinositol) links eukaryotes and Actinobacteria, showing that they did not lose the OM independently. In conjunction with the 20 synapomorphies linking all neomura this is further independent evidence that archaebacteria and Posibacteria did not lose the OM separately.

- For the evidence based on the L-ring/V-ring proteins

Phylogenetic trees of these proteins would help to decide if they can or cannot easily be transferred by LGT, especially for *Aquifex *and *Thermotoga*, before being taken as evidence for a vertical history. Could you tell us more about this?

##### Author's response

It might, but I doubt it for the reasons given in my response to Referee 2. But if you think so, do it yourself.

- About the Chlorobacteria

In the figures legend, you acknowledged that this group might not be monophyletic, but in the main text you simply stated that "*Chlorobacteria *[1]*comprise filamentous 'non-sulphur' green bacteria, e.g. Chloroflexus, Oscillochloris, Chloronema and Heliothrix, which can be photoheterotrophs or photoautotrophs; colourless heterotrophs, including thermophiles (e.g. Thermomicrobium, Herpetosiphon); and chlororespirers (Dehalococcoides)*", p.64. Would you know how many published trees support or reject this grouping? Do we have a priori phylogenetic reasons to believe/not to believe in its monophyly?

##### Author's response

I did not say that they 'might not be monophyletic'. The only trees with good taxon sampling for this group are 16S rRNA trees and all show them as robustly monophyletic. I said they might be paraphyletic, which in the classical and proper nomenclature that I use is a form a monophyly [69]. Sequence trees are unrooted and thus useless to distinguish between chlorobacteria being paraphyletic or holophyletic. We need cladistic/transition analysis for robust characters that vary within the group to determine whether the root of the tree is within or beside Chlorobacteria.

- About Omp85

Your arguments are very interesting. Yet, it would be interesting to dispose of an Omp85 tree to see if its phylogenetic history matches the one of your proposed Tree of Life. This would confirm that this marker can be safely used to reason about this problem.

##### Author's response

I do not agree. It is theoretically possible for Omp85 to have been laterally transferred from one phylum to another that already had it and then to replace the existing version. A tree that accurately represented that transfer would then have the wrong topology, and thus not match my proposed tree of life. But it would be entirely wrong to conclude from it that my Omp85 argument is unreliable. Making a tree would also fail to discriminate between my argument that absence in Chlorobacteria is primitive and the theoretical possibility (the only thing that if it were true would invalidate my argument) that their common ancestor lost it. Thirdly it is highly unlikely that any single gene, whether Omp85 or anything else contains enough conserved information to correctly and robustly reconstruct the negibacterial radiation. Even the recent 31-protein tree failed to do so robustly [175]. Whatever the results we could neither be sure that it is safe to use the protein as I have or unsafe to do so. My logical reasoning is therefore independent of how closely the Omp85 tree matches Fig. 7. A sequence tree could be positively misleading if it were considered to be relevant. A tree would be interesting (a) to see how much sensible evolutionary information Omp85 sequences contain, and (b) whether there is any clear evidence for lateral transfer, but **not **for evaluating the 'safeness' of my argument. All that matters is whether it is right or wrong and the tree will not tell us. But please make one if you are sufficiently interested in the outcome

- About the ancestral complex membrane

It seems to me that regarding the origin of the negibacterial membrane, you pushed the difficulty further back in the past. It is thus difficult to evaluate if your scenario is truly parcimonious for this polarisation. Where does the ancestral negibacterial with its outer membrane come from? Was there ever a transition from one membrane to two membranes before LUCA?

##### Author's response

I think there was and that this transition marked the origin of the first cell; I explained this and how the OM may originally have evolved in detail in [31].

- About the limited impact of short term adaptation on long term phylogeny

p. 106, you claim that "*many lateral transfers seem more important for short term adaptation than for long-term phylogeny*", but I do not see why these events, even if punctual, would not strongly reshape the long term phylogeny. After all, in your paper, is not the loss of the OM one of this short term adaptation with huge taxonomical consequences?

##### Author's response

Loss of the OM was neither short-term nor necessarily adaptive. I said that many transfers seem of short-term significance, e.g. because many less-core genes seem to come and go. I did not say that no lateral gene transfers have long-term significance. Surely it would be ridiculous to claim that all lateral transfers shape long-term phylogeny. Evolution is not homogeneous in rates, magnitude or persistence of effects with respect to the different genes of the organism, but highly heterogeneous or mosaic [129]; why should lateral transfer be any different in this respect from mutations? I slightly extended this discussion by using the example of cyanobacteria to make my meaning clearer.

Minor comments:

To the reader:

Abstract, Background, first paragraph.

TCS considers the root of the tree of life as "*the most difficult problems in phylogeny*". Interestingly, Laporte in his ".Laporte, J. 2005. Is There a Single Objective, Evolutionary Tree of Life? Journal of Philosophy CII: 357–374.", recently argued along a different line, establishing that even at the smallest scale polarizing a phylogeny is incredibly problematic. This is also consistent with TCS later claims in this paper that "*establishing the root of a small part of the tree is more straightforward, yet often surprisingly difficult for organisms without plentiful fossils*", (p.3, background, first paragraph.)

##### Author's response

Saying that polarizing phylogeny is 'incredibly difficult' is an exaggeration, but not one that in any way contradicts my assertion that positioning the root and establishing the properties of the most ancient cells is the most difficult phylogenetic problem. But I prefer to solve a problem rather that to philosophize about why it is difficult.

##### In "The primacy of transition analysis, p.6, 4 paragraph"

"*Search for congruence among multiple lines of evidence – the more diverse the better and resolving apparent contradictions by weighing up the evidence is not special to evolutionary biology but fundamental to all science*." I think this is arguable. In a paper to be published soon in Philosophy and Biology, G. McOuat illustrates how eastern science can go for a different objective: highlighting conflict and incongruence as major features of scientific knowledge instead of stressing on congruence. K. Popper stressed on falsification not confirmation to realize scientific progress. T. Kuhn and P. Feyerabend would also argue that, deeply, science does not proceed so much by confirmation, but more by revolutions and shifts.

##### Author's response

Anything is arguable if you are sufficiently philosophically inclined. Congruence and incongruence are two inseparable sides of a coin. I have little respect for Feyerabend's approach, but think that a realistic appreciation on how science actually progresses involves elements of both Popper's and Kuhn's perspectives, which are not as contradictory as you imply. Thus, using Kuhnian language, we could call the shift, which I hope is underway, of considering the root to be between neomura and eubacteria to accepting it among negibacteria a paradigm shift, because it affects our perspectives in so many ways and also meets the usual intense resistance associated with such a major shift. But the proper way to effect such a shift is not philosophic discussion but by evaluating the evidence critically and in detail. In evolutionary biology both refutation and confirmation have a role. Popper's emphasis on refutation and denial of the validity of scientific proof (in contrast to mathematical proof) stems from his obsession with general theories. But phylogeny is particular and historical not general. That reptiles were ancestral to birds and mammals, or prokaryotes were ancestral to eukaryotes are not general theories, but specific suggestions that specific unique historical events actually happened. For unique historical events, particular pieces of evidence can logically confirm or prove a particular happening; Popper's argument for the logical asymmetry of proof and disproof relates only to general/universal theories, where single novel instances may disprove previously well-corroborated and accepted general theories. But for historical events if we have sufficient information we can prove some things beyond question, such as what were the historical ancestors of a new allopolyploid species like *Primula kewensis *or that Queen Victoria existed and who her children were.

p. 107: TCS considers ref [162] as unduly pessimistic. As one of its author, I feel that many of the conceptual problems listed in this reference (diaphonia, criterion evaluation, circular reasoning and palaisma) are still present, even in TCS admirable work. In terms of optimism/pessimism, future studies will tell if the congruence defended in the present transition analyses, a major progress for the debate about the tree of life, can be considered as the solution or if the debate will keep going on. I bet it will.

##### Author's response

I do not think my reasoning circular, and you have not pointed out anything specifically that is. Your final prediction is historically solidly based: there are still flat-earthers, creationists, and people who believe in fairies.

Minor comments:

To the author.

##### In "The primacy of transition analysis, first paragraph"

It is not common practice in cladistics to use a grade. I do not mind it, but I do not know if cladists would enjoy your example of "reptiles", and give you their full support there.

##### Author's response

Cladistic reasoning in phylogeny, which I use and highly value (in that sense I am a cladist), actually 'uses' both clades and grades when referring to ancestral and derived conditions. What you mean is that some cladists hate paraphyletic groups in formal taxonomy. As explained elsewhere [69], I think all reasons they have given for this aversion are scientifically unsound; they reflect confusion between the purposes of phylogeny and taxonomy. But the validity of my reptile example is entirely independent of whether you wish to classify reptiles as a taxon (as I would) or not (which some, not all, cladists prefer).

##### In "Paralogue rooting failed clearly to root the tree"

You don't have to, but you might be willing to comment on Zhaxybayeva O, Lapierre P, Gogarten JP., Ancient gene duplications and the root(s) of the tree of life., Protoplasma. 2005 Dec;227(1):53–64. Epub 2005 Dec 30.

##### Author's response

This paper [116] by one of the original inventors of paralogue rooting agrees with me that it has **not **solved the problem. They made trees for 17 universal paralogue pairs; nine put the root between archaebacteria and eubacteria; seven put it within eubacteria (which I consider correct) and one put it within archaebacteria. They explicitly recognized that all trees showing the root between neomura and eubacteria could be affected by long-branch artefacts, as I earlier suggested [1]. As I had [1], they draw attention to the fact that the insertion in the neomuran catalytic subunit only of the vacuolar type ATPase strongly indicates that the neomuran state is derived compared with the eubacterial state. As I also pointed out [1], this polarizes the tree from the eubacterial state to the neomuran state for this enzyme. This polarization clearly contradicts the rooting shown by the tree itself, which is between neomura and eubacteria. Thus the same molecule gives two contradictory answers depending whether you use tree rooting logic (necessarily subject to long-branch artefacts) or discrete character molecular cladistic/polarization logic (unaffected by long branching). I think the only reasonable way of resolving this contradiction is to accept that the tree is misrooted by extremely severe long-branch attraction, exactly as I explained before [1] (those long bare stems stick out like a sore thumb) and that the polarization by the insertion from eubacteria to neomura is correct; thus eubacteria are ancestral to neomura. However only four of the other eight enzymes showing the root between archaebacteria and eubacteria, rather than within eubacteria, are ribosome- or transcription-related, which I argued should have been subject to strong quantum evolution [1] specifically in the ancestral neomuran. But there is no reason why some other proteins should not have similar long-branch problems, as many paralogues are deeply divergent and thus have a highly stretched stem on paralogue trees. These new conflicting results among different paralogues reinforce the necessity of using transition polarization to resolve the conflict.

##### In "HslV to proteasome differentiation polarizes the evolutionary transition, 7 paragraph, p.23"

To conclude your scenario about HslV evolution, you say that "Thus mechanistic, selective, and ***phylogenetic*** arguments all unambiguously polarize the direction of evolution". You should remove "phylogenetic". I do not think it is tested, since it is not possible to do a phylogenetic tree with HslV, the alpha and the beta subunits, as you explained after, p. 25, 2^nd ^paragraph.

##### Author's response

I disagree. You construe 'phylogenetic' too narrowly. The term was invented a century before anyone did sequence trees or invented electronic computers. There is much phylogenetic evidence other than sequence trees; phylogenetic arguments entirely independent of sequence trees can often be stronger than the conclusions of a sequence tree.

The notion of "monophyletic grade" is unknown to me (p. 76). You could rephrase that for people with a cladist eye.

##### Author's response

The meaning should be obvious. Grades can be ancestral or derived. Some grades are monophyletic (either paraphyletic, like prokaryotes or fish, or holophyletic, like tetrapods) others are polyphyletic (like testate amoebae or legless reptiles). The former are acceptable as taxa, the latter are not; some with a 'cladist's eye' have almost forgotten the important distinction between paraphyly and polyphyly and lump both as 'non-monophyletic'. Classically [189], and to me, monophyly includes both paraphyly and holophyly [69]; it originated to contrast with polyphyly. Using all four terms appropriately is more precise and informative than using just two: 'monophyletic' sensu Hennig (= holophyletic) and 'non-monophyletic' (sensu many cladists = paraphyletic plus polyphyletic). By attempting to change monophyly to mean holophyly alone Hennig destroyed its original purpose [189], sowing immense confusion (two conflicting meanings now for monophyly) that still haunts us.
